# Visible-Light Mediated Oxidative Fragmentation of Ethers and Acetals by Means of Fe(III) Catalysis

**DOI:** 10.1021/acs.orglett.2c00231

**Published:** 2022-02-22

**Authors:** Rickard Lindroth, Alica Ondrejková, Carl-Johan Wallentin

**Affiliations:** † Department of Chemistry and Molecular Biology, University of Gothenburg, SE-412 96 Gothenburg, Sweden

## Abstract

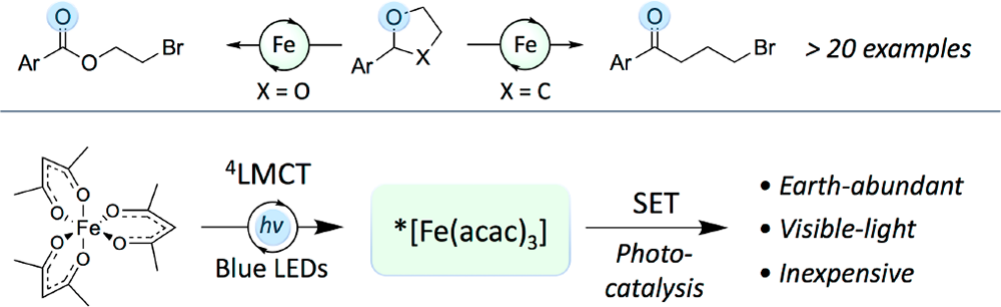

A new method employing iron(III) acetylacetonate along with visible light is described to effect oxidative ring opening of cyclic ethers and acetals with unparalleled efficiency. The method allows for a photocatalytic radical chemistry approach to functionalize relatively inert cyclic ethers into useful synthetic intermediates. The methodology sheds further light on the use of underexplored iron complexes in visible-light photochemical contexts and illustrates that simple Fe(III) complexes can initiate redox processes from ^4^LMCT excited states.

Cyclic ethers are used in most scientific disciplines utilizing organic compounds. Despite being ubiquitous structural moieties, synthetically useful transformations employing these structural features as synthons are scarce. Attempts at achieving oxidative ring opening of cyclic ethers has essentially been limited to epoxides and oxetanes, relying on strain relief mediating the transformations.^1−3^ Among the few examples known for nonstrained cyclic ethers, e.g. tetrahydrofurans, harsh conditions such as aqueous molecular bromine has been used to oxidatively ring-open THF to give 4-hydroxybutanal in only 20% yield (Scheme 1b).^4^ A more recent paper by Leadbeater and co-workers pursued the transformation of 2-phenyltetrahydrofurans into 4-hydroxy-1-phenylbutane-1-ones by employing oxoammonium salts as oxidants (Scheme 1b).^3^ The transformation is achieved by a hydride abstraction generating a stabilized carbocation. The authors concluded that the cationic intermediates were too stable resulting in multiple reaction pathways, e.g. polymerizations, giving poor yields with the best example reaching only 45% yield. Moreover, the method was unable to use any other nucleophile than water due to nucleophile oxidation by the oxoammonium cation. These examples illustrate the typical difficulties associated with oxidative approaches for fragmentative diversification of the ether functionality.

**Scheme 1:**
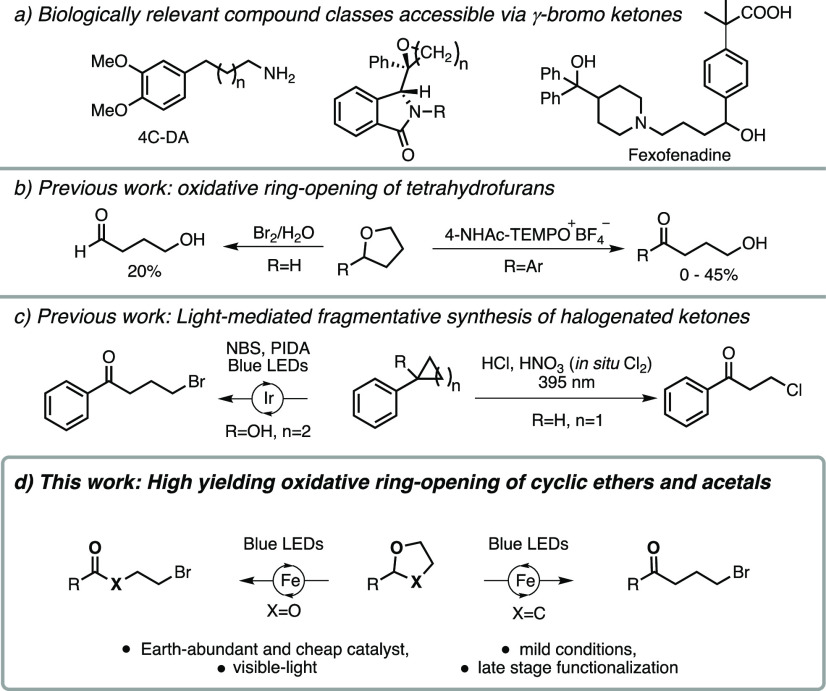
Oxidative Ring Opening of THF-Core and Methods of Halogenated Ketone Syntheses

The past decade has seen a surge in catalytic visible-light mediated methodologies demonstrating its success in achieving novel radical transformations under mild conditions.^5−7^ As part of our ongoing research activities, we aimed to explore if photoredox catalysis could provide opportunities for developing a reliable and robust approach engaging ether functionalities oxidatively, and thereby generating structural features of broader synthetic value. Our initial ambition was to target the generation of γ-bromo ketones, which are valuable building blocks for various chemical transformations and utilized in the syntheses of many biologically relevant compound classes (Scheme 1a).^8−10^ Recent work from the Knowles^11^ and Zhu^12^ groups provide elegant examples of PCET mediated fragmentative functionalization of cyclic alcohols to access γ- or δ-bromo ketones.^13−15^ Also noteworthy is the work by Almqvist, König and co-workers that recently disclosed the generation of β-chloroketones via a light-mediated oxidative fragmentation using aryl cyclopropanes as starting material (Scheme 1c).^16^

We hypothesized that hydrogen atom transfer, HAT, not only could be used for chemoselective generation of carbon-centered radicals from ethers, but that such an approach toward oxidative fragmentation might overcome the limited substrate scope and competing polymerization as previously reported for methods involving polar pathways. Herein, we report the use of simple Fe(acac)_3_ as an inexpensive, efficient, and earth abundant based catalyst in a visible-light driven oxidative ring opening of tetrahydrofurans, tetrahydropyrans, and acetals to give synthetically useful bromo-substituted ketones or esters (Scheme 1d). Recently, other noteworthy examples in photoredox catalysis reported the use of simple catalysts based on iron.^17−23^ To the best of our knowledge, the present method constitutes the first high-yielding oxidative ring opening of nonstrained cyclic ethers and, furthermore, the process is presumably initiated via an unprecedented SET from an excited state Fe(III) species by an initial ^4^LMCT absorption.

In our initial investigation we explored the use of Ru(bpy)_3_(PF_6_)_2_ as a photocatalyst to oxidatively ring-open 2-(4-chlorophenyl)tetrahydrofuran (**1aa**) to the target 4-bromo-1-(4-chlorophenyl)butan-1-one (**1a**). To our dismay, these conditions provided irreproducible results, ranging from 0% to quantitative yields based on ^1^H NMR (Table 1, entry 1). Even more surprisingly, we found the reaction to behave similarly in our control experiment without any catalyst present. We suspected that trace metal impurities possibly could be responsible for the irreproducibility issues, which prompted us to screen various transition metal additives (see Supporting Information (SI)). Indeed, when using new glassware or glassware cleaned with aqua regia, conversion to the product was reproducible (Table 1, entry 2). We were pleased to find that **1a** was consistently formed in 90% yield using 1 mol % of Fe(acac)_3_ and 3 equiv of BrCCl_3_ in dichloroethane irradiated with blue LEDs (Table 1, entry 3).

**Table 1:**
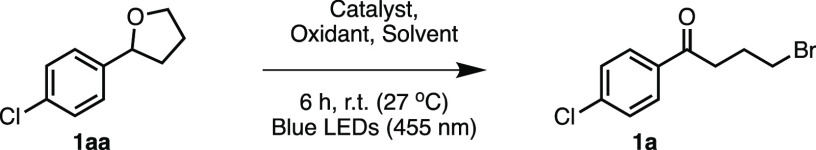
Deviation from Standard Conditions

Entry	Catalyst	BrCCl_3_	Solvent	Yield (%)^a^
1^b^	Ru(bpy)_3_(PF_6_)_2_ 1–5 mol %	2–10 equiv	Solvents	0-quant
2	Ru(bpy)_3_(PF_6_)_2_ 1 mol %	3 equiv	DCE	31 (full conv^c^)^g^
**3**	**Fe(acac)**_**3**_**1** **mol %**	**3 equiv**	**DCE**	**89 (90**^d^**)**
4	Fe(acac)_3_ 1 mol %	CBr_4_, 3 equiv	DCE	64
5	FeBr_3_ 1 mol %	3 equiv	DCE	55
6^e^	Fe(acac)_3_ 1 mol %	3 equiv	DCE	Trace
7^f^	Fe(acac)_3_ 1 mol %	3 equiv	DCE	No reaction
8^g^	No catalyst	3 equiv	DCE	Trace

a Isolated yields conducted at 0.1 mmol scale.

b Note, irreproducible yields were consistently obtained also when keeping all parameters constant. Yields were determined by ^1^H NMR using dimethyl sulfone or ethylene carbonate as internal standard.

c Reaction run for 18 h.

d Average isolated yield of two runs at 0.2 mmol scale.

e Heat control (80 °C).

f Control experiment in the dark.

g Reaction conducted in either a brand new vial or a vial cleaned with aqua regia.

It should be pointed out that these conditions provided the product much more efficiently than the corresponding conditions using Ru(bpy)_3_(PF_6_)_2_ as catalysts (6 h and 89% yield vs 6 h and 31% yield). It should be noted that a catalyst loading of 0.1 mol % also promotes the reaction with a slightly diminished efficacy (SI, Table 1, entry 12), whereas decreasing the loading even further down to ppm levels generates conditions that provide 50–60% conversion when irradiated for 18 h. These results may shed some light on recently published methods in which BrCCl_3_ is claimed to be engaged in photo-mediated processes under catalyst-free conditions.^24,25^ Our control experiments show that the reaction is not thermally promoted and both Fe(acac)_3_ and light are essential for the reaction to progress (Table 1, entries 7–8).

With optimized conditions at hand we started our scope exploration by varying the aromatic moiety of the tetrahydrofuran derivative. As can be seen in Scheme 2, the method is compatible with a vast range of electronic properties associated with the aromatic functionality (**2a**–**2k**). Going from methoxy (**2d**) to a nitrile (**2c**) substituent in the *para*-position practically does not affect the efficiency of the reaction giving a 79% and 81% yield, respectively. The reaction also progressed well with a sterically hindered isopropyl substituent in the *ortho*-position (**2f**, 92%). Expanding the scope to also include electron-rich N-heteroaromatics typically provided trichloromethyl functionalized THF derivatives, as exemplified by the formation of **2l**, along with unreacted starting material. These observations can be rationalized by the basic nature of these moieties, which are localized in close proximity to the β-carbon of the THF functionality. This setup can possibly mediate an elimination of the so formed cationic intermediate (Scheme 6) providing the corresponding 2,3-dihydrofuran derivatives.

**Scheme 2:**
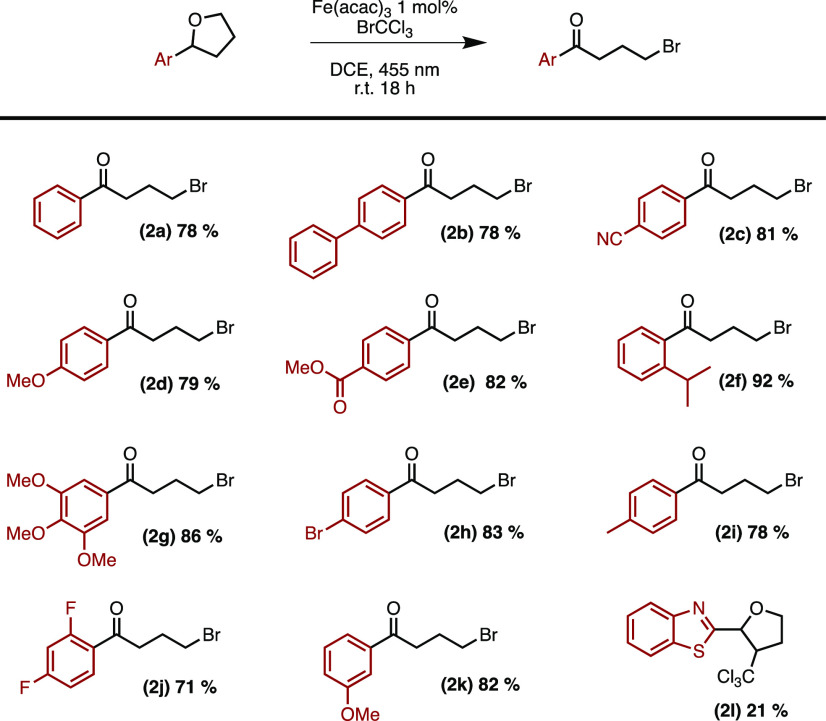
Scope of Aromatic Moiety of THF-Derivative Reactions were conducted at 0.2 mmol scale and 0.1 mol/dm^3^, 3 eq. BrCCl_3_ and 1 mol % Fe(acac)_3_. Yields are reported as average isolated yield of two runs, except for (**2j**) with only one run.

Next we turned to investigate congeners to the THF functionality (Scheme 3a). Introducing a methyl group in the 3-position of the THF-core gave an exceptionally clean reaction in 95% yield (**3a**). Enlarging the ring size to a tetrahydropyran was also compatible with the reaction conditions, yielding 5-bromo-1-phenylpentan-1-one (**3b**) in 84% yield. Surprisingly, when the dioxane core (**3cc**) was subjected to the reaction conditions, no product or only traces could be detected with close to total recovery of the starting material.

**Scheme 3:**
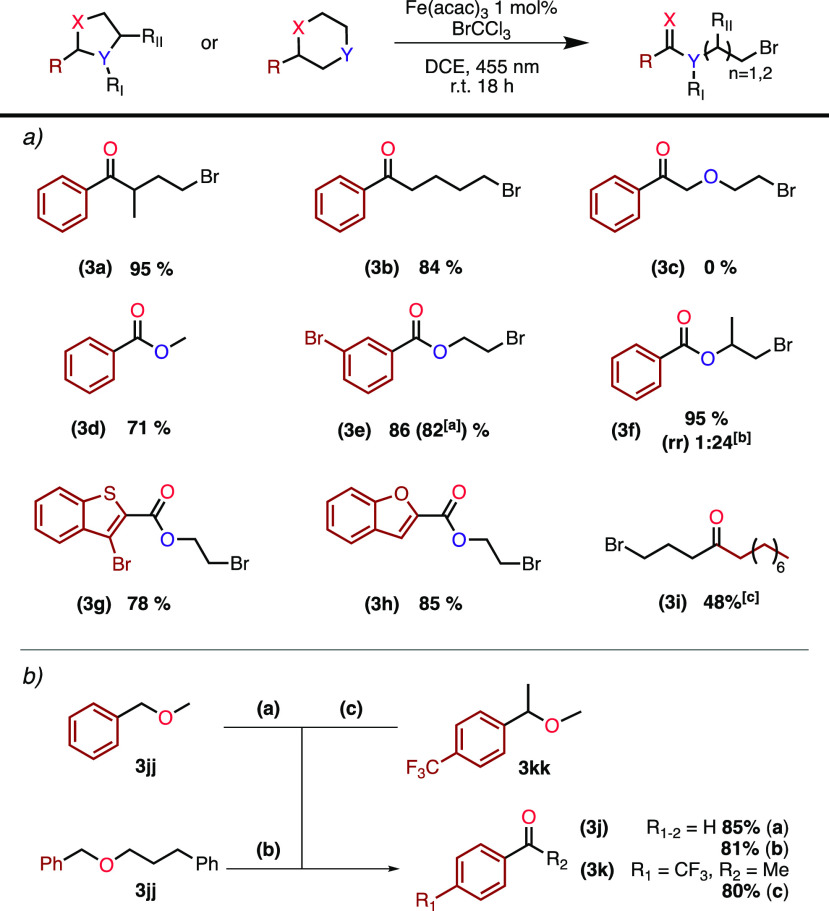
Scope of Ether and Acetal Reactions were conducted at 0.2 mmol scale and 0.1 mol/dm^3^, 3 equiv of BrCCl_3_ and 1 mol % Fe(acac)_3_. Yields are reported as average isolated yield of two runs. Reaction conducted at 1 mmol scale. Regioisomeric ratio. Percentage refers to conversion as determined by ^1^H NMR; 10 equiv of BrCCl_3_ were used.

Acetals have previously been reported to oxidatively convert to esters under various conditions, and we consequently wondered if such a transformation would also be accessible with our iron-catalyzed photodriven conditions.^26−29^ To our delight, benzaldehyde dimethyl acetal neatly transformed to methyl benzoate (**3d**) in good yield (71%). Cyclic acetals, 1,3-dioxolanes, were all converted to the expected products with high yields ranging from 78% to 95% (**3e**–**3h**). 2-(3-Bromophenyl)-1,3-dioxolane was subjected to the reaction conditions on a 1 mmol scale, resulting in only a minor decrease in yield, providing 82% as compared to 86% (**3e**). When 4-methyl-2-phenyl-1,3-dioxolane was subjected to the reaction conditions, an inseparable mixture of two regioisomers was formed in a 1:24 ratio with a total yield of 95% (**3f**). We continued examining the compatibility with heteroaromatic functionalities of our system on cyclic acetals and contentedly found that benzo[*b*]thiophene- and benzofuran cores are successfully tolerated providing **3g** and **3h** in 78% and 85% yield, respectively. Furthermore, applying more forcing conditions by increasing the equivalents of BrCCl_3_ also converted a nonaromatic THF-derivative with a pendant alkyl chain to the γ-bromo ketone.

Next, exploring the engagement of acyclic ethers provides a method for oxidative dealkylative fragmentation to yield aldehydes or ketones (Scheme 3b). Benzylmethyl ether and (3-(benzyloxy)propyl)benzene both converted to benzaldehyde (**3j**) in good yields (85% and 81% respectively). The latter result, showing selectivity for the benzyl ether hydrogen over the benzylic hydrogen, sheds light on the possibility of brominative deprotection of benzyl ethers. Lastly, introducing a methyl group on the benzylic position gives access to the corresponding acetophenone (**3k**) in 80% yield.

To further demonstrate the utility of our system, we addressed the production of a key intermediate in the synthesis of H1 receptor antagonist fexofenadine.^10^ The key intermediate is a 4-bromo-1-phenylbutane-1-one with an isobutyronitrile group in the *para*-position. We envisioned a two-step synthesis of this compound starting with the production of the corresponding tetrahydrofuran precursor followed by our iron-catalyzed visible-light driven reaction (Scheme 4). The one-pot Heck and hydrogenation reaction described by Evans and co-workers gave the desired 2-methyl-2(4-(tetrahydrofuran-2-yl)phenyl)propanenitrile (**4aa**) in a satisfactory 68% yield.^30^ Next, subjected to our conditions the key intermediate (**4a**) was formed in 82% yield giving a total isolated yield of 56%, which should be compared to 53% yield over 3 steps as previously reported.^10^

**Scheme 4:**
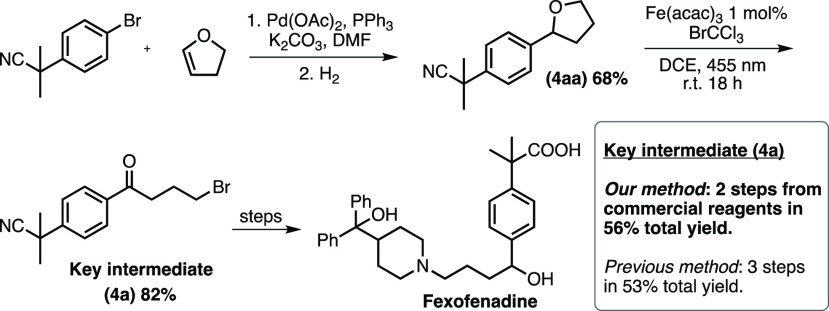
Synthesis of Key Intermediate to Fexofenadine

To gain some insight into the mechanism of this reaction we carried out a set of reactions under different conditions to probe for the role of Fe(acac)_3_. It has been described that Fe(acac)_3_ under irradiation of visible light undergoes ^4^LMCT resulting in release of an acac radical (Scheme 5).^31,32^ One conceiving mechanism could be that an extruded acac radical might act as the hydrogen atom acceptor, initiating a propagation based generation of the product. To probe this possibility two experiments were devised (*i–ii* in Scheme 5).

**Scheme 5:**
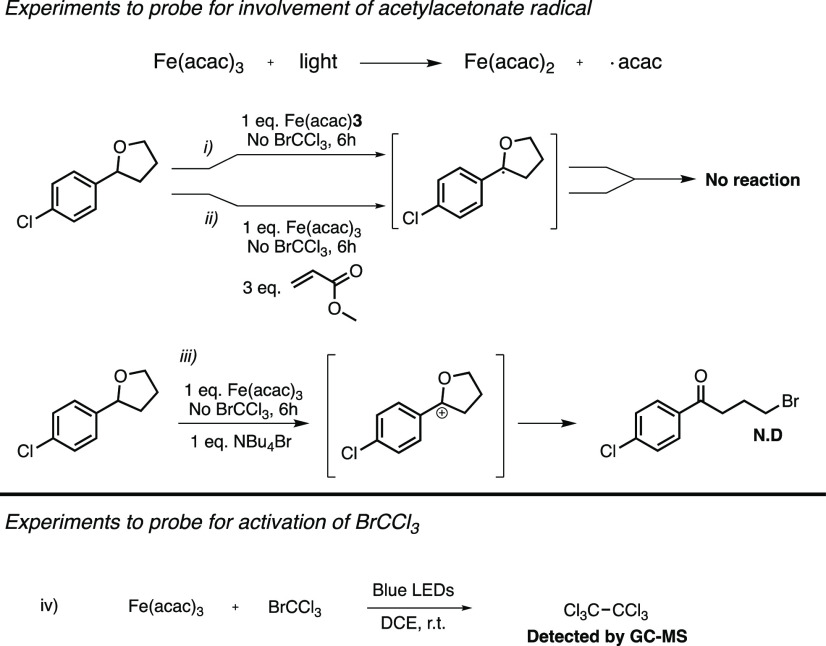
Mechanistic Experiments of acac Radical Involvement

In experiment (*i*), equimolar amounts of Fe(acac)_3_ and **1aa** were irradiated under optimized conditions. If acac radicals were to be extruded, degradation of **1aa** with potential formation of dimers or the dihydrofuran derivative would be expected. However, after 6 h of irradiation, no reaction could be observed. Experiment (*ii*) served the same purpose, to probe the formation of acac radicals. By adding an electrophilic reaction partner an opportunity is provided to more efficiently engage the hypothetical THF radical in a Giese reaction. However, as for the previous experiment, no reaction occurred. Experiment (*iii*) served the purpose of probing for any potential ionic intermediates formed in the absence of BrCCl_3_. Again, only starting material was observed after 6 h of irradiation. Taken together, these findings are in agreement with previous studies that reported on the high photostability of Fe(acac)_3_.^33^ We therefore find it unlikely that acac radicals initiate the reaction.

Instead, we turned our attention to Fe(acac)_3_ as an activator of BrCCl_3_. Because Fe(acac)_3_ does not fluoresce within the visible region, the typical Stern–Volmer quenching studies were not a viable option to probe such activation. That said, if Fe(acac)_3_ could induce a mesolytic cleavage of BrCCl_3_, this process should be able to initiate a dimerization of the so formed trichloromethyl radicals. Indeed, when irradiating an equimolar solution of BrCCl_3_ and Fe(acac)_3_, hexachloroethane was detected as a major constituent by GC-MS (*iv* in Scheme 5). This result strongly suggests that *[Fe(acac)_3_] is quenched by BrCCl_3_ either by energy transfer or by SET. Furthermore, the reaction was almost completely impeded by addition of TEMPO.

Besides quenching of *[Fe(acac)_3_] by BrCCl_3_, there is a possibility of forming an EDA-complex between Fe(acac)_3_ and BrCCl_3_ for which UV–vis titration experiments were conducted, but these ruled out any potential absorption complex formation (see SI). Energy transfer based initiation via homolytic cleavage can also be ruled out due to the energy discrepancy between the relaxed ^4^LMCT state of *[Fe(acac)_3_] (220 kJ/mol)^34^ and that of the ^1^[BrCCl_3_] → ^3^[BrCCl_3_] transition (571 kJ/mol; see SI). On the other hand, the estimated redox potential of the relaxed ^4^LMCT state of *[Fe(acac)_3_] (*E*_1/2_^red^ = −0.68 V vs SCE)^34,35^ clearly provides an exergonic pathway for SET based quenching with BrCCl_3_ (*E*_1/2_^red^ = −0.18 V vs SCE).^5^

The absorption characteristics and the excited state dynamics of Fe(acac)_3_ have been thoroughly studied experimentally.^33^ This study together with early work by Wilkinson and Farmilo^34^ clearly indicates that Fe(acac)_3_ harbors at least two metal centered excited states lower in energy than the initial vibrationally relaxed ^4^LMCT state of *[Fe(acac)_3_] (λ_max_ = 440 nm). The estimated excited state redox potential for one of these states do allow for an exergonic SET to BrCCl_3_ (see SI Figure S2). Upon excitation at 440 nm, Fe(acac)_3_ is known to regain the absorption characteristics of its fully decayed ground state after ca. 60 ps.^33^ As such, Fe(acac)_3_ decay back to its ground state roughly 1 order of magnitude faster than [Fe(bpy)_3_]^2+^, a complex known to efficiently initiate propagation based processes via the excited state.^18^

With these experimental results at hand, we propose a mechanism starting with Fe(acac)_3_ absorbing a photon resulting in a ^4^LMCT state of *[Fe(acac)_3_] (Scheme 6). Oxidative quenching of the excited catalyst by BrCCl_3_ causes mesolytic cleavage to give a trichloromethyl radical and a bromide ion. The trichloromethyl radical abstracts the α-hydrogen, forming chloroform. From here a propagation mechanism starts where the radical intermediate abstracts a bromine from BrCCl_3_ forming another trichloromethyl radical. The bromine intermediate fragmentizes to a stabilized carbocation intermediate that ring-opens irreversibly by action of the bromide ion. The [Fe(acac)_3_]^+^ formed is highly oxidizing (+1.60 V vs SCE)^35^ and, from a thermodynamic perspective, capable of oxidizing the nucleophilic radical (<+0.16 V vs SCE).^36^ This SET event closes the catalytic cycle for Fe(acac)_3_. There is also the possibility of the so formed electron-rich radical to reduce Fe(acac)_3_ to Fe(II) from which another mode of initiation could act in parallel.

**Scheme 6:**
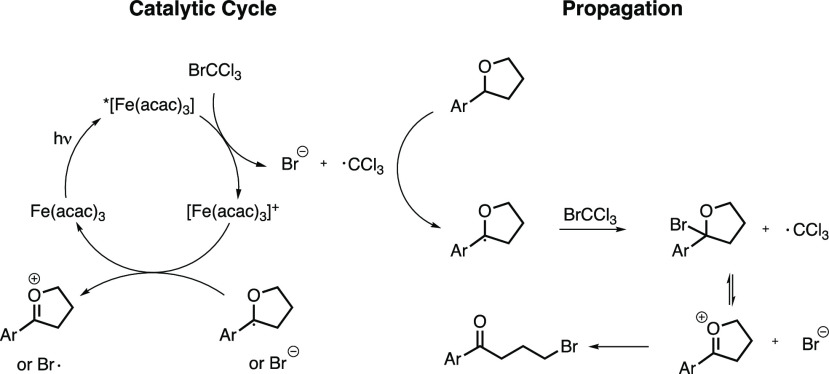
Proposed Mechanism

In summary we have developed an efficient method for the oxidative ring opening of cyclic ethers as well as acetals with good to excellent yields. The method employs the novel reactivity of Fe(acac)_3_ in conjunction with visible light. There is still some uncertainties regarding the detailed mechanism, and a more in-depth understanding of light-induced iron chemistry is needed.

## Supporting Information Available

Synthetic procedures and characterization data (PDF)
