# Supplementary material for: Visible-Light Mediated Oxidative Fragmentation of Ethers and Acetals by Means of Fe(III) Catalysis
Source: Org Lett. 2022 Feb 22;24(8):1662–7. doi: 10.1021/acs.orglett.2c00231 (PMC8902804; doi:10.1021/acs.orglett.2c00231)
Supplement: Supplementary file 1 — ol2c00231_si_001.pdf [file ol2c00231_si_001.pdf]

Supporting Information

Visible-Light Mediated Oxidative Fragmentation of Ethers and Acetals by Means of Fe(III) Catalysis

Rickard Lindroth[a], Alica Ondrejková[a], Carl-Johan Wallentin[a]*

[a]Department of Chemistry and Molecular Biology, University of Gothenburg, Kemigården 4, 412 96, Gothenburg, Sweden

*Email: carl.wallentin@chem.gu.se

Table of contents

General information ____ **S-3**

Deviation from standard conditions ____ **S-4**

Screening of metal additives ____ **S-5**

Synthesis of starting materials ____ **S-6**

- **Method I - THF-derivatives** ____ S-6
- **Method II – THF, THP and dioxane-derivatives** ____ S-6

Characterization data and preparation of starting materials ____ **S-7**

Experimental procedure for photoreaction ____ **S-14**

Characterization data for photoproducts ____ **S-15**

Mechanism – Experiments and Calculations ____ **S-20**

- **UV-Vis** ____ S-20
- **Reactions** ____ S-21
- **Calculations on $BrCCl_3$** ____ S-22
- **Jablonski diagram** ____ S-22

References ____ **S-23**

Appendix – Experimental NMR-Spectra ____ **S-24**

General information

All chemicals were purchased from Sigma-Aldrich and VWR. All chemicals were used as received (exception being 2-(3-bromophenyl)-1,3-dioxolane which was purified by precipitating the aldehyde impurity as its bisulfite adduct). All reactions were performed under an atmosphere of argon using Schlenck techniques. TLC plates (Silica gel 60 F_{254} coated on aluminum plates purchased from Sigma-Aldrich and VWR) were visualized by either UV light (254 nm), Seebach's stain (superior for THF-derivatives) or $KMnO_4$ solution. Flash column chromatography was performed both manually (using Silica gel 40-63 µm from VWR) and with Büchi Pure C-810 Flash (using Büchi FlashPure EcoFlex cartridges of either 50 µm or 20 µm).
GC-MS(EI) was performed on Agilent 19091S-433 gas chromatograph with an Agilent 5977E MSD detector. Separations were performed on HP-5MS Phenyl Methyl Silox of 30 m x 250 µm x 0.25 µm column.
LC-(HR)MS was performed on a Waters Acquity™ UPLC coupled with a SYNAPT G2-Si Mass Spectrometer using a LockSpray (Zspray™) ESI ionization source.
IR-spectra were recorded on a Bruker FT-IR Spectrometer INVENIO®.
A Büchi® Melting Point B-545 was used to determine the melting points.
^{1}H-NMR and ^{13}C-NMR spectra were recorded on a Varian NMR 400 or Bruker Avance III HD 800 MHz spectrometer. Chemical shifts (δ) are reported in ppm relative to the residual solvent peak. Splitting patterns are indicated as (s) singlet, (d) doublet, (dd) doublet of doublets, (ddd) doublet of doublet of doublets, (dddd) doublet of doublet of doublet of doublets, (t) triplet, (tt) triplet of triplets, (dt) doublet of triplets), (td) triplet of doublets, (dtd) doublet of triplet of doublets, (ddt) doublet of doublet of triplets, (q) quartet, (dq) doublet of quartets, (qt) quartet of triplet, (dqd) doublet of quartet of doublets, (quint) quintet, (sext) sextet, (hept) heptet, (m) multiplet. Coupling constants (*J*) are reported in Hertz (Hz).
Photoreactions were conducted in Biotage® Microwave Reaction Vials placed in EvoluChem™ PhotoRedOx Box By HepatoChem equipped with an EvoluChem LED 18 W (P201-18-2, 450-455 nm) as irradiation source and was used without any filter. Emission spectrum can be found at https://www.hepatochem.com/photoreactors-leds-accessories/led-evoluchem.

Deviation from standard conditions

The reactions were prepared according to the procedure described in the section *Experimental procedure for photoreaction*.

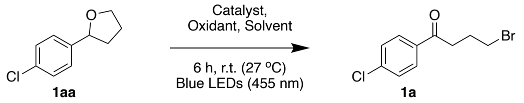

Entry	Catalyst	$BrCCl_3$	Solvent	Yield (%)[a]
1[b]	$Ru(bpy)_3(PF_6)_2$ (1-5 mol%)	2-10 eq.	Solvents	0-quant
2	$Ru(bpy)_3(PF_6)_2$ (1 mol%)	3 eq.	DCE	31(full conv.[c])[g]
3	$Fe(acac)_3$ (1 mol%)	5 eq.	DCE	76
**4**	**$Fe(acac)_3$ (1 mol%)**	**3 eq.**	**DCE**	**89 (90[d])**
5	$Fe(acac)_3$ (1 mol%)	1.2 eq.	DCE	59
6	$Fe(acac)_3$ (1 mol%)	0 eq.	DCE	No reaction
7	$Fe(acac)_3$ (1 mol%)	3 eq.	Toluene	52
8	$Fe(acac)_3$ (1 mol%)	3 eq.	MeCN	41
9	$Fe(acac)_3$ (1 mol%)	3 eq.	EtOAc	66
10	$Fe(acac)_3$ (1 mol%)	3 eq.	DMF	Trace
11	$Fe(acac)_3$ (1 mol%)	CBr_4, 3 eq.	DCE	64
12	$Fe(acac)_3$ (0.1 mol%)	3 eq.	DCE	64
13	$FeBr_3$ (1 mol%)	3 eq.	DCE	55
14[e]	$Fe(acac)_3$ (1 mol%)	3 eq.	DCE	Trace
15[f]	$Fe(acac)_3$ (1 mol%)	3 eq.	DCE	No reaction
16[g]	No catalyst	3 eq.	DCE	Trace

Table 1. [a] Isolated yields conducted at 0.1 mmol scale. [b] Note, irreproducible yields were consistently obtained also when keeping all parameters constant. Yields were determined by ^{1}H-NMR using dimethyl sulfone or ethylene carbonate as internal standard. [c] Reaction run for 18 h. [d] Average isolated yield of two runs at 0.2 mmol scale. [e] Heat control (80 °C). [f] Control experiment in the dark. [g] Reaction conducted either in a brand new vial or a vial cleaned with aqua regia.

Screening of metal additives

The reactions were prepared according to the procedure described in the section *Experimental procedure for photoreaction*. The reactions were analyzed by taking 0.1 ml of crude reaction mixture and diluted with 0.6 ml $CDCl_3$ followed by recording ^{1}H-NMR, no prior work-up. Results are summarized below.

No.	Metal additive	Conversion (%)
1	$Ce(SO_4)_2$	0
2	$Co(ClO_4)_2$	100
3	$Sc(OTf)_3$	0
4	$Pd(CF_3COO)_2$	0
5	$Gd(OTf)_3$	0
6	$La(OTf)_3$	0
7	$Bi(OTf)_3$	0
8	$AgBF_4$	0
9	$Rh_2(CH_3COO)_4$	0
10	$MnCl_2$	0
11	$RuCl_3$	0
12	$Ni(acac)_2$	29
13	CuCl	17
14	$CuCl_2$	0
15	$Fe(acac)_3$	100

Table 2. 1 mol% of the metal additive and 2-(4-chlorophenyl)tetrahydrofuran (**1aa**) were used in each reaction and run for 6 hours. All vials and stir bars were carefully cleaned with aqua regia before reaction.

To double-check that $Fe(acac)_3$ and $Co(ClO_4)_2$ are active catalysts for the reaction experiments were conducted again for these two metal complexes in brand new vials. In case of $Fe(acac)_3$ the results were always reproducible while for $Co(ClO_4)_2$ slightly diminished yields were sometimes obtained. Hence, we concluded that $Fe(acac)_3$ was the most efficient catalyst and continued exploring it in our system.

Synthesis of starting materials

Two general methods have been used to prepare a large portion of the starting materials. The specific syntheses of starting materials beyond these two methods are described in the following section together with the characterization data for that compound.

Method I - THF-derivatives

The corresponding 4-oxo-4-phenylbutyric acid (5 mmol) was added portion wise to a suspension of $LiAlH_4$ (4 eq.) in Et_2O (50 ml) at 0 °C. Reaction was then stirred at r.t until completed as judged by TLC. Work-up procedure according to the Fieser protocol. Almost quantitative yields of the diols were obtained, and the diols were used in the next step without further purification.
The cyclization of the diols to tetrahydrofurans was conducted as described in[1]. No purification on silica was necessary.

Method II – THF, THP and dioxane-derivatives

Cyclic ethers were synthesized according to procedure in[2]. Oven dried Biotage MW vial (20 ml) was charged with the boronic acid (2 mmol), $Ni(acac)_2$ (10 mol%, 51 mg), PPh_3 (10 mol%, 52 mg), K_3PO_4 (1 eq., 425 mg) and THF (anhydrous, 12 ml). The vial was capped and reaction mixture sparged with argon for 10 min. Then DTBP (1.2 eq., 0.44 ml) was added in one portion and the reaction mixture heated to 100 °C overnight. After cooled to r.t the reaction was diluted with 20 ml $HCl_{(aq)}$ (2M)[1] and extracted 3 times with 20 ml Et_2O, dried over Na_2SO_4 and concentrated under reduced pressure. Products were then purified on silica. Seebach's stain turned out to be superior in visualizing the product on TLC plates.

Care must be taken regarding reduced pressure since the compounds are, in some cases, volatile. 20 mbar and 40 °C water bath worked fine in most cases, exception being the 2,4-difluoro (compound X).

[1]In cases where acid sensitive compounds were used only water was used and turned out to work OK.

Characterization data and preparation of starting materials

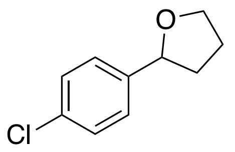

(1aa) 2-(4-chlorophenyl)tetrahydrofuran: Prepared according to *Method I*. Slight yellowish oil (595 mg, **66**% yield). **^{1}H-NMR** (400 MHz, $CDCl_3$) δ 7.37 – 7.18 (m, 4H), 4.86 (t, *J* = 7.2 Hz, 1H), 4.08 (dt, *J* = 8.3, 6.8 Hz, 1H), 3.93 (dt, *J* = 8.3, 6.9 Hz, 1H), 2.37 – 2.26 (m, 1H), 2.05 – 1.96 (m, 2H), 1.80 – 1.69 (m, 1H). **^{13}C-NMR** (201 MHz, $CDCl_3$) δ 142.2, 132.9, 128.5, 127.1, 80.1, 68.9, 34.8, 26.1. **MS-EI**(m/z): calculated for $C_{10}H_{11}ClO^+$, 182,0498; found 182.1. Spectroscopic data is in agreement with[2].

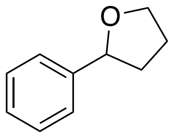

(2aa) 2-phenyltetrahydrofuran: Prepared according to *Method I* (2.28 mmol scale). Slight yellowish oil (219 mg, **53** % yield). **^{1}H-NMR** (400 MHz, $CDCl_3$) δ 7.35 – 7.31 (m, 4H), 7.28 – 7.22 (m, 2H), 4.89 (t, *J* = 7.2 Hz, 1H), 4.10 (ddd, *J* = 8.3, 7.1, 6.5 Hz, 1H), 3.94 (ddd, *J* = 8.2, 7.5, 6.4 Hz, 1H), 2.37 – 2.27 (m, 1H), 2.07 – 1.95 (m, 2H), 1.81 (ddt, *J* = 12.2, 8.6, 7.5 Hz, 1H). **^{13}C-NMR** (201 MHz, $CDCl_3$) δ 143.6, 128.4, 127.2, 125.8, 80.8, 68.8, 34.7, 26.2. **MS-EI**(m/z): calculated for $C_{10}H_{12}O^+$, 148,0888; found 148.1. Spectroscopic data is in agreement with[2].

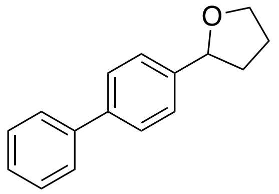

(2bb) 2-([1,1'-biphenyl]-4-yl)tetrahydrofuran: Prepared according *Method II*. Colorless oil (135 mg, **30**% yield), R_f (2% EtOAc in hexane) = 0.10. **^{1}H-NMR** (400 MHz, $CDCl_3$) δ 7.61 – 7.54 (m, 4H), 7.46 – 7.39 (m, 4H), 7.36 – 7.31 (m, 1H), 4.94 (t, *J* = 7.2 Hz, 1H), 4.13 (ddd, *J* = 8.3, 7.1, 6.4 Hz, 1H), 3.96 (ddd, *J* = 8.2, 7.6, 6.3 Hz, 1H), 2.41 – 2.31 (m, 1H), 2.12 – 1.96 (m, 2H), 1.86 (ddt, *J* = 12.2, 8.8, 7.6 Hz, 1H). **^{13}C-NMR** (101 MHz, $CDCl_3$) δ 142.6, 141.0, 140.1, 128.8, 127.2, 127.1, 127.1, 126.1, 80.5, 68.7, 34.6, 26.1. **MS-EI**(m/z): calculated for $C_{16}H_{16}O^+$, 224.1201; found 224.2. Spectroscopic data is in agreement with[3]

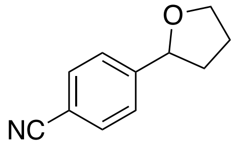

(2cc) 4-(tetrahydrofuran-2-yl)benzonitrile: Prepared according to *Method II*. Colorless oil (130 mg, **38**% yield), R_f (5% EtOAc in petroleum ether) = 0.08. **^{1}H-NMR** (400 MHz, $CDCl_3$) δ 7.62 (d, *J* = 8.4 Hz, 2H), 7.46 – 7.41 (m, 2H), 4.94 (t, *J* = 7.2 Hz, 1H), 4.10 (dtd, *J* = 8.3, 6.7, 0.5 Hz, 1H), 3.97 (ddd, *J* = 8.4, 7.3, 6.5 Hz, 1H), 2.43 – 2.33 (m, 1H), 2.08 – 1.95 (m, 2H), 1.74 (ddt, *J* = 12.2, 8.0, 7.6 Hz, 1H). **^{13}C-NMR** (201 MHz, $CDCl_3$) δ 149.4, 132.3, 126.3, 119.1, 110.9, 80.0, 69.1, 34.8, 26.1. **MS-EI**(m/z): calculated for $C_{11}H_{11}NO^+$, 173.0841; found 173.1. Spectroscopic data is in agreement with[4].

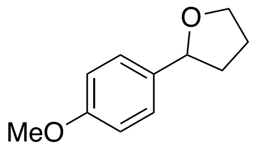

(2dd) 2-(4-methoxyphenyl)tetrahydrofuran: Prepared according to *Method I.* Slight yellowish oil (523 mg, **58** % yield). ^{1}**H-NMR** (400 MHz, $CDCl_3$) δ 7.29 – 7.23 (m, 2H), 6.89 – 6.84 (m, 2H), 4.83 (t, J = 7.2 Hz, 1H), 4.11 – 4.03 (m, 1H), 3.94 – 3.87 (m, 1H), 3.80 (s, 3H), 2.32 – 2.22 (m, 1H), 2.08 – 1.92 (m, 2H), 1.84 – 1.73 (m, 1H). ^{13}**C-NMR** (201 MHz, $CDCl_3$) δ 158.9, 135.5, 127.1, 113.8, 80.6, 68.6, 55.4, 34.6, 26.2. **MS-EI**(m/z): calculated for $C_{11}H_{14}O_2^+$, 178.0994; found 178.1. Spectroscopic data is in agreement with[2].

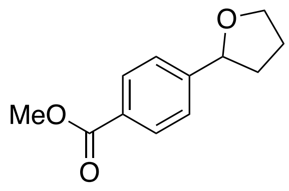

(2ee) methyl 4-(tetrahydrofuran-2-yl)benzoate: Prepared according to *Method II.* White solid (161 mg, **39**% yield), R_f (2% EtOAc in hexane) = 0.05. ^{1}**H-NMR** (400 MHz, $CDCl_3$) δ 8.00 (d, J = 8.3 Hz, 2H), 7.40 (d, J = 8.3 Hz, 2H), 4.95 (t, J = 7.2 Hz, 1H), 4.15 – 4.07 (m, 1H), 4.00 – 3.93 (m, 1H), 3.91 (s, 3H), 2.41 – 2.31 (m, 1H), 2.06 – 1.96 (m, 2H), 1.83 – 1.72 (m, 1H). ^{13}**C-NMR** (101 MHz, $CDCl_3$) δ 169.2, 151.3, 131.9, 131.2, 127.7, 82.4, 71.1, 54.3, 37.0, 28.2. **MS-EI**(m/z): calculated for $C_{12}H_{14}O_3^+$, 206.0943; found 206.1. Spectroscopic data is in agreement with[4].

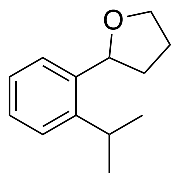

(2ff) 2-(2-isopropylphenyl)tetrahydrofuran: Prepared according to *Method II.* Colorless oil (139 mg, **37**% yield), R_f (2% EtOAc in pentane) = 0.12. ^{1}**H-NMR** (400 MHz, $CDCl_3$) δ 7.47 – 7.43 (m, 1H), 7.29 – 7.16 (m, 3H), 5.18 (t, J = 7.2 Hz, 1H), 4.16 (ddd, J = 8.3, 7.0, 6.1 Hz, 1H), 3.94 (dt, J = 8.2, 7.1 Hz, 1H), 3.16 (hept, J = 6.9 Hz, 1H), 2.42 – 2.28 (m, 1H), 2.10 – 1.93 (m, 2H), 1.72 (ddt, J = 12.2, 8.5, 7.4 Hz, 1H), 1.25 (d, J = 6.8 Hz, 6H). ^{13}**C-NMR** (201 MHz, $CDCl_3$) δ 145.4, 140.3, 127.3, 126.0, 125.2, 125.0, 77.5, 68.8, 34.7, 28.5, 26.3, 24.3, 24.0. **HRMS-ESI**(m/z): calculated for $C_{13}H_{19}O^+$ $[(M+H)^+]$ 191.1430; found 191.1429. **IR** (cm^{-1}): 1054 (C-O).

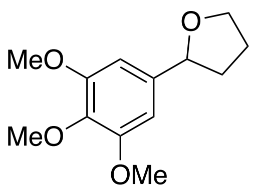

(2gg) 2-(3,4,5-trimethoxyphenyl)tetrahydrofuran: Prepared according to *Method II.* Colorless oil (179 mg, **38**% yield), R_f (15% EtOAc in hexane) = 0.23. ^{1}**H-NMR** (400 MHz, $CDCl_3$) δ 6.56 (d, J = 0.6 Hz, 2H), 4.82 (t, J = 7.2 Hz, 1H), 4.10 (ddd, J = 8.3, 7.2, 6.4 Hz, 1H), 3.96 – 3.89 (m, 1H), 3.87 (s, 6H), 3.83 (s, 3H), 2.36 – 2.25 (m, 1H), 2.10 – 1.93 (m, 2H), 1.80 (ddt, J = 12.1, 8.7, 7.7 Hz, 1H). ^{13}**C-NMR** (201 MHz, $CDCl_3$) δ 153.3, 139.2, 137.1, 102.6, 80.9, 68.8, 61.0, 56.2, 34.7, 26.1. **MS-EI**(m/z): calculated for $C_{13}H_{18}O_4^+$, 238.1205; found 238.2. Spectroscopic data is in agreement with[5].

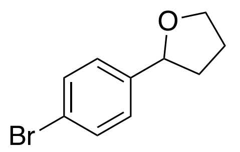

(2hh) 2-(4-bromophenyl)tetrahydrofuran: Prepared according to *Method II.* Colorless oil (118 mg, **26**% yield), R_f (2% EtOAc in petroleum ether) = 0.11. ^{1}**H-NMR** (400 MHz, $CDCl_3$) δ 7.45 (d, J = 8.7 Hz, 2H), 7.20 (d, J = 8.7 Hz, 2H), 4.84 (t, J = 7.2 Hz, 1H), 4.11 – 4.04 (m, 1H), 3.96 – 3.88 (m, 1H), 2.36 – 2.25 (m, 1H), 2.04 – 1.94 (m, 2H), 1.80 – 1.68 (m, 1H). ^{13}**C-NMR** (101 MHz, $CDCl_3$) δ 142.6, 131.3, 127.3, 120.8, 80.0, 68.7, 34.6, 25.9. **MS-EI**(m/z): calculated for $C_{10}H_{11}BrO^+$, 225.9993; found 226.1. Spectroscopic data is in agreement with[6].

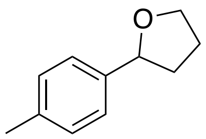

(2ii) 2-(*p*-tolyl)tetrahydrofuran: Prepared according to *Method II.* Colorless oil (102 mg, **31**% yield), R_f (2% EtOAc in pentane) = 0.09. **^{1}H-NMR** (400 MHz, $CDCl_3$) δ 7.23 (d, *J* = 8.0 Hz, 2H), 7.14 (d, *J* = 8.0 Hz 2H), 4.86 (t, *J* = 7.2 Hz, 1H), 4.09 (dt, *J* = 8.1, 6.9 Hz, 1H), 3.96 – 3.88 (m, 1H), 2.34 (s, 3H), 2.33 – 2.25 (m, 1H), 2.08 – 1.93 (m, 2H), 1.80 (ddt, *J* = 12.1, 8.6, 7.6 Hz, 1H). **^{13}C-NMR** (201 MHz, $CDCl_3$) δ 140.5, 136.8, 129.1, 125.8, 80.7, 68.7, 34.7, 26.2, 21.2. **MS-EI**(m/z): calculated for $C_{11}H_{14}O^+$, 162.1045; found 162.1. Spectroscopic data is in agreement with[5].

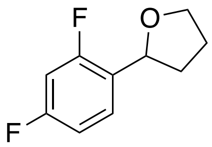

(2jj) 2-(2,4-difluorophenyl)tetrahydrofuran: Prepared according to *Method II.* Colorless liquid (26 mg, **10**% yield), R_f (2% Et_2O in petroleum ether) = 0.14. **^{1}H-NMR** (400 MHz, $CDCl_3$) δ 7.41 (tdd, *J* = 8.5, 6.6, 0.8 Hz, 1H), 6.88 – 6.81 (m, 1H), 6.76 (ddd, *J* = 10.6, 8.9, 2.5 Hz, 1H), 5.08 (t, *J* = 7.1 Hz, 1H), 4.08 (dt, *J* = 8.3, 6.7 Hz, 1H), 3.92 (dt, *J* = 8.3, 6.9 Hz, 1H), 2.38 (dqd, *J* = 12.2, 6.9, 1.2 Hz, 1H), 2.05 – 1.94 (m, 2H), 1.75 (dqd, *J* = 12.4, 7.5, 0.9 Hz, 1H). **^{13}C-NMR** (201 MHz, $CDCl_3$) δ 162.2 (dd, *J* = 247.1, 11.9 Hz), 159.9 (dd, *J* = 248.4, 11.8 Hz), 127.8 (dd, *J* = 9.6, 6.2 Hz), 126.8 (d, *J* = 13.7 Hz), 111.1 (dd, *J* = 21.1, 3.6 Hz), 103.7 (t, *J* = 25.5 Hz), 74.9, 68.8, 33.7, 26.1. **^{19}F-NMR** (470 MHz, $CDCl_3$) δ -112.75 (quint, *J* = 7.6 Hz, 1F), -114.87 (q, *J* = 8.5 Hz, 1F). **HRMS-ESI**(m/z): calculated for $C_{10}H_{11}F_2O^+$ [$(M+H)^+$] 185.0772; found 185.0755. **IR** (cm^{-1}): 1271 (C-F), 1062 (C-O).

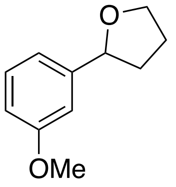

(2kk) 2-(3-methoxyphenyl)tetrahydrofuran: Prepared according to *Method II.* Colorless oil (131 mg, **37**% yield), R_f (5% EtOAc in petroleum ether) = 0.16. **^{1}H-NMR** (400 MHz, $CDCl_3$) δ 7.25 (t, *J* = 8.1 Hz, 1H), 6.92 - 6.89 (m, 2H), 6.79 (dddd, *J* = 8.2, 2.5, 1.1, 0.4 Hz, 1H), 4.88 (t, *J* = 7.1 Hz, 1H), 4.13 – 4.06 (m, 1H), 4.00 – 3.89 (m, 1H), 3.81 (s, 3H), 2.37 – 2.26 (m, 1H), 2.07 – 1.92 (m, 2H), 1.86 – 1.75 (m, 1H). **^{13}C-NMR** (201 MHz, $CDCl_3$) δ 159.8, 145.4, 129.4, 118.1, 112.7, 111.2, 80.6, 68.8, 55.3, 34.7, 26.1. **MS-EI**(m/z): calculated for $C_{11}H_{14}O_2^+$, 178.0994; found 178.1. Spectroscopic data is in agreement with[4].

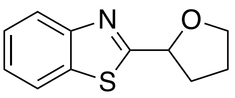

(2ll) 2-(tetrahydrofuran-2-yl)benzo[*d*]thiazole: Prepared according to previously reported procedure[7]. Slight yellowish oil (59 mg, **14**% yield), R_f (5% EtOAc in petroleum ether) = 0.07. **^{1}H-NMR** (400 MHz, $CDCl_3$) δ 7.98 (dt, *J* = 8.1, 1.0 Hz, 1H), 7.89 (ddd, *J* = 8.0, 1.3, 0.6 Hz, 1H), 7.47 (ddd, *J* = 8.3, 7.2, 1.3 Hz, 1H), 7.37 (ddd, *J* = 8.3, 7.2, 1.2 Hz, 1H), 5.35 (dd, *J* = 7.8, 5.4 Hz, 1H), 4.16 (dt, *J* = 8.4, 6.6 Hz, 1H), 4.01 (dt, *J* = 8.3, 7.0 Hz, 1H), 2.53 (dq, *J* = 12.6, 7.6 Hz, 1H), 2.34 – 2.23 (m, 1H), 2.10 – 1.99 (m, 2H). **^{13}C-NMR** (201 MHz, $CDCl_3$) δ 176.6, 153.7, 134.8, 126.1, 125.0, 122.9, 121.9, 78.9, 69.6, 33.5, 25.9. **MS-EI**(m/z): calculated for $C_{11}H_{11}NOS^+$, 205.0561; found 205.1. Spectroscopic data is in agreement with[7].

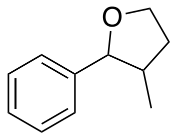

(3aa) 3-methyl-2-phenyltetrahydrofuran: Under dry and inert conditions, LDA (1.1 eq. 3.3 ml (2M)) and 30 ml anhydrous THF was cooled to -78 °C. Propiophenone (1 eq., 6 mmol, 0.8 ml) dissolved in 3 ml anhydrous THF was added slowly to the LDA solution and stirred for 1h at -78 °C. Methyl-2-bromoacetate (1.2 eq., 0.68 ml) dissolved in 3 ml anhydrous THF was slowly added to the reaction solution and then warmed to r.t and stirred overnight. Reaction was quenched with sat. NH_4Cl and extracted with EtOAc three times, dried over Na_2SO_4 and concentrated under reduced pressure. Crude mixture was purified on silica (4% EtOAc in petroleum ether) to give methyl 3-methyl-4-oxo-4-phenylbutanoate (690 mg, **56**% yield). **^{1}H-NMR** (400 MHz, $CDCl_3$) δ 8.01 – 7.96 (m, 2H), 7.60 – 7.54 (m, 1H), 7.51 – 7.44 (m, 2H), 3.95 (dqd, *J* = 8.4, 7.2, 5.8 Hz, 1H), 3.65 (s, 3H), 2.97 (dd, *J* = 16.8, 8.4 Hz, 1H), 2.47 (dd, *J* = 16.8, 5.8 Hz, 1H), 1.23 (d, *J* = 7.2 Hz, 3H). Methyl 3-methyl-4-oxo-4-phenylbutanoate was then transformed to **methyl-2-phenyltetrahydrofuran** by following *Method I*, yielding an inseparable mixture of two diastereoisomers. Colorless oil (132 mg, **25**% overall yield). **^{1}H-NMR** (400 MHz, $CDCl_3$) δ 7.35 – 7.29 (m, 2H), 7.26 – 7.20 (m, 3H), 4.96 (d, *J* = 6.5 Hz, 1H), 4.19 (dddd, *J* = 8.2, 7.6, 5.6, 0.5 Hz, 1H), 3.93 (td, *J* = 8.1, 6.8 Hz, 1H), 2.59 – 2.46 (m, 1H), 2.23 – 2.13 (m, 1H), 1.80 – 1.64 (m, 1H), 0.61 (d, *J* = 7.0 Hz, 3H). **^{13}C-NMR** (201 MHz, $CDCl_3$) δ 140.9, 128.0, 126.9, 126.4, 83.8, 67.4, 37.8, 33.9, 15.5. **MS-EI**(m/z): calculated for $C_{11}H_{14}O^+$, 162.1045; found 162.1. Spectroscopic data is in agreement with[8].

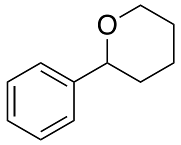

(3bb) 2-phenyltetrahydro-2*H*-pyran: Prepared according to *Method II*. Colorless oil (83 mg, **26**% yield), R_f (2% EtOAc in petroleum ether) = 0.17. **^{1}H-NMR** (400 MHz, $CDCl_3$) δ 7.37 – 7.30 (m, 4H), 7.28 – 7.23 (m, 1H), 4.32 (dd, *J* = 10.8, 2.3 Hz, 1H), 4.19 – 4.10 (m, 1H), 3.62 (td, *J* = 11.6, 2.5 Hz, 1H), 1.99 – 1.91 (m, 1H), 1.87 – 1.80 (m, 1H), 1.74 – 1.56 (m, 4H). **^{13}C-NMR** (201 MHz, $CDCl_3$) δ 143.5, 128.4, 127.4, 126.0, 80.3, 69.2, 34.2, 26.0, 24.2. **MS-EI**(m/z): calculated for $C_{11}H_{14}O^+$, 162.1045; found 162.2. Spectroscopic data is in agreement with[9].

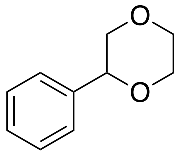

(3cc) 2-phenyl-1,4-dioxane: Prepared according to *Method II*. Colorless oil (171 mg, **52**% yield), R_f (2% EtOAc in petroleum ether) = 0.12. **^{1}H-NMR** (400 MHz, $CDCl_3$) δ 7.37 – 7.34 (m, 4H), 7.34 – 7.28 (m, 1H), 4.63 (dd, *J* = 10.2, 2.8 Hz, 1H), 3.99 – 3.70 (m, 5H), 3.48 (dd, *J* = 11.7, 10.2 Hz, 1H). **^{13}C-NMR** (201 MHz, $CDCl_3$) δ 138.4, 128.6, 128.2, 126.4, 78.1, 72.6, 67.2, 66.5. **MS-EI**(m/z): calculated for $C_{10}H_{12}O_2^+$, 164.0837; found 164.1. Spectroscopic data is in agreement with[2].

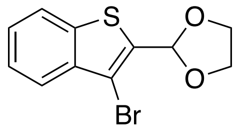

(3gg) 2-(3-bromobenzo[*b*]thiophen-2-yl)-1,3-dioxolane: 3-bromobenzo[*b*]thiophene-2-carbaldehyde (1.5 mmol, 362 mg), ethylene glycol (2 eq., 0.17 ml), *para*-toluenesulfonic acid (0.1 eq., 29 mg) and toluene (10 ml) were refluxed with a Dean-Stark trap overnight. Reaction was cooled to r.t and diluted with sat. $NaHCO_3$, extracted three times with EtOAc, dried over Na_2SO_4 and concentrated under reduced pressure. Crude mixture was purified on silica R_f (5% EtOAc in petroleum ether) = 0.15. Colorless oil (240 mg, **56**% yield). **^{1}H-NMR** (400 MHz, $CDCl_3$) δ 7.85 – 7.79 (m, 2H), 7.49 – 7.37 (m, 2H), 6.35 (s, 1H), 4.26 – 4.04 (m, 4H). **^{13}C-NMR** (201 MHz, $CDCl_3$) δ 138.3, 137.8, 137.2, 126.1, 125.2, 123.4, 122.9, 107.9, 100.0, 65.8. **MS-EI**(m/z): calculated for $C_{11}H_9BrO_2S^+$, 283.9507; found 284.0. Spectroscopic data is in agreement with[10].

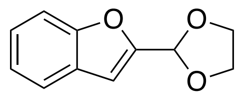

(3hh) 2-(1,3-dioxolan-2-yl)benzofuran: benzofuran-2-carbaldehyde (2 mmol, 293 mg), ethylene glycol (2 eq. 0.23 ml), *para*-toluenesulfonic acid (0.1 eq., 38 mg) and toluene (15 ml) were refluxed with a Dean-Stark trap overnight. Reaction was cooled to r.t and diluted with sat. $NaHCO_3$, extracted three times with EtOAc, dried over Na_2SO_4 and concentrated under reduced pressure. Crude mixture was purified on silica R_f (3% EtOAc in petroleum ether) = 0.09. Yellow oil (185 mg, **49**% yield). **^{1}H-NMR** (400 MHz, $CDCl_3$) δ 7.57 (ddd, J = 7.6, 1.4, 0.7 Hz, 1H), 7.50 (dq, J = 8.2, 0.9 Hz, 1H), 7.30 (ddd, J = 8.2, 7.2, 1.4 Hz, 1H), 7.25 – 7.20 (m, 1H), 6.83 – 6.79 (m, 1H), 6.10 (s, 1H), 4.24 – 4.04 (m, 4H). **^{13}C-NMR** (201 MHz, $CDCl_3$) δ 155.4, 153.6, 127.6, 125.0, 123.1, 121.6, 111.8, 105.4, 98.2, 65.5. **MS-EI**(m/z): calculated for $C_{11}H_{10}O_3^+$, 190.0630; found 190.1. Spectroscopic data is in agreement with[11].

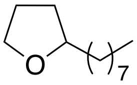

(3ii) 2-octyltetrahydrofuran: Prepared at 2x1 mmol scale according to the procedure in *Adv. Synth. Catal.* **2020**, 362, 2367-2372. Purified on silica R_f (1% EtOAc in hexane) = 0.19, colorless oil (215 mg, **58**% yield). **^{1}H-NMR** (400 MHz, $CDCl_3$) δ 3.91 – 3.82 (m, 1H), 3.81-3.74 (m, 1H), 3.74-3.67 (m, 1H), 2.02 – 1.77 (m, 3H), 1.47 – 1.35 (m, 3H), 1.35 – 1.22 (m, 12H), 0.92 – 0.85 (m, 3H). **^{13}C-NMR** (126 MHz, $CDCl_3$) δ 79.4, 67.6, 35.8, 31.9, 31.4, 29.8, 29.6, 29.3, 26.4, 25.7, 22.6, 14.1. **MS-EI**(m/z): calculated for $C_{12}H_{24}O^+$, 184.1822; found 184.2 (only weekly ionizing).

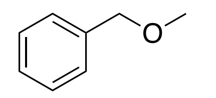

(3jj(a)) Benzylmethyl ether: To a solution of benzaldehyde (2 mmol, 212 mg) in 20 ml MeOH was added $NaBH_4$ (1.2 eq. 91 mg). Reaction was stirred until completed as judged by TLC. Reaction mixture was concentrated under reduced pressure and redissolved in Et_2O and washed with H_2O, dried over Na_2SO_4 and concentrated under reduced pressure. Without further purification, the benzyl alcohol was dissolved in 20 ml anhydrous THF and NaH (1.5 eq. 60% dispersion in paraffin liquid, 120 mg) was added portionwise and stirred for 10 minutes. Then methyl iodide (5 eq., 0.62 ml) was added in one portion and stirred overnight. The reaction mixture was adsorbed onto Celite and purified on silica R_f(5% EtOAc in hexane) = 0.28, colorless oil (110 mg, **45**% yield over two steps). **^{1}H-NMR** (400 MHz, $CDCl_3$) δ 7.39 – 7.26 (m, 5H), 4.46 (s, 2H), 3.39 (s, 3H). **MS-EI**(m/z): calculated for $C_8H_{10}O^+$, 122.0726; found 122.1.

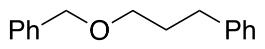

(3jj(b)) (3-(benzyloxy)propyl)benzene: To a solution of 3-phenyl-1-propanol (2 mmol, 272 mg) in 20 ml anhydrous THF and NaH (1.5 eq. 60% dispersion in paraffin liquid, 120 mg) was added portionwise and stirred for 10 minutes. Then benzyl bromide (2 eq. 0.50 ml) was added in one portion and stirred overnight. Reaction mixture was adsorbed onto Celite and purified on silica R_f(2% EtOAc in hexane) = 0.25, colorless oil (334 mg, **74**% yield). **^{1}H-NMR** (400 MHz, $CDCl_3$) δ 7.40 – 7.26 (m, 7H), 7.22 – 7.15 (m, 3H), 4.52 (s, 2H), 3.50 (t, *J* = 6.4 Hz, 2H), 2.77 – 2.68 (t, *J* = 7.5 Hz, 2H), 2.02 – 1.89 (m, 2H). **^{13}C-NMR** (126 MHz, $CDCl_3$) δ 142.1, 138.7, 128.6, 128.4, 128.4, 127.7, 127.6, 125.8, 73.0, 69.6, 32.5, 31.4. **MS-EI**(m/z): calculated for $C_{16}H_{18}O^+$, 226.1352; found 226.2.

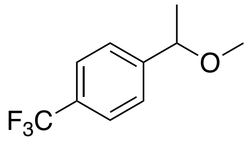

(3kk) 1-(1-methoxyethyl)-4-(trifluoromethyl)benzene: To a solution of 4′-(trifluoromethyl)acetophenone (2 mmol, 376 mg) in 20 ml MeOH was added $NaBH_4$ (1.2 eq. 91 mg). Reaction was stirred until completed as judged by TLC. Reaction mixture was concentrated under reduced pressure and redissolved in Et_2O and washed with H_2O, dried over Na_2SO_4 and concentrated under reduced pressure. Without further purification, the 2-(4-(trifluoromethyl)phenyl)-2-propanol was dissolved in 20 ml anhydrous THF and NaH (1.5 eq. 60% dispersion in paraffin liquid, 120 mg) was added portionwise and stirred for 10 minutes. Then methyl iodide (5 eq., 0.62 ml) was added in one portion and stirred overnight. The reaction mixture was adsorbed onto Celite and purified on silica R_f(10% EtOAc in hexane) = 0.40, colorless oil (145 mg, **36**% yield over two steps). **^{1}H-NMR** (400 MHz, $CDCl_3$) δ 7.61 (d, *J* = 8.1 Hz, 2H), 7.42 (d, *J* = 8.0 Hz, 2H), 4.35 (q, *J* = 6.5 Hz, 1H), 3.24 (s, 3H), 1.43 (d, *J* = 6.5, 3H). **^{13}C-NMR** (126 MHz, $CDCl_3$) δ 147.8, 147.8, 147.8, 147.8, 130.1, 129.8, 129.6, 129.3, 126.4, 125.5, 125.4, 125.4, 125.4, 123.1, 121.0, 79.1, 56.6, 23.8. **MS-EI**(m/z): calculated for $C_{10}H_{11}F_3O^+$, 204.0757; found 204.1.

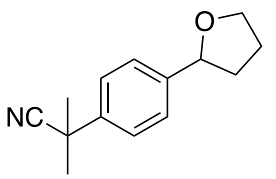

(4aa) 2-methyl-2-(4-(tetrahydrofuran-2-yl)phenyl)propane nitrile: Prepared at 0.4 mmol scale according to the procedure in *J. Org. Chem.* 2011, 76, 7, 2187–2194. Purified on silica R_f (10% EtOAc in petroleum ether) = 0.11, colorless oil (59 mg, **68**% yield). ^{1}**H-NMR** (400 MHz, $CDCl_3$) δ 7.45 – 7.41 (m, 2H), 7.37 – 7.33 (m, 2H), 4.89 (t, *J* = 7.2 Hz, 1H), 4.09 (ddd, *J* = 8.3, 7.1, 6.5 Hz, 1H), 3.98 – 3.90 (m, 1H), 2.38 – 2.27 (m, 1H), 2.06 – 1.96 (m, 2H), 1.84 – 1.74 (m, 1H), 1.71 (s, 6H). ^{13}**C-NMR** (201 MHz, $CDCl_3$) δ 143.3, 140.4, 126.3, 125.2, 124.7, 80.3, 68.8, 37.1, 34.7, 29.3, 29.3, 26.2. **HRMS-ESI**(m/z): calculated for $C_{14}H_{18}NO^+$ [$(M+H)^+$] and $C_{13}H_{17}O^+$ [$(M-HCN+H)^+$] 189.1279; found 189.1282. **IR** (cm^{-1}): 2235 (Nitrile), 1060 (C-O).

Experimental procedure for photoreaction

All reactions were run in duplicate unless otherwise stated. Dichloroethane (DCE, anhydrous) and $BrCCl_3$ were deoxygenated by sparging with argon for 20 min. Cyclic ether or acetal (0.2 mmol), $Fe(acac)_3$ (1 mol%) and a stir bar was added to a Biotage MW vial (10 ml) and capped. Reaction atmosphere was exchanged to argon by "sparging" head space for 10 min. Then $BrCCl_3$ (3 eq. 60 µL) followed by DCE (0.1 M, 2 ml) was added. The capped end of the vial was wrapped with parafilm and then placed in a photoreactor and irradiated with 455 nm light for 18 h at 27 °C (with fan). The two duplicated reactions were combined and adsorbed onto Celite and purified on silica. Isolated yields were thereby obtained as an average.

Compounds on TLC-plates were visualized with UV (254 nm), Seebach's stain or $KMnO_4$.

Characterization data for photoproducts

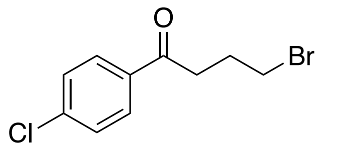

(1a) 4-bromo-1-(4-chlorophenyl)butan-1-one: Colorless oil (93 mg, **90**% isolated yield), R_f (2% EtOAc in hexane) = 0.12. **^{1}H-NMR** (400 MHz, $CDCl_3$) δ 7.92 (d, *J* = 8.6 Hz, 2H), 7.44 (d, *J* = 8.6 Hz, 2H), 3.54 (t, *J* = 6.3 Hz, 2H), 3.15 (t, *J* = 6.9 Hz, 2H), 2.30 (appar. quint, *J* = 6.5 Hz, 2H). **^{13}C-NMR** (201 MHz, $CDCl_3$) δ 197.7, 139.9, 135.2, 129.6, 129.1, 36.7, 33.6, 26.9. **MS-ESI**(m/z): calculated for $C_{10}H_{11}BrClO^+$ [$(M+H)^+$] 260.9682; found 260.1. Spectroscopic data is in agreement with[12].

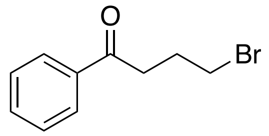

(2a) 4-bromo-1-phenylbutan-1-one: Yellow/greenish oil (71 mg, **78**% isolated yield), R_f (2% EtOAc in petroleum ether) = 0.14. **^{1}H-NMR** (400 MHz, $CDCl_3$) δ 8.01 – 7.96 (m, 2H), 7.61 – 7.55 (m, 1H), 7.51 – 7.45 (m, 2H), 3.56 (t, *J* = 6.3 Hz, 2H), 3.19 (t, *J* = 6.9 Hz, 2H), 2.32 (tt, *J* = 6.9, 6.3 Hz, 2H). **^{13}C-NMR** (201 MHz, $CDCl_3$) δ 199.0, 136.9, 133.4, 128.8, 128.2, 36.7, 33.8, 27.0. **MS-ESI**(m/z): calculated for $C_{10}H_{12}BrO^+$ [$(M+H)^+$] 227.0072; found 227.1. Spectroscopic data is in agreement with[12].

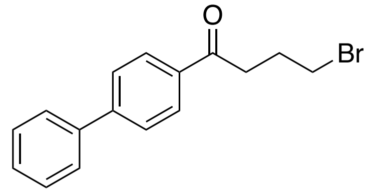

(2b) 1-([1,1'-biphenyl]-4-yl)-4-bromobutan-1-one: Off-white solids (94 mg, **78**% isolated yield), R_f(2% EtOAc in hexane) = 0.14. **^{1}H-NMR** (400 MHz, $CDCl_3$) δ 8.08 – 8.03 (m, 2H), 7.72 – 7.67 (m, 2H), 7.66 – 7.60 (m, 2H), 7.51 – 7.45 (m, 2H), 7.44 – 7.38 (m, 1H), 3.58 (t, *J* = 6.3 Hz, 2H), 3.23 (t, *J* = 6.9 Hz, 2H), 2.34 (appar. quint, , *J* = 6.6 Hz, 2H). **^{13}C-NMR** (201 MHz, $CDCl_3$) δ 198.6, 146.1, 140.0, 135.6, 129.1, 128.8, 128.4, 127.5, 127.4, 36.8, 33.8, 27.1. **MS-ESI**(m/z): calculated for $C_{16}H_{16}BrO^+$ [$(M+H)^+$] 303.0385; found 303.0. Spectroscopic data is in agreement with[12].

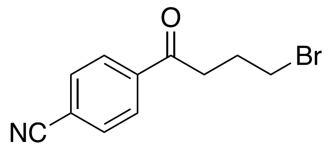

(2c) 4-(4-bromobutanoyl)benzonitrile: Off-white solids (82 mg, **81**% isolated yield), R_f (5% EtOAc in petroleum ether) = 0.07. **^{1}H-NMR** (400 MHz, $CDCl_3$) δ 8.07 (d, , *J* = 8.7 Hz, 2H), 7.79 (d, *J* = 8.7 Hz 2H), 3.56 (t, *J* = 6.2 Hz, 2H), 3.21 (t, *J* = 6.9 Hz, 2H), 2.33 (appar. quint, *J* = 6.6 Hz, 2H). **^{13}C-NMR** (201 MHz, $CDCl_3$) δ 197.6, 139.7, 132.7, 128.6, 118.0, 116.7, 37.0, 33.4, 26.6. **HRMS-ESI**(m/z): calculated for $C_{11}H_{11}BrNO^+$ [$(M+H)^+$] 252.0019; found 252.0000. **IR** (cm^{-1}): 2228 (Nitrile), 1691(C=O). **mp**: 76.0-77.1 ºC.

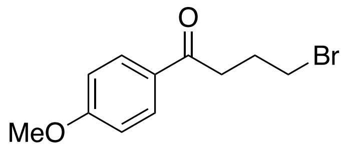

(2d) 4-bromo-1-(4-methoxyphenyl)butan-1-one: Pinkish oil (82 mg, **79**% isolated yield), R_f (3% EtOAc in petroleum ether) = 0.09. **^{1}H-NMR** (400 MHz, $CDCl_3$) δ 7.97 (d, *J* = 9.0 Hz, 2H), 6.95 (d, *J* = 9.0 Hz, 2H), 3.88 (s, 3H), 3.55 (t, *J* = 6.3 Hz, 2H), 3.13 (t, *J* = 6.9 Hz, 2H), 2.31 (appar. quint, *J* = 6.7 Hz, 2H). **^{13}C-NMR** (201 MHz, $CDCl_3$) δ 197.5, 163.7, 130.4, 130.0, 113.9, 55.6, 36.3, 33.9, 27.2. **MS-ESI**(m/z): calculated for $C_{11}H_{14}BrO_2^+$ [$(M+H)^+$] 257.0177; found 257.0. Spectroscopic data is in agreement with[12].

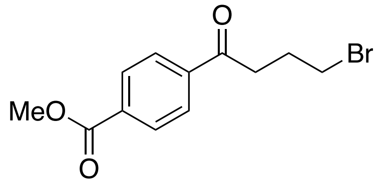

(2e) methyl 4-(4-bromobutanoyl)benzoate: White solids (94 mg, **82**% isolated yield), R_f (5% EtOAc in hexane) = 0.07. **^{1}H-NMR** (400 MHz, $CDCl_3$) δ 8.13 (d, *J* = 8.5 Hz, 2H), 8.03 (d, *J* = 8.5 Hz, 2H), 3.96 (s, 3H), 3.56 (t, *J* = 6.3 Hz, 2H), 3.22 (t, *J* = 6.9 Hz, 2H), 2.33 (appar. quint, *J* = 6.6 Hz, 2H). **^{13}C-NMR** (201 MHz, $CDCl_3$) δ 198.5, 166.3, 140.0, 134.2, 130.1, 128.1, 52.6, 37.1, 33.6, 26.8. **HRMS** (m/z): calculated for $C_{12}H_{14}BrO_3^+$ [(M+H)$^+$] 285.0126; not detected using either EI, ESI or APCI. **IR** (cm^{-1}): 1715 (C=O), 1687 (C=O ester). **mp**: 66.7-77.9 °C.

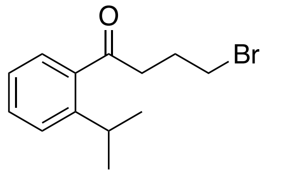

(2f) 4-bromo-1-(2-isopropylphenyl)butan-1-one: Yellow oil (99 mg, **92**% isolated yield), R_f (2% EtOAc in pentane) = 0.10. **^{1}H-NMR** (400 MHz, $CDCl_3$) δ 7.46 – 7.39 (m, 3H), 7.25 – 7.21 (m, 1H), 3.55 (t, *J* = 6.4 Hz, 2H), 3.31 (hept, *J* = 6.8 Hz, 1H), 3.07 (t, *J* = 6.9 Hz, 2H), 2.29 (appar. quint, *J* = 6.5 Hz, 2H), 1.24 (d, *J* = 6.8, 6H). **^{13}C-NMR** (201 MHz, $CDCl_3$) δ 205.1, 147.5, 139.0, 131.1, 127.1, 126.7, 125.6, 40.9, 33.5, 29.7, 27.0, 24.3. **HRMS-ESI**(m/z): calculated for $C_{13}H_{18}BrO^+$ [(M+H)$^+$] 269.0536; found 269.0513. **IR** (cm^{-1}): 1686 (C=O).

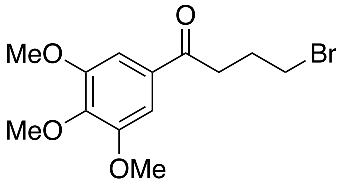

(2g) 4-bromo-1-(3,4,5-trimethoxyphenyl)butan-1-one: Off-white solids (109 mg, **86**% isolated yield), R_f (10% EtOAc in petroleum ether) = 0.10. **^{1}H-NMR** (400 MHz, $CDCl_3$) δ 7.24 (s, 2H), 3.93 (s, 6H), 3.92 (s, 3H), 3.56 (t, *J* = 6.2 Hz, 2H), 3.15 (t, *J* = 6.9 Hz, 2H), 2.32 (appar. quint, *J* = 6.5 Hz, 2H). **^{13}C-NMR** (201 MHz, $CDCl_3$) δ 197.8, 153.2, 142.9, 132.1, 105.7, 61.1, 56.5, 36.4, 33.8, 27.3. **HRMS-ESI**(m/z): calculated for $C_{13}H_{18}BrO_4^+$ [(M+H)$^+$] 317.0388; found 317.0393. **IR** (cm^{-1}): 1671 (C=O), 1231 (C-O). **mp**: 87.3-88.5 °C.

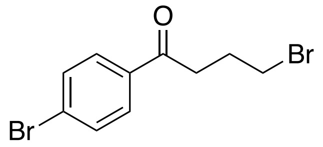

(2h) 4-bromo-1-(4-bromophenyl)butan-1-one: Colorless oil (101 mg, **83**% isolated yield), R_f (2% EtOAc in petroleum ether) = 0.10. **^{1}H-NMR** (400 MHz, $CDCl_3$) δ 7.84 (d, *J* = 8.7 Hz, 2H), 7.61 (d, *J* = 8.7 Hz, 2H), 3.54 (t, *J* = 6.3 Hz, 2H), 3.15 (t, *J* = 6.9 Hz, 2H), 2.30 (appar. quint, *J* = 6.6 Hz, 2H). **^{13}C-NMR** (201 MHz, $CDCl_3$) δ 197.9, 135.6, 132.1, 129.7, 128.6, 36.7, 33.6, 26.8. **MS-ESI**(m/z): calculated for $C_{10}H_{11}Br_2O^+$ [(M+H)$^+$] 304.9177; found 305.0. Spectroscopic data is in agreement with[12].

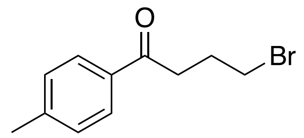

(2i) 4-bromo-1-(*p*-tolyl)butan-1-one: Colorless oil (75 mg, **78**% isolated yield), R_f (2% EtOAc in pentane) = 0.10. **^{1}H-NMR** (400 MHz, $CDCl_3$) δ 7.87 (d, *J* = 8.2 Hz, 2H), 7.28 – 7.23 (m, 2H), 3.54 (t, *J* = 6.4 Hz, 2H), 3.15 (t, *J* = 7.0 Hz, 2H), 2.41 (s, 3H), 2.30 (tt, *J* = 6.9, 6.3 Hz, 2H). **^{13}C-NMR** (201 MHz, $CDCl_3$) δ 198.6, 144.2, 134.4, 129.5, 128.3, 36.6, 33.8, 27.1, 21.8. **MS-ESI**(m/z): calculated for $C_{11}H_{14}BrO^+$ [(M+H)$^+$] 241.0228; found 241.0. Spectroscopic data is in agreement with[12].

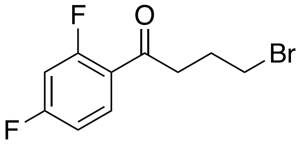

(2j) 4-bromo-1-(2,4-difluorophenyl)butan-1-one: Colorless liquid (34 mg, **71**% isolated yield), R_f(2% EtOAc in hexane) = 0.12. **^{1}H-NMR** (400 MHz, $CDCl_3$) δ 7.95 (td, *J* = 8.6, 6.6 Hz, 1H), 7.01 – 6.93 (m, 1H), 6.89 (ddd, *J* = 11.1, 8.6, 2.4 Hz, 1H), 3.53 (t, *J* = 6.4 Hz, 2H), 3.16 (td, *J* = 6.9, 3.3 Hz, 2H), 2.30 (appar. quint, *J* = 6.6 Hz, 2H). **^{13}C-NMR** (201 MHz, $CDCl_3$) δ 195.6 (d, *J* = 4.8 Hz), 166.0 (dd, *J* = 257.4, 12.3 Hz), 163.0 (dd, *J* = 257.7, 12.5 Hz), 132.8 (dd, *J* = 10.6, 4.3 Hz), 122.0 (d, *J* = 13.7 Hz), 112.4 (d, *J* = 21.4 Hz), 105.0 (t, *J* = 26.6 Hz), 41.6 (d, *J* = 7.8 Hz), 33.4, 26.8. **^{19}F-NMR** (470 MHz, $CDCl_3$) δ -101.52 – -101.73 (m, 1F), -104.09 (tdd, *J* = 11.7, 7.2, 3.3 Hz, 1F). **HRMS-ESI**(m/z): calculated for $C_{10}H_{10}BrF_2O^+$ $[(M+H)^+]$ 262.9883; found 262.9881. **IR** (cm^{-1}): 1685 (C=O).

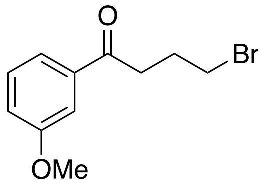

(2k) 4-bromo-1-(3-methoxyphenyl)butan-1-one: Colorless oil (83 mg, **82**% isolated yield), R_f(2% EtOAc in petroleum ether) = 0.10. **^{1}H-NMR** (400 MHz, $CDCl_3$) δ 7.56 (ddd, *J* = 7.7, 1.6, 0.9 Hz, 1H), 7.49 (dd, *J* = 2.7, 1.6 Hz, 1H), 7.38 (t, *J* = 7.9 Hz, 1H), 7.11 (ddd, *J* = 8.2, 2.7, 1.0 Hz, 1H), 3.86 (s, 3H), 3.54 (t, *J* = 6.4 Hz, 2H), 3.17 (t, *J* = 6.9 Hz, 2H), 2.30 (appar. quint, *J* = 6.7 Hz, 2H). **^{13}C-NMR** (201 MHz, $CDCl_3$) δ 198.8, 160.0, 138.2, 129.8, 120.8, 119.8, 112.4, 55.6, 36.8, 33.7, 27.1. **MS-ESI**(m/z): calculated for $C_{11}H_{14}BrO_2^+$ $[(M+H)^+]$ 257.0177; found 257.0. Spectroscopic data is in agreement with[13].

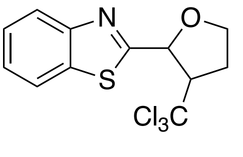

(2l) 2-(3-(trichloromethyl)tetrahydrofuran-2-yl)benzo[*d*]thiazole: Colorless oil (27 mg, **21**% isolated yield), R_f(5% EtOAc in petroleum ether) = 0.33. **^{1}H-NMR** (400 MHz, $CDCl_3$) δ 8.05 (d, *J* = 8.2 Hz, 1H), 7.90 (d, *J* = 8.0 Hz, 1H), 7.49 (ddd, *J* = 8.2, 7.2, 1.3 Hz, 1H), 7.40 (ddd, *J* = 8.2, 7.2, 1.0 Hz, 1H), 5.54 (d, *J* = 3.6 Hz, 1H), 4.40 (ddd, *J* = 9.3, 5.9, 3.6 Hz, 1H), 4.29 (td, *J* = 8.4, 4.5 Hz, 1H), 4.07 (appar. q, *J* = 8.2 Hz, 1H), 2.55 – 2.34 (m, 2H). **^{13}C-NMR** (201 MHz, $CDCl_3$) δ 172.0, 153.3, 135.8, 126.3, 125.6, 123.6, 122.0, 101.5, 81.1, 69.2, 66.0, 31.3. **HRMS-ESI**(m/z): calculated for $C_{12}H_{11}Cl_3NOS^+$ $[(M+H)^+]$ 321.9621; found 321.9627. **IR** (cm^{-1}): 1129 (C-O), 762 (C-Cl).

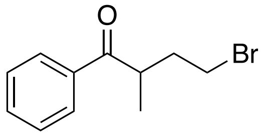

(3a) 4-bromo-2-methyl-1-phenylbutan-1-one: Greenish oil (81 mg, **95**% isolated yield), R_f (2% EtOAc in petroleum ether) = 0.11. **^{1}H-NMR** (400 MHz, $CDCl_3$) δ 8.02 – 7.98 (m, 2H), 7.61 – 7.56 (m, 1H), 7.52 – 7.46 (m, 2H), 3.81 (appar. sext, *J* = 6.4 Hz, 1H), 3.51 (ddd, *J* = 10.2, 7.0, 5.8 Hz, 1H), 3.43 (ddd, *J* = 10.2, 7.5, 5.7 Hz, 1H), 2.44 (dtd, *J* = 14.3, 7.1, 5.7 Hz, 1H), 1.96 (ddt, *J* = 14.5, 7.5, 6.0 Hz, 1H), 1.23 (d, *J* = 7.0 Hz, 3H). **^{13}C-NMR** (201 MHz, $CDCl_3$) δ 203.3, 136.3, 133.4, 128.9, 128.6, 39.0, 35.8, 32.2, 17.4. **IR** (cm^{-1}): 1678 (C=O). **HRMS-ESI**(m/z): calculated for $C_{11}H_{14}BrO^+$ $[(M+H)^+]$ 241.0228; found 241.0225.

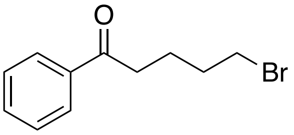

(3b) 5-bromo-1-phenylpentan-1-one: Off-white solids (80 mg, **84**% isolated yield), R_f (2% EtOAc in petroleum ether) = 0.12. **^{1}H-NMR** (400 MHz, $CDCl_3$) δ 7.98 – 7.93 (m, 2H), 7.60 – 7.54 (m, 1H), 7.50 – 7.44 (m, 2H), 3.46 (t, J = 6.4 Hz, 2H), 3.02 (t, J = 6.9 Hz, 2H), 2.02 – 1.85 (m, 4H). **^{13}C-NMR** (201 MHz, $CDCl_3$) δ 199.7, 137.0, 133.2, 128.8, 128.1, 37.5, 33.5, 32.3, 22.9. **MS-ESI**(m/z): calculated for $C_{11}H_{14}BrO^+$ $[(M+H)^+]$ 241.0228; found 241.0. Spectroscopic data is in agreement with[12].

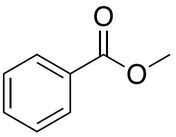

(3d) methyl benzoate: Slight yellow liquid (39 mg, **71**% isolated yield), R_f (2% EtOAc in pentane) = 0.19. **^{1}H-NMR** (400 MHz, $CDCl_3$) δ 8.06 – 8.01 (m, 2H), 7.58 – 7.53 (m, 1H), 7.47 – 7.41 (m, 2H), 3.92 (s, 3H). **^{13}C-NMR** (201 MHz, $CDCl_3$) δ 167.3, 133.0, 130.3, 129.7, 128.5, 52.2. **MS-EI**(m/z): calculated for $C_8H_8O_2^+$, 136.0524; found 136.1. Spectroscopic data is in agreement with[14].

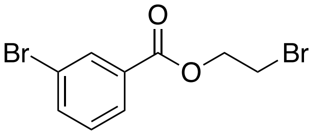

(3e) 2-bromoethyl 3-bromobenzoate: (purified prior to reaction). Colorless oil (107 mg, **86**% isolated yield and 252 mg, 82% isolated yield for the 1 mmol scale reaction), R_f (3% EtOAc in petroleum ether) = 0.16. **^{1}H-NMR** (400 MHz, $CDCl_3$) δ 8.20 (ddd, J = 2.0, 1.6, 0.4 Hz, 1H), 8.00 (ddd, J = 7.8, 1.6, 1.1 Hz, 1H), 7.71 (ddd, J = 8.0, 2.1, 1.1 Hz, 1H), 7.34 (td, J = 7.9, 0.4 Hz, 1H), 4.64 (t, J = 6.1 Hz, 2H), 3.64 (t, J = 6.1 Hz, 2H). **^{13}C-NMR** (201 MHz, $CDCl_3$) δ 164.9, 136.4, 132.9, 131.7, 130.2, 128.5, 122.7, 64.7, 28.7. **HRMS** (m/z): calculated for $C_9H_9Br_2O^+$ $[(M+H)^+]$ 306.8969; not detected using either EI, ESI or APCI. **IR** (cm^{-1}): 1721 (C=O).

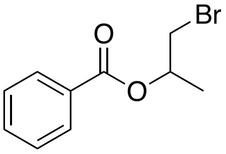

(3f) 1-bromopropan-2-yl benzoate: Colorless oil (92 mg, **95**% isolated yield), R_f (3% EtOAc in petroleum ether) = 0.16. **^{1}H-NMR** (400 MHz, $CDCl_3$) δ 8.09 – 8.04 (m, 2H), 7.60 – 7.54 (m, 1H), 7.49 – 7.42 (m, 2H), 5.33 (qt, J = 6.4, 5.1 Hz, 1H), 3.59 (dd, J = 5.1, 1.9 Hz, 2H), 1.49 (d, J = 6.4 Hz, 3H). **^{13}C-NMR** (201 MHz, $CDCl_3$) δ 165.9, 133.3, 130.2, 129.9, 128.5, 69.9, 35.6, 18.9. **HRMS** (m/z): calculated for $C_{10}H_{12}BrO_2^+$ $[(M+H)^+]$ 243.0021; not detected using either EI, ESI or APCI. **IR** (cm^{-1}): 1715 (C=O). Spectroscopic data is in agreement with.[15]

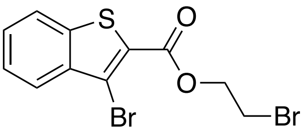

(3g) 2-bromoethyl 3-bromobenzo[*b*]thiophene-2-carboxylate: White solids (115 mg, **78** % isolated yield), R_f (2% EtOAc in petroleum ether) = 0.09. **^{1}H-NMR** (400 MHz, $CDCl_3$) δ 8.02 – 7.98 (m, 1H), 7.86 – 7.82 (m, 1H), 7.58 – 7.49 (m, 2H), 4.68 (t, J = 6.2, 2H), 3.67 (t, J = 6.2, 2H). **^{13}C-NMR** (201 MHz, $CDCl_3$) δ 161.0, 139.7, 138.8, 128.5, 126.8, 125.9, 125.6, 122.8, 116.0, 64.9, 28.3. **HRMS-ESI**(m/z): calculated for $C_{11}H_9Br_2O_2S^+$ $[(M+H)^+]$ 362.8685; found 362.8690. **IR** (cm^{-1}): 1716 (C=O). **mp**: 93.0-94.2 °C.

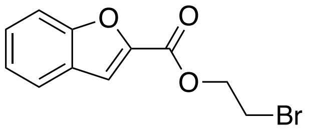

(3h) 2-bromoethyl benzofuran-2-carboxylate: Colorless oil (91 mg, **85** % isolated yield), R_f (6% EtOAc in petroleum ether) = 0.11. **^{1}H-NMR** (400 MHz, $CDCl_3$) δ 7.69 (ddd, *J* = 7.9, 1.4, 0.7 Hz, 1H), 7.62 – 7.57 (m, 2H), 7.46 (ddd, *J* = 8.6, 7.0, 1.2 Hz, 1H), 7.31 (td, *J* = 7.5, 0.9 Hz, 1H), 4.68 (t, *J* = 6.2 Hz, 2H), 3.66 (t, *J* = 6.2 Hz, 2H). **^{13}C-NMR** (201 MHz, $CDCl_3$) δ 159.1, 156.0, 144.9, 128.0, 127.0, 124.0, 123.1, 114.8, 112.5, 64.6, 28.4. **HRMS-ESI**(m/z): calculated for $C_{11}H_{10}BrO_3^+$ $[(M+H)^+]$ 268.9808; found 268.9799. **IR** (cm^{-1}): 1720 (C=O).

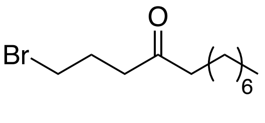

(3i) 1-bromododecan-4-one: Colorless oil isolated as an inseparable mixture of product and starting material with a conversion of **48**% as determined from ^{1}H-NMR (See Appendix). R_f(0.5% EtOAc in hexane) = 0.07. **MS-EI**(m/z): calculated for $C_{12}H_{23}BrO^+$, 262.0927; found 262.1 (only weekly ionizing).

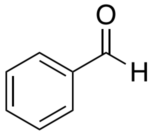

(3j) Benzaldehyde: Slight yellow oil (36 mg, **85**% yield (from 3jj(a)) and 32 mg, **81**% yield (from 3jj(b)), R_f(2% EtOAc in hexane) = 0.18. Care needs to be taken when evaporating the solvents as benzaldehyde seems abnormally volatile in these cases. **^{1}H-NMR** (400 MHz, $CDCl_3$) δ 10.05 (s, 1H), 7.89 (d, *J* = 7.3 Hz, 2H), 7.65 (t, *J* = 7.4 Hz, 1H), 7.55 (t, *J* = 7.5 Hz, 2H).

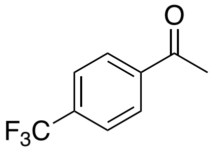

(3k) 4'-(trifluoromethyl)acetophenone: Colorless oil (60 mg, **80**% yield), R_f(2% EtOAc in hexane) = 0.17. Care needs to be taken when evaporating the solvents in this case to as the product seems somewhat volatile. **^{1}H-NMR** (400 MHz, $CDCl_3$) δ 8.06 (d, *J* = 8.0 Hz, 2H), 7.74 (d, *J* = 8.2 Hz, 2H), 2.65 (s, 3H).

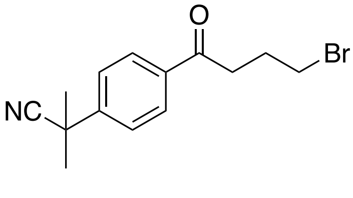

(4a) 2-(4-(4-bromobutanoyl)phenyl)-2-methylpropanenitrile: Conducted once at 0.1 mmol scale. White solids (24 mg, **82** % isolated yield), R_f (10% EtOAc in petroleum ether) = 0.09. **^{1}H-NMR** (400 MHz, $CDCl_3$) δ 8.00 (d, *J* = 8.6 Hz, 2H), 7.59 (d, *J* = 8.6 Hz, 2H), 3.55 (t, *J* = 6.3 Hz, 2H), 3.18 (t, *J* = 6.9 Hz, 2H), 2.31 (appar. quint, *J* = 6.6 Hz, 2H), 1.75 (s, 6H). **^{13}C-NMR** (201 MHz, $CDCl_3$) δ 198.2, 146.7, 136.4, 128.9, 125.7, 124.0, 37.5, 36.8, 33.6, 29.1, 26.9. **MS-ESI**(m/z): calculated for $C_{14}H_{17}BrNO^+$ $[(M+H)^+]$ 294.0493; found 294.0. Spectroscopic data is in agreement with[16].

Mechanism – Experiments and Calculations

UV-Vis

To test whether $Fe(acac)_3$ and $BrCCl_3$ could form an electron-donor-acceptor complex (EDA complex) UV-Vis were recorded for the two pure substances in DCE as well as their mixtures. A solution of $Fe(acac)_3$ and $BrCCl_3$ in DCE (0.2 mM and 60 mM respectively) was initially measured. $BrCCl_3$ (300 eq. 17.7 µL) was then added to the cuvette and UV-Vis was once more measured (Mixture 1). A second equal portion of $BrCCl_3$ was added to the same cuvette and UV-Vis recorded once more (Mixture 2). In the plot below the concentration has been corrected for the added volume of $BrCCl_3$. As can be seen in Figure S1 below, no shift of the bands has occurred upon mixing $Fe(acac)_3$ and $BrCCl_3$ and no new charge-transfer bands can be seen. The only difference is a slight decrease in absorbance for Mixture 1 and Mixture 2, which correlates with the slightly diluted solution by the added volume of $BrCCl_3$.

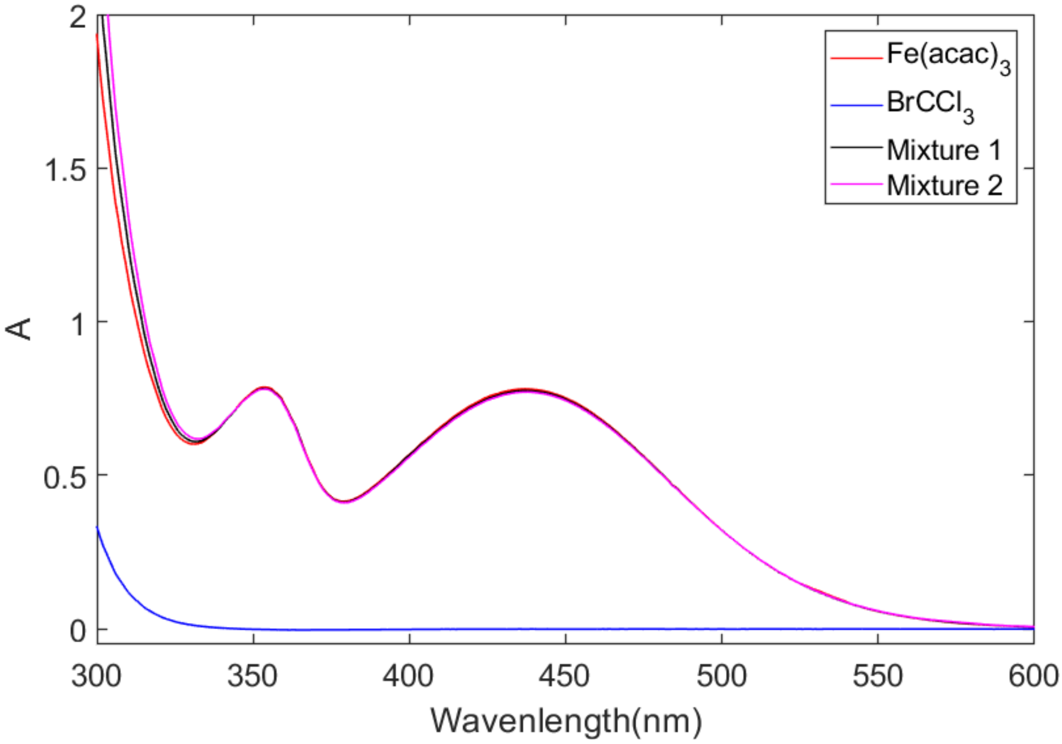

Figure S1. UV-vis absorbance spectroscopy experiments on mixture. No shifts of bands was observed indicating there is no formation of an EDA-complex.

Reactions

Reactions in this section refers to those in Scheme 5 and Scheme 6. All reaction were prepared as described in *Experimental procedure for photoreaction* and conducted at 0.1 mmol scale with respect to **1aa**.

Experiment i): **1aa**, 1 eq. $Fe(acac)_3$ and no $BrCCl_3$ in DCE, irradiated for 6 h. These conditions did not provide any product.

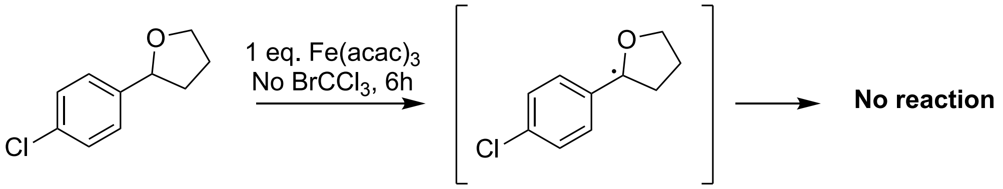

Experiment ii): **1aa**, 3 eq. methyl acrylate, 1 eq. $Fe(acac)_3$ and no $BrCCl_3$ in DCE, irradiated for 6 h. These conditions did not provide any product.

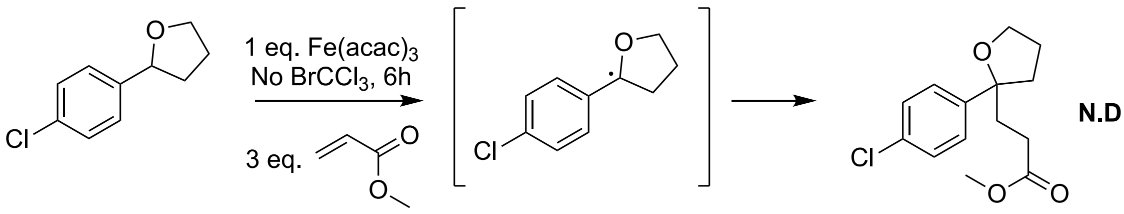

Experiment iii): **1aa**, 1 eq. NBu_4Br, 1 eq. $Fe(acac)_3$ and no $BrCCl_3$ irradiated for 6h. These conditions did not provide any product.

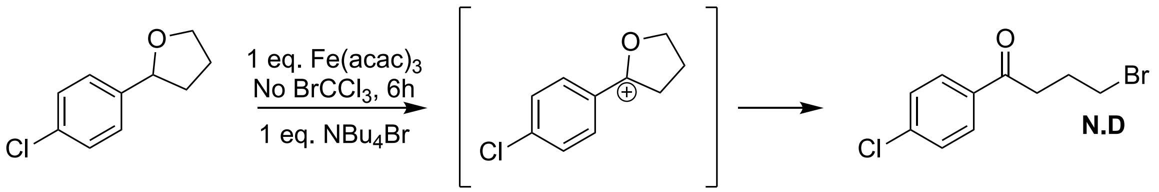

Experiment iv): 0.1 mmol $Fe(acac)_3$ and 1 eq. $BrCCl_3$ was dissolved in 0.5 ml DCE and irradiated for 6 h. These conditions afforded hexachloroethane as detected my GC-MS(EI).

$$Fe(acac)_3 + BrCCl_3 \xrightarrow[\text{DCE, r.t.}]{\text{Blue LEDs}} Cl_3C{-}CCl_3$$

Experiment v): **1aa**, 1 eq. TEMPO, 1 eq. $Fe(acac)_3$, 3 eq. $BrCCl_3$ in DCE, irradiated for 6h. These conditions did not provide any product.

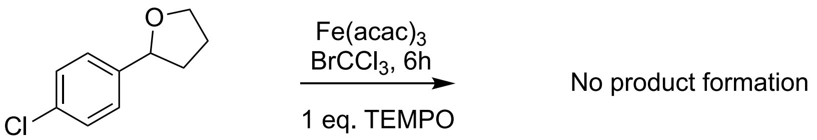

Calculations on $BrCCl_3$

Geometry optimization was performed with the method CCSD+PCM/cc-pVTZ, solvent: dichloroethane.

Optimized geometry (Å):

C	0.00000	0.00000	-0.40878
Br	0.00000	0.00000	1.52862
Cl	0.00000	1.66949	-1.00096
Cl	1.44582	-0.83474	-1.00096
Cl	-1.44582	-0.83474	-1.00096

Energy: -3989.827393 Hartree

Single-point calculations:

Performed with the method (R/U)CCSD(T)+PCM/aug-cc-pVQZ, solvent: dichloroethane.

Energy $^1A_1$: -3990.085935 Hartree
Energy $^3A_1$: -3989.868337 Hartree

Jablonski diagram

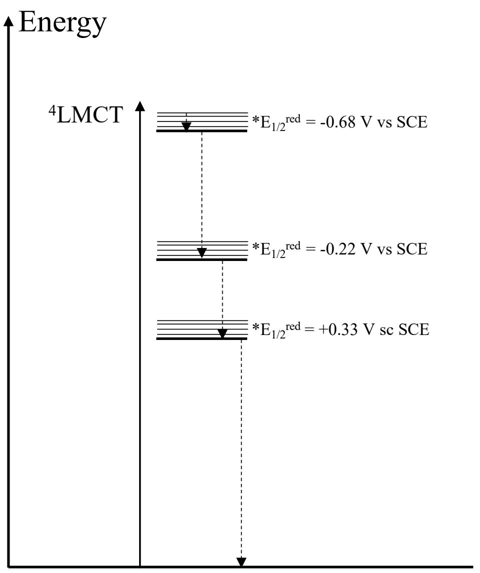

Figure S2. Schematic Jablonski diagram of the some excited states of $Fe(acac)_3$.

References

[1] P. H. Huy, A. M. P. Koskinen, *Org. Lett.* **2013**, *15*, 5178–5181.

[2] D. Liu, C. Liu, H. Li, A. Lei, *Angew. Chemie - Int. Ed.* **2013**, *52*, 4453–4456.

[3] B. J. Shields, A. G. Doyle, *J. Am. Chem. Soc.* **2016**, *138*, 12719–12722.

[4] D. R. Heitz, J. C. Tellis, G. A. Molander, *J. Am. Chem. Soc.* **2016**, *138*, 12715–12718.

[5] W. Li, C. Yang, G. L. Gao, W. Xia, *Synlett* **2016**, *27*, 1391–1396.

[6] S. Estopiñá-Durán, L. J. Donnelly, E. B. Mclean, B. M. Hockin, A. M. Z. Slawin, J. E. Taylor, *Chem. - A Eur. J.* **2019**, *25*, 3950–3956.

[7] Z. Xie, Y. Cai, H. Hu, C. Lin, J. Jiang, Z. Chen, L. Wang, Y. Pan, *Org. Lett.* **2013**, *15*, 4600–4603.

[8] A. R. Reddy, C. Y. Zhou, Z. Guo, J. Wei, C. M. Che, *Angew. Chemie - Int. Ed.* **2014**, *53*, 14175–14180.

[9] H. Im, D. Kang, S. Choi, S. Shin, S. Hong, *Org. Lett.* **2018**, *20*, 7437–7441.

[10] S. Fredrich, A. Bonasera, V. Valderrey, S. Hecht, *J. Am. Chem. Soc.* **2018**, *140*, 6432–6440.

[11] C. Wang, M. Gong, M. Huang, Y. Li, J. K. Kim, Y. Wu, *Tetrahedron* **2016**, *72*, 7931–7936.

[12] R. Zhao, Y. Yao, D. Zhu, D. Chang, Y. Liu, L. Shi, *Org. Lett.* **2018**, *20*, 1228–1231.

[13] X. Fan, H. Zhao, J. Yu, X. Bao, C. Zhu, *Org. Chem. Front.* **2016**, *3*, 227–232.

[14] C. Liu, J. Wang, L. Meng, Y. Deng, Y. Li, A. Lei, *Angew. Chemie* **2011**, *123*, 5250–5254.

[15] J.-N. Xiang, X. Dai, C. X. Zhou, J. Li, M. Q. Nguyen, *Patent: US 20090326067A1*, **2009**.

[16] J. Huang, W. Wang, L. Wang, *Org. Process Res. Dev.* **2010**, *14*, 4–8.

Appendix – Experimental NMR-Spectra

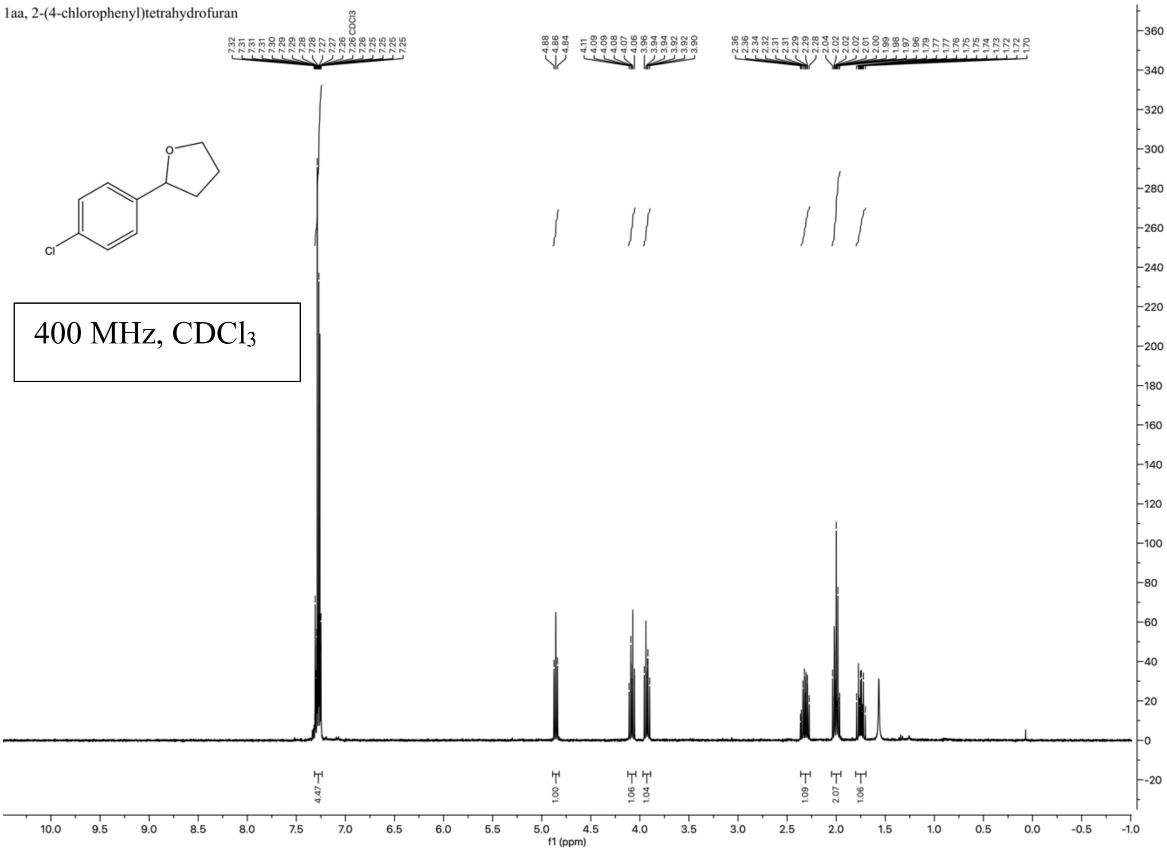

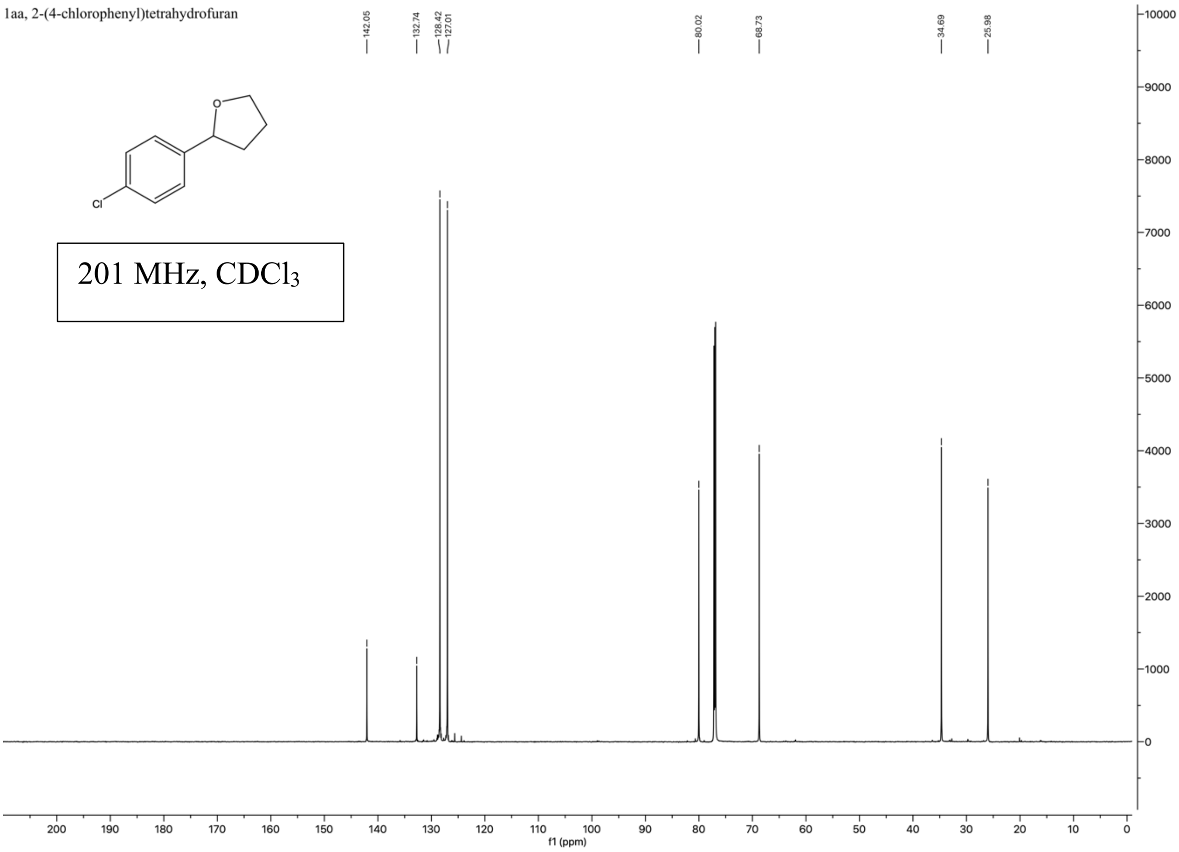

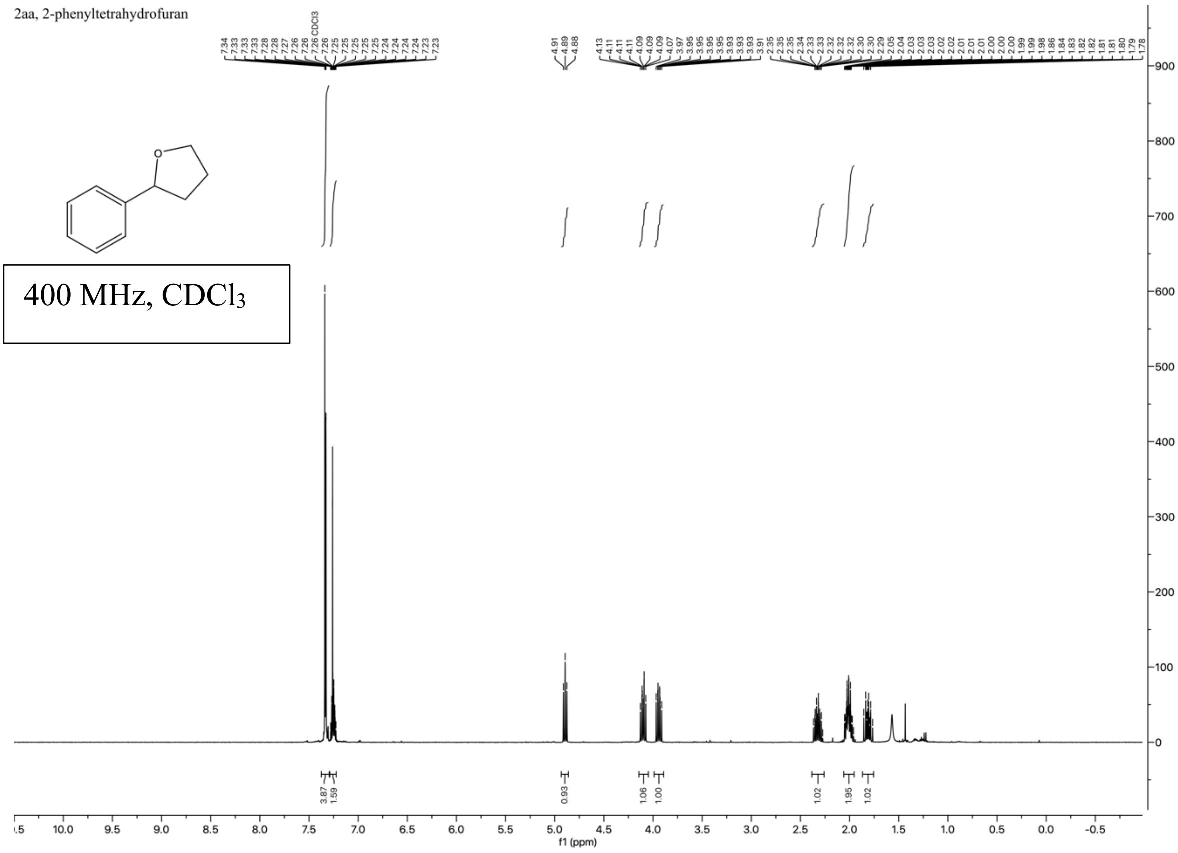
2aa, 2-phenyltetrahydrofuran
400 MHz, CDCl3
f1 (ppm)

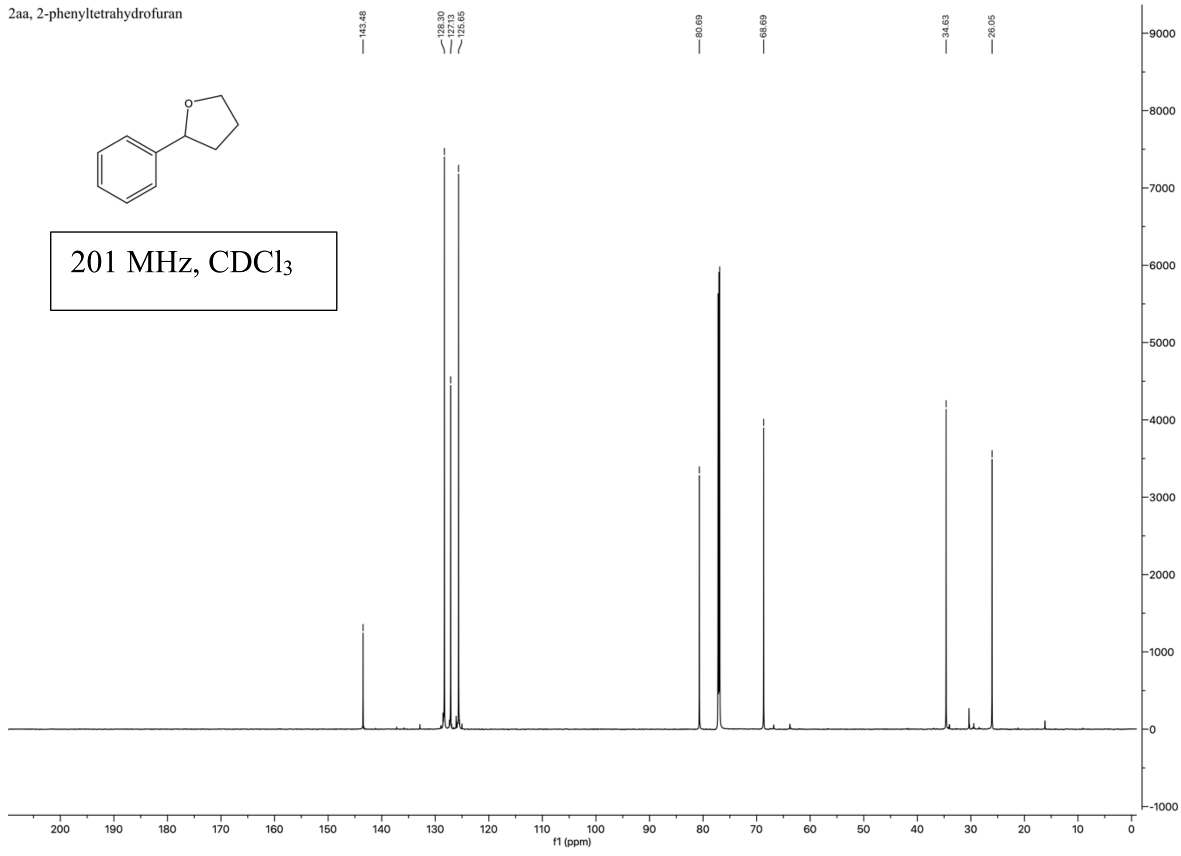
2aa, 2-phenyltetrahydrofuran
143.48
128.30
127.13
125.65
80.69
68.69
34.63
26.05
201 MHz, CDCl3
f1 (ppm)

2bb, 2-([1,1'-biphenyl]-4-yl)tetrahydrofuran

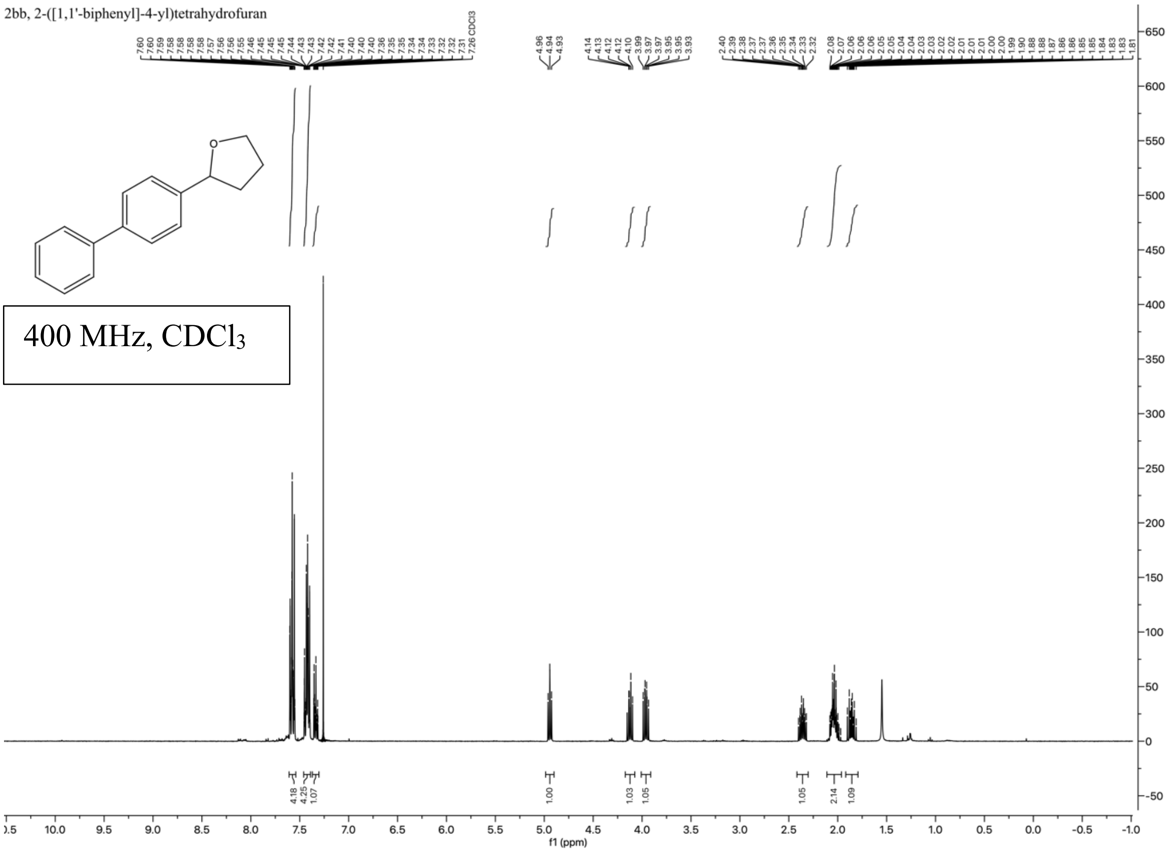

2bb, 2-([1,1'-biphenyl]-4-yl)tetrahydrofuran

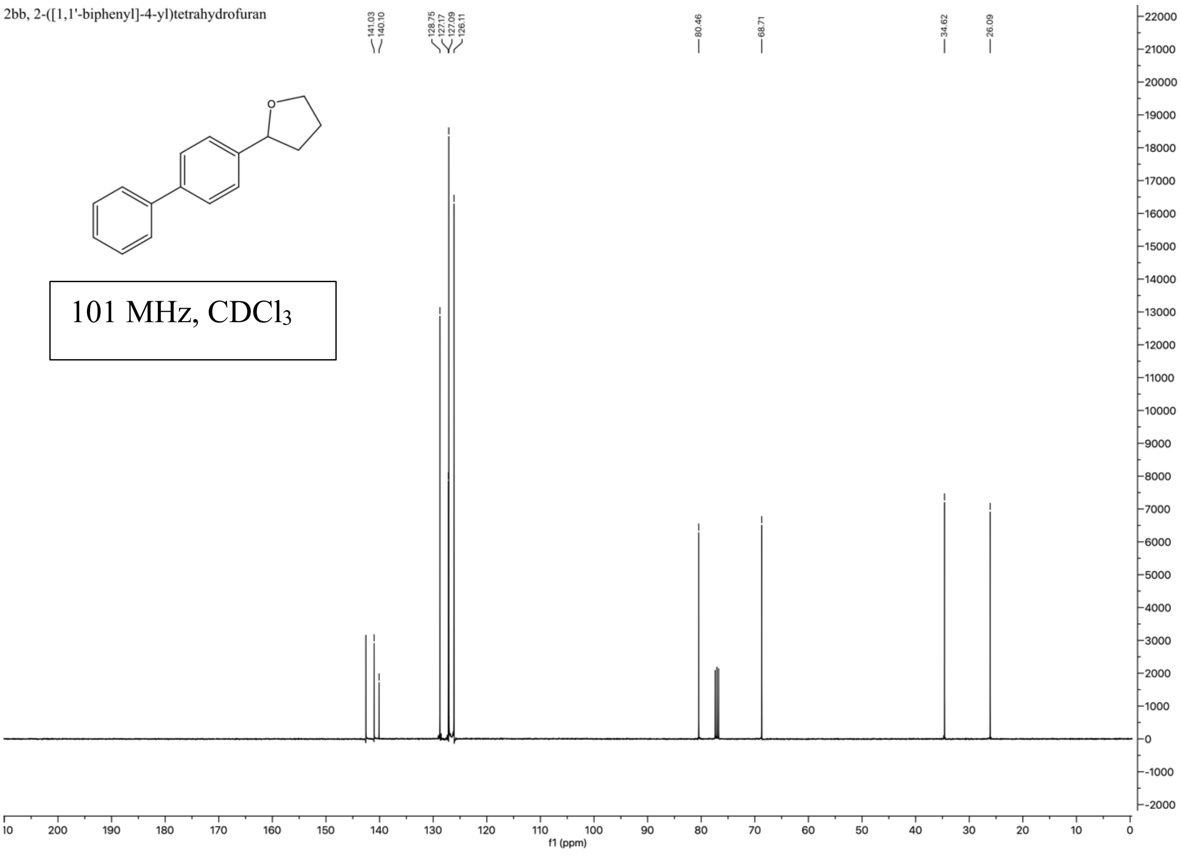

2cc, 4-(tetrahydrofuran-2-yl)benzonitrile

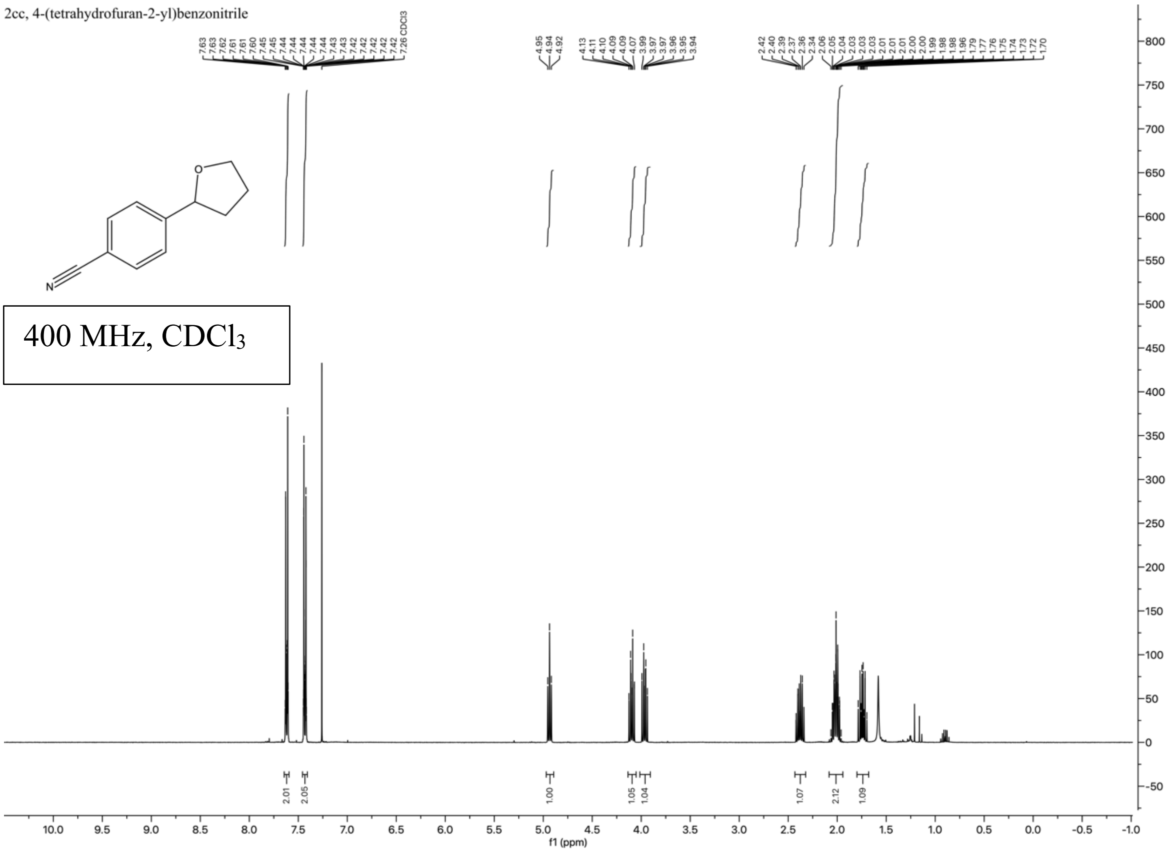

2cc, 4-(tetrahydrofuran-2-yl)benzonitrile

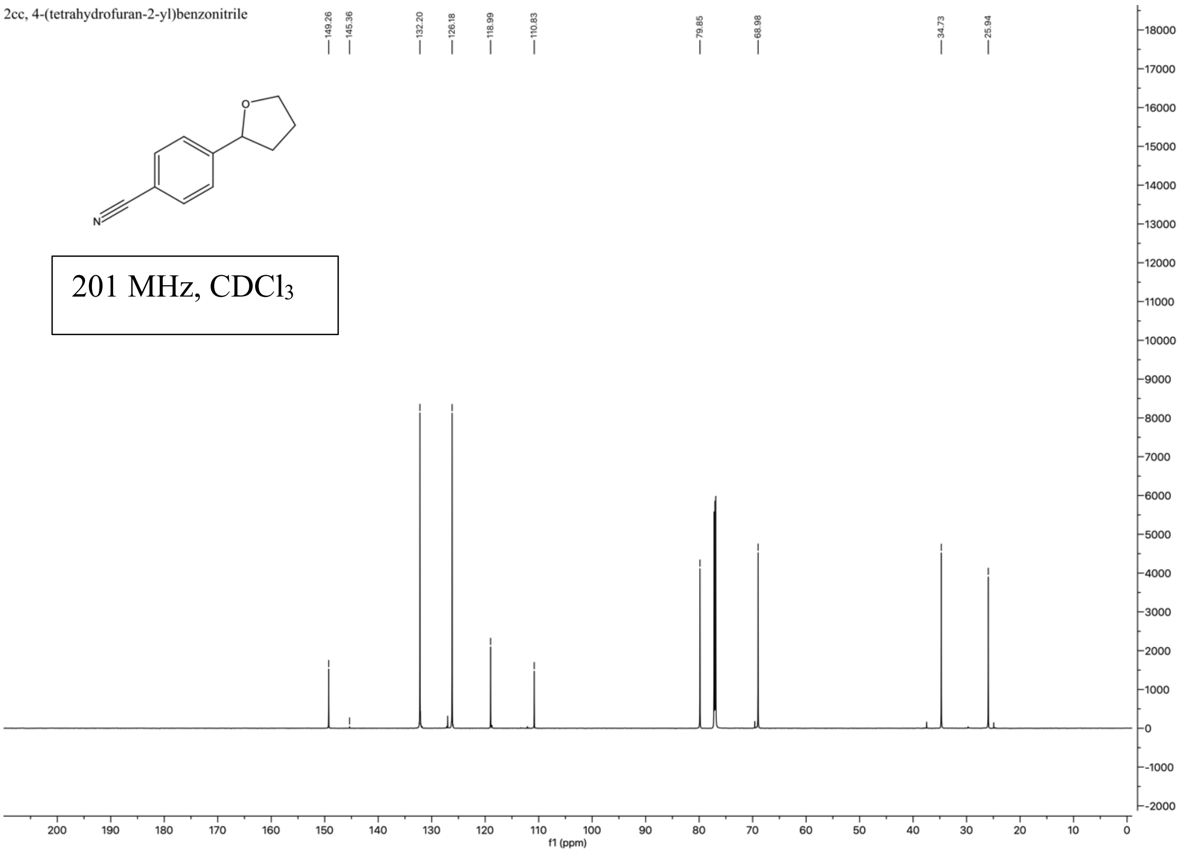

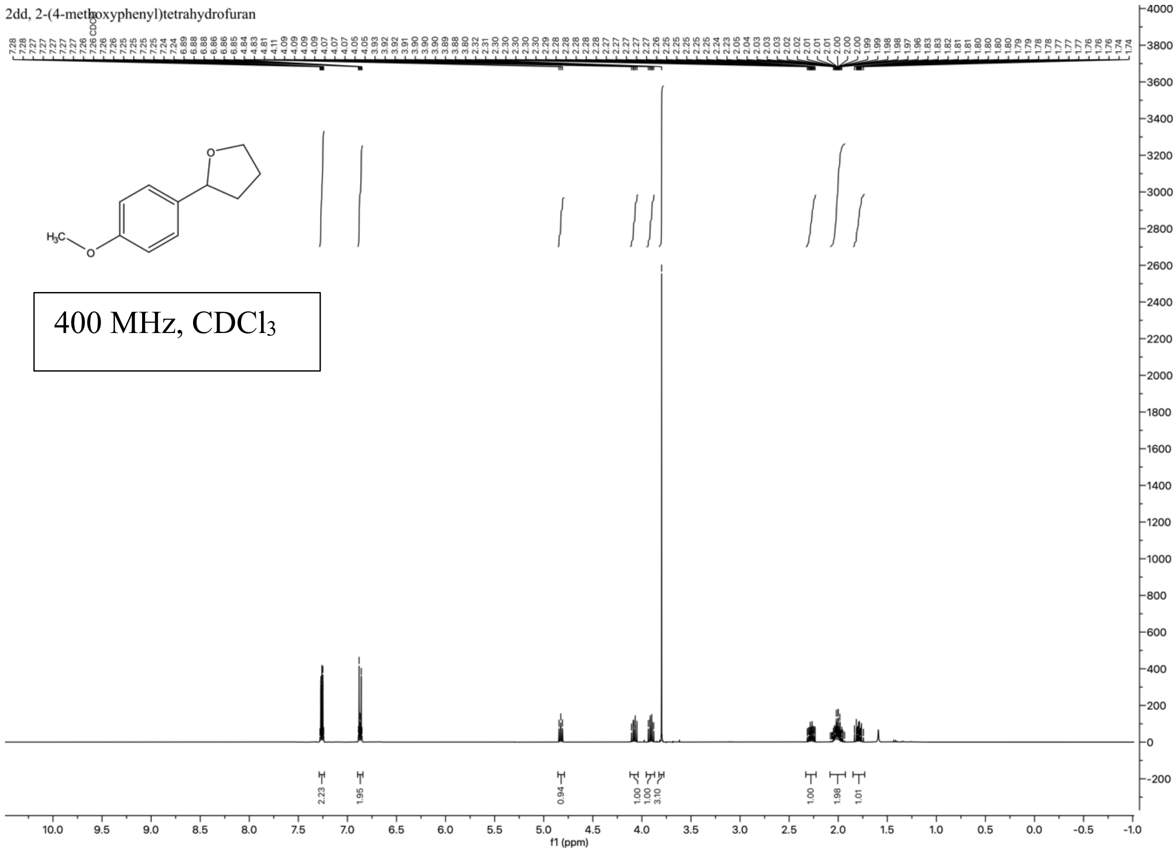
2dd, 2-(4-methoxyphenyl)tetrahydrofuran
400 MHz, CDCl3
f1 (ppm)

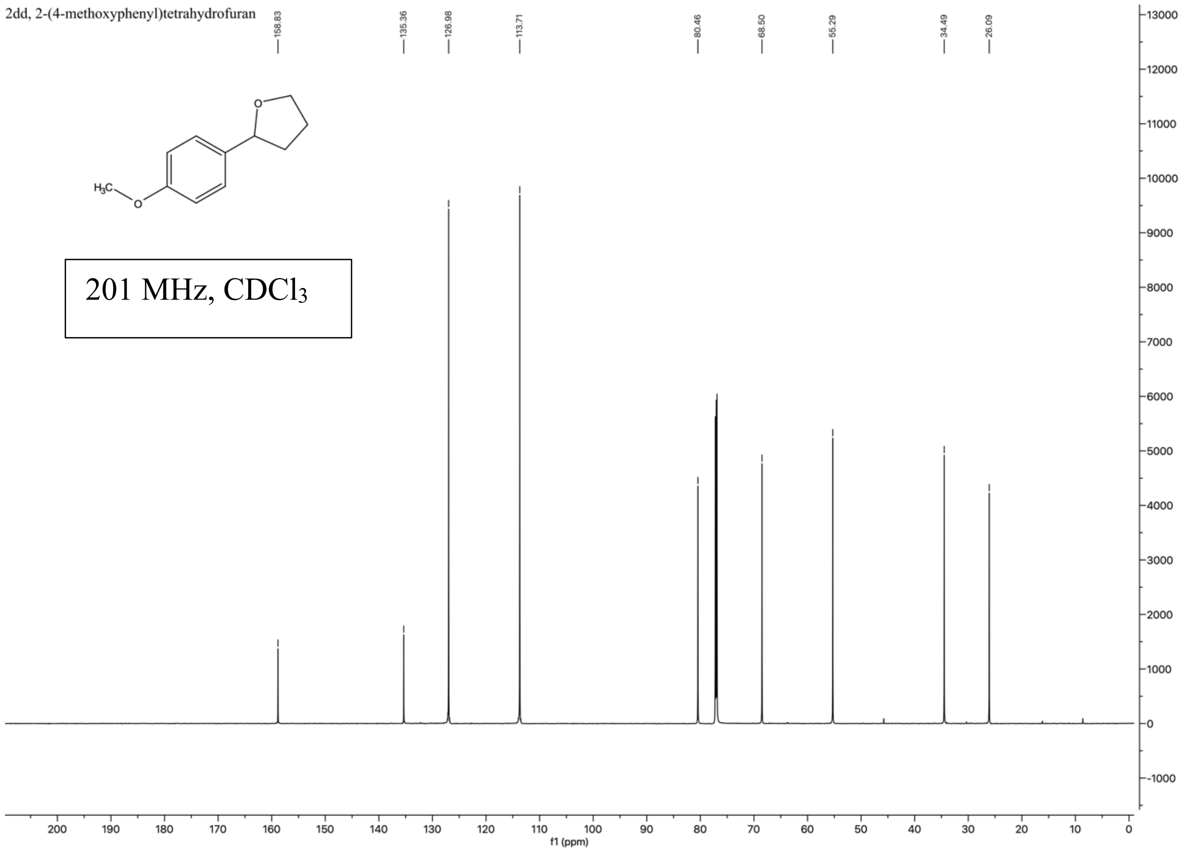
2dd, 2-(4-methoxyphenyl)tetrahydrofuran
158.83
135.36
126.98
113.71
80.46
68.50
55.29
34.49
26.09
201 MHz, CDCl3
f1 (ppm)

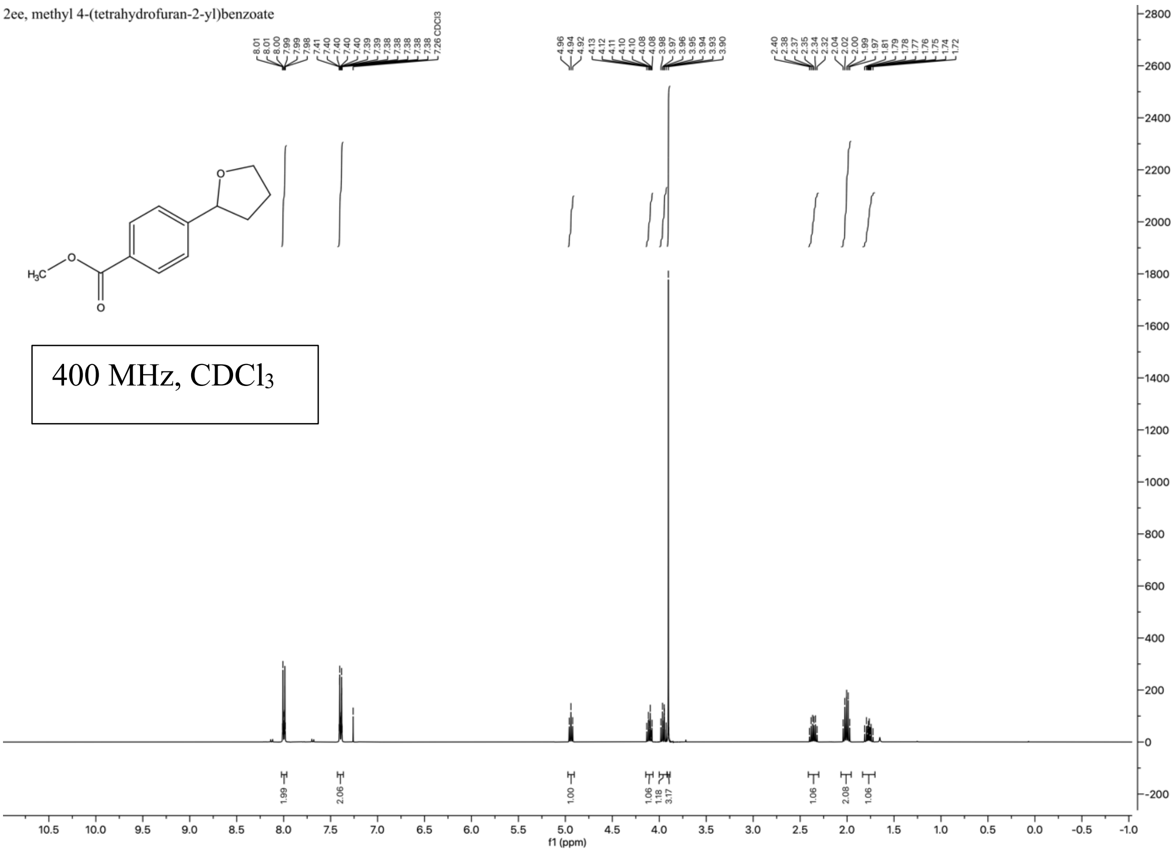
2ee, methyl 4-(tetrahydrofuran-2-yl)benzoate
400 MHz, CDCl3
f1 (ppm)

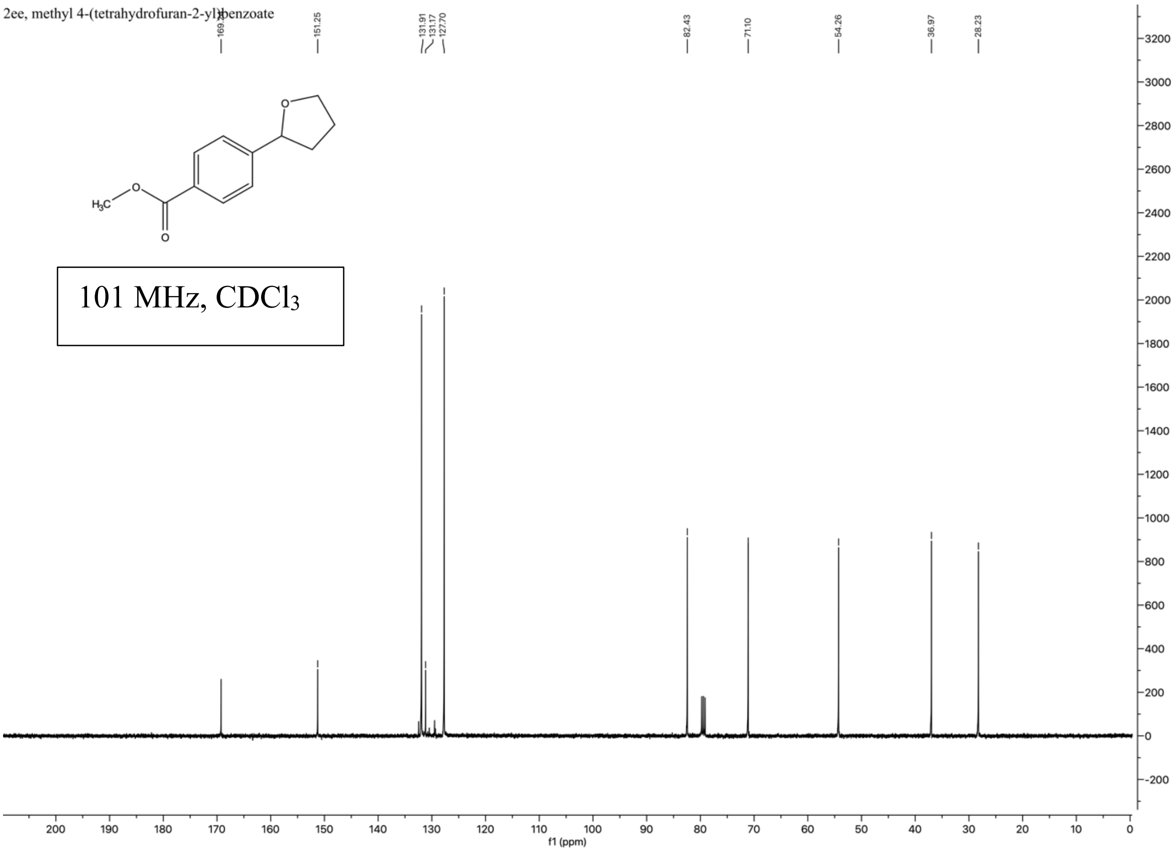
2ee, methyl 4-(tetrahydrofuran-2-yl)benzoate
151.25
131.91
131.17
127.70
82.43
71.10
54.26
36.97
28.23
101 MHz, CDCl3
f1 (ppm)

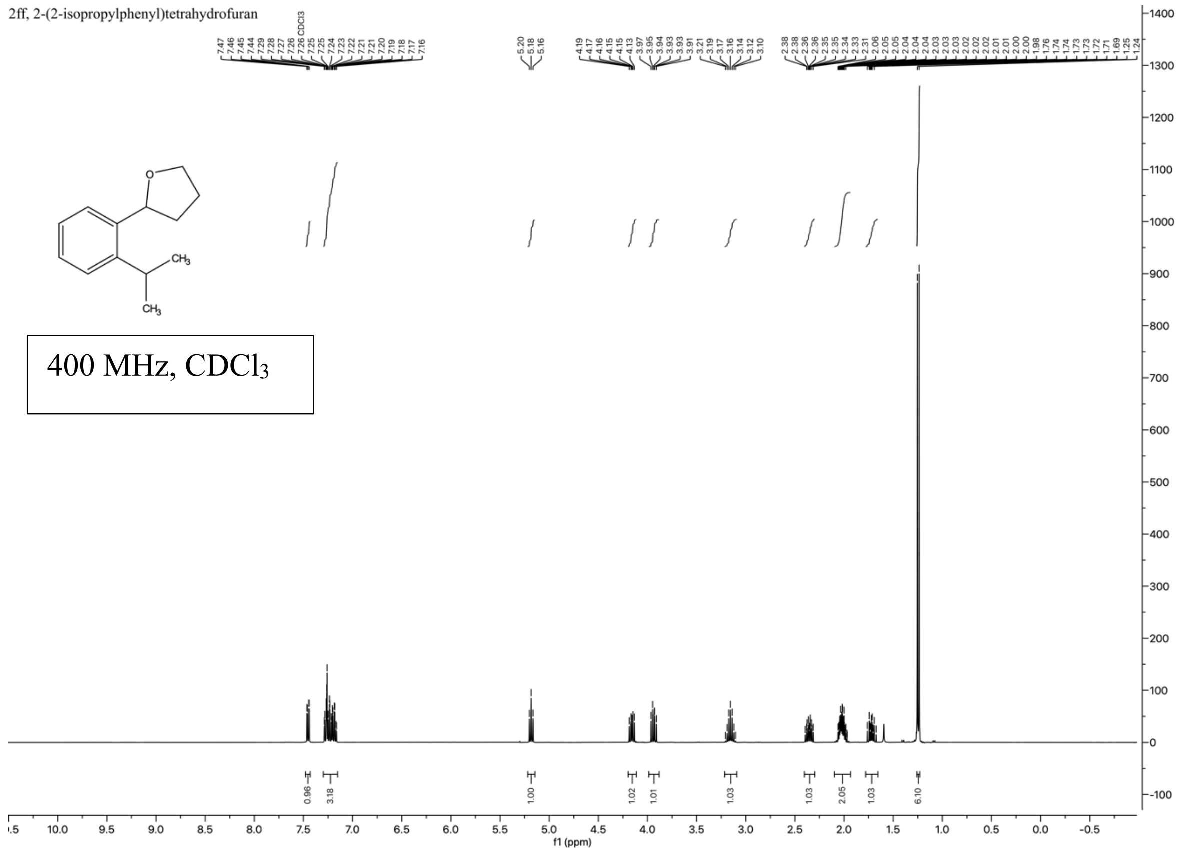
2ff, 2-(2-isopropylphenyl)tetrahydrofuran
400 MHz, CDCl3
f1 (ppm)

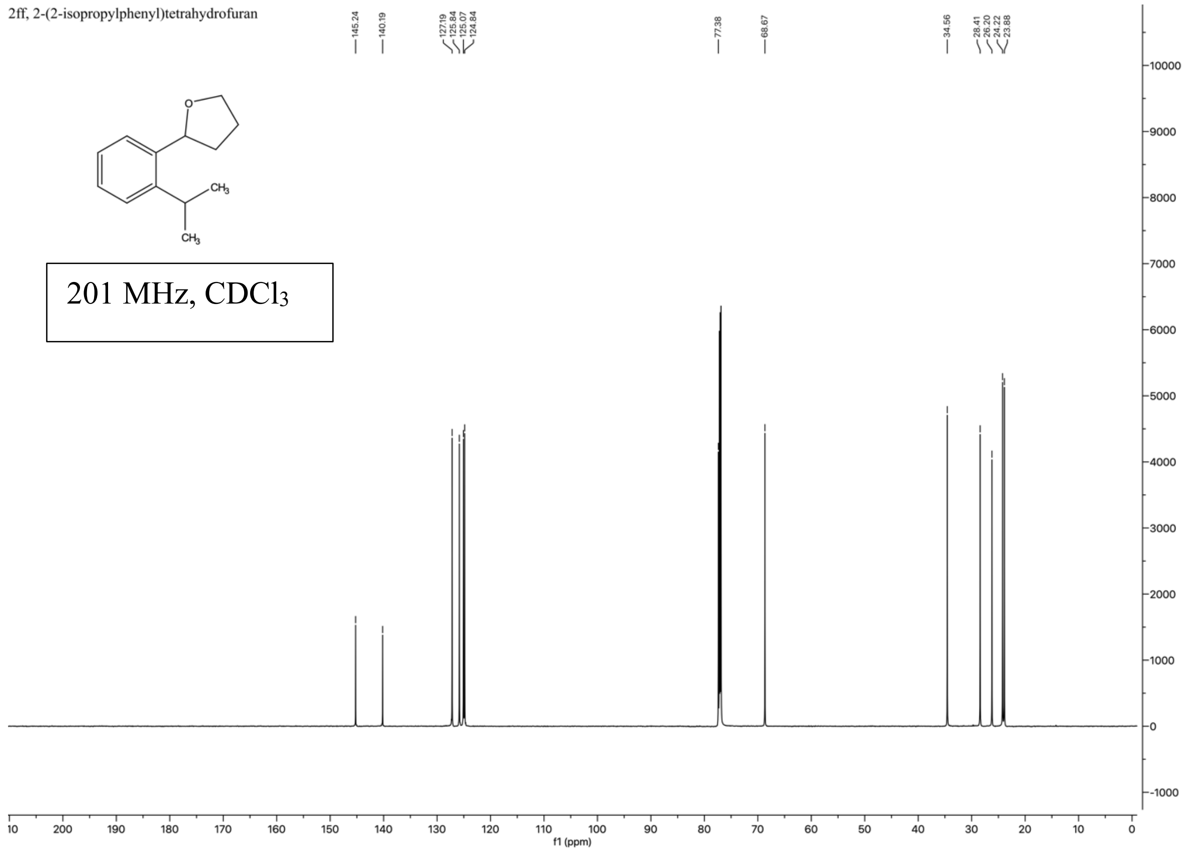
2ff, 2-(2-isopropylphenyl)tetrahydrofuran
145.24
140.19
127.19
125.84
125.07
124.84
77.38
68.67
34.56
28.41
26.20
24.22
23.88
201 MHz, CDCl3
f1 (ppm)

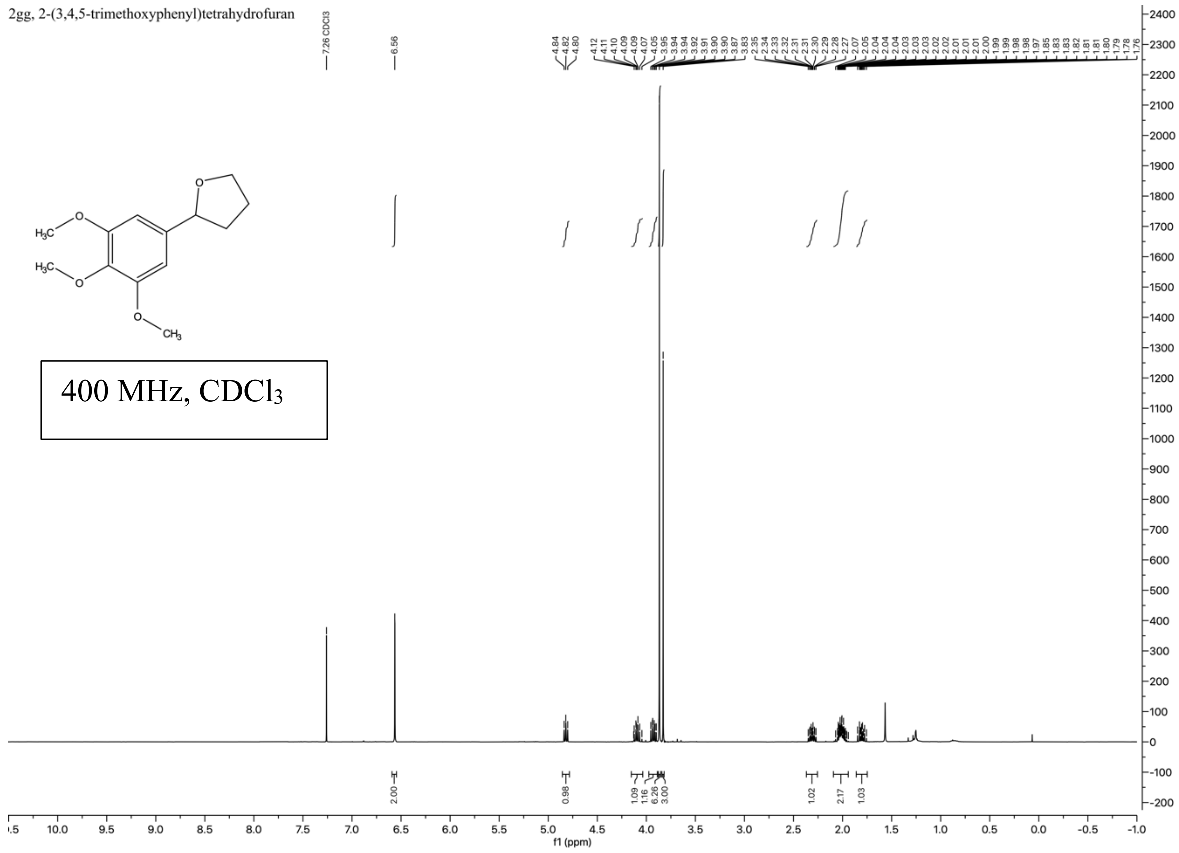
2gg, 2-(3,4,5-trimethoxyphenyl)tetrahydrofuran
400 MHz, CDCl3
f1 (ppm)

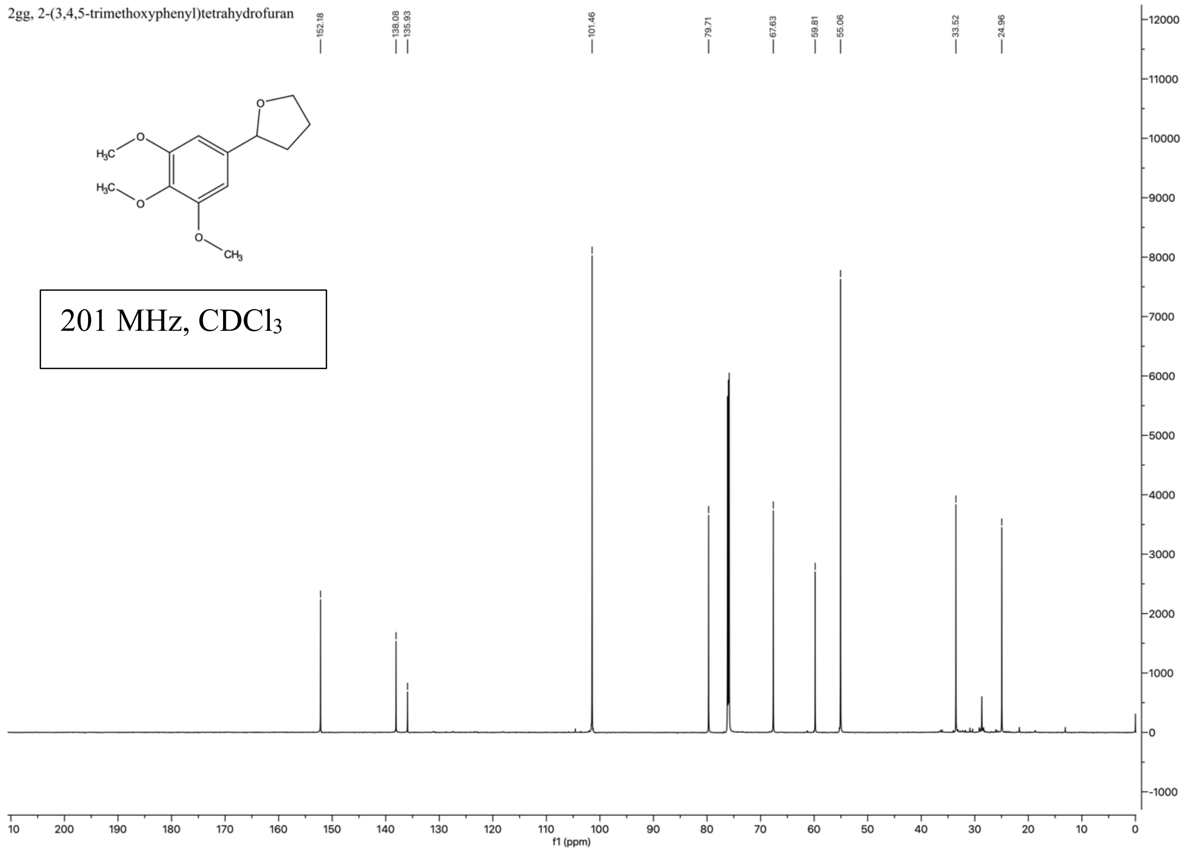
2gg, 2-(3,4,5-trimethoxyphenyl)tetrahydrofuran
201 MHz, CDCl3
f1 (ppm)

2hh, 2-(4-bromophenyl)tetrahydrofuran

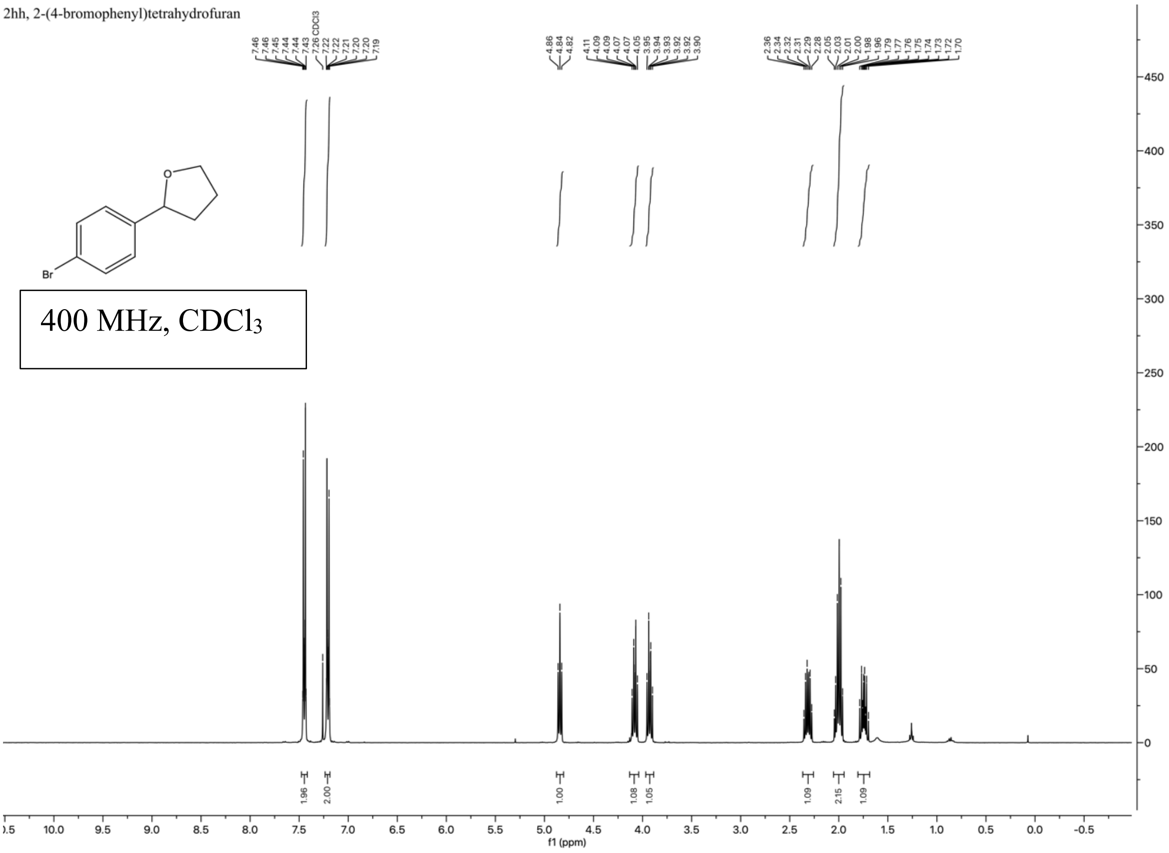

2hh, 2-(4-bromophenyl)tetrahydrofuran

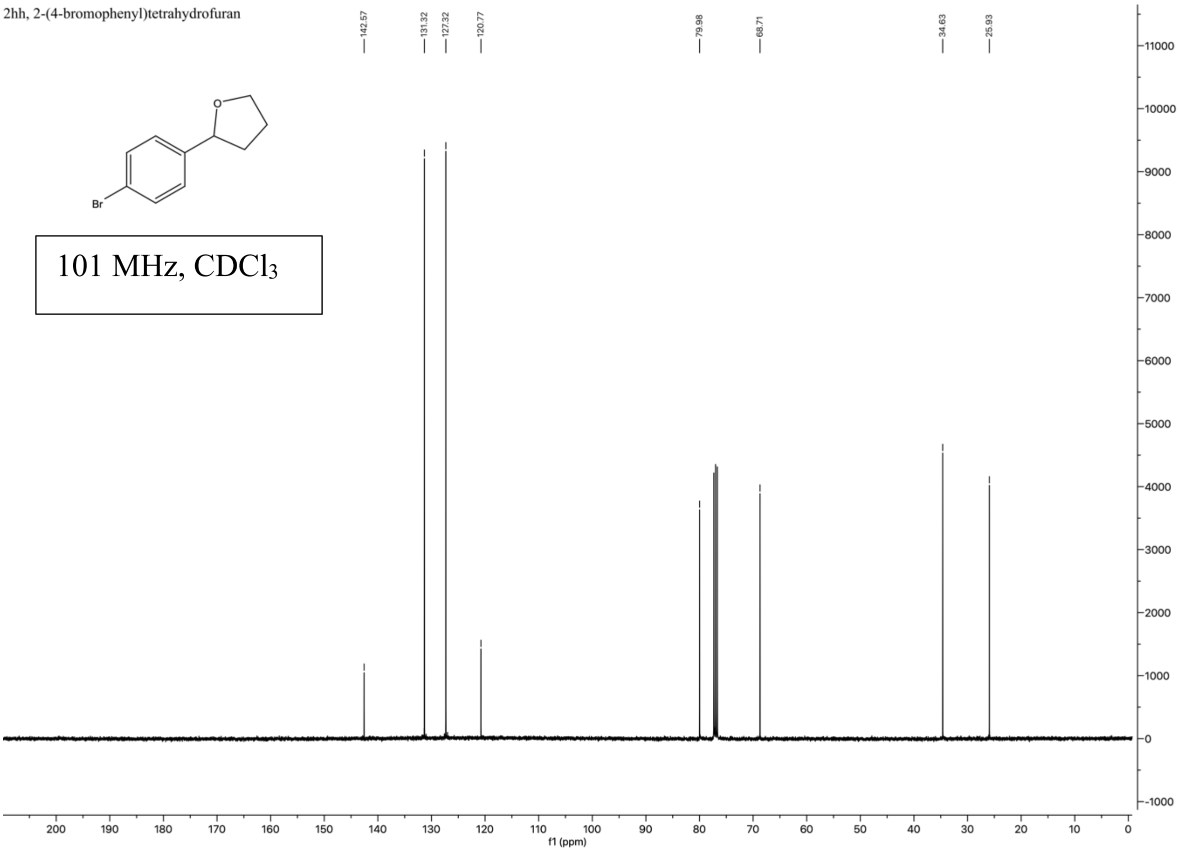

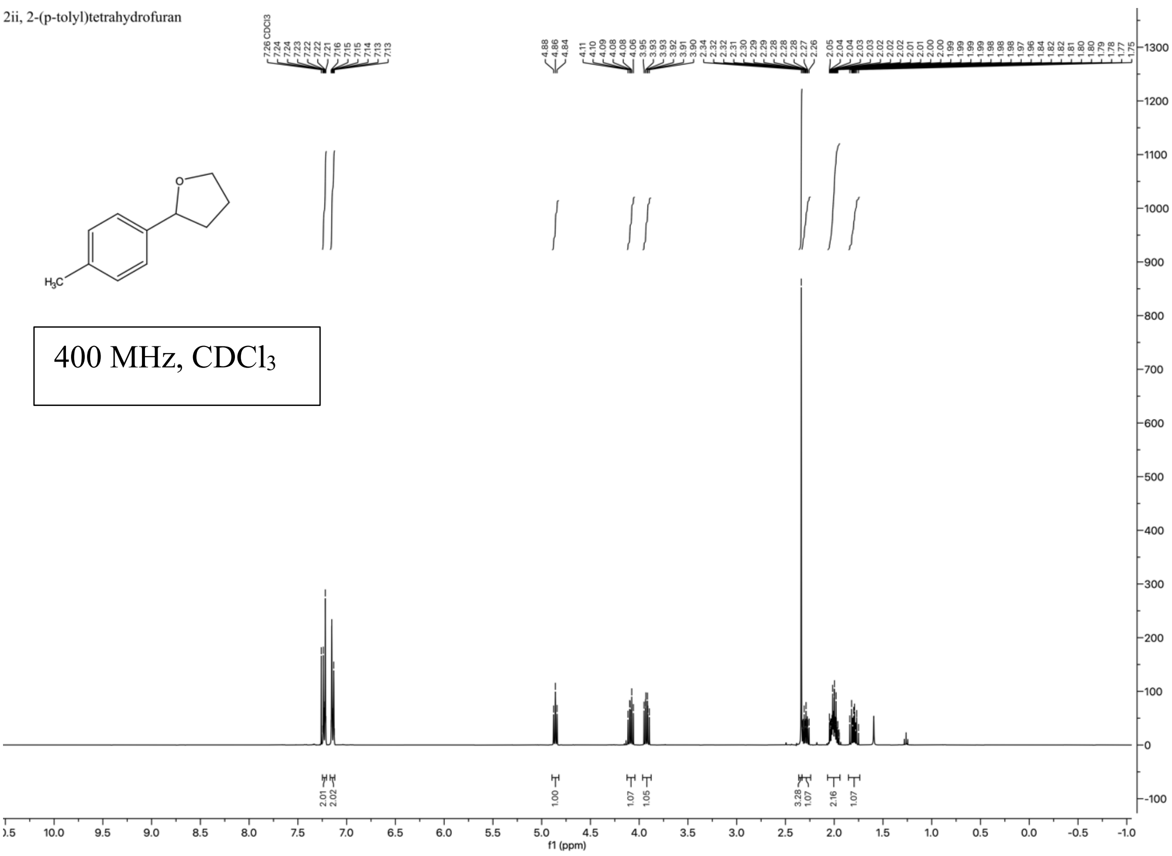
2ii, 2-(p-tolyl)tetrahydrofuran
O
H3C
400 MHz, CDCl3
f1 (ppm)

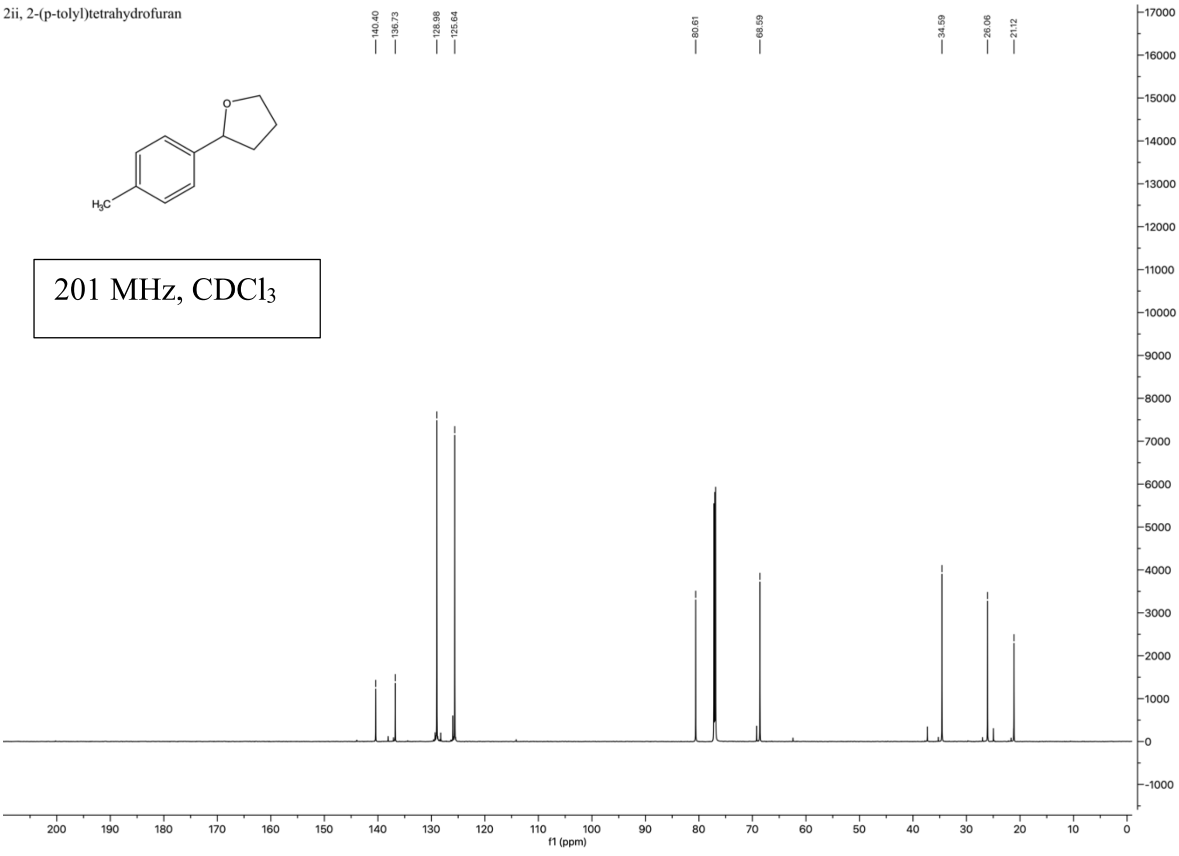
2ii, 2-(p-tolyl)tetrahydrofuran
140.40
136.73
128.98
125.64
80.61
68.59
34.59
26.06
21.12
O
H3C
201 MHz, CDCl3
f1 (ppm)

2jj, 2-(2,4-difluorophenyl)tetrahydrofuran

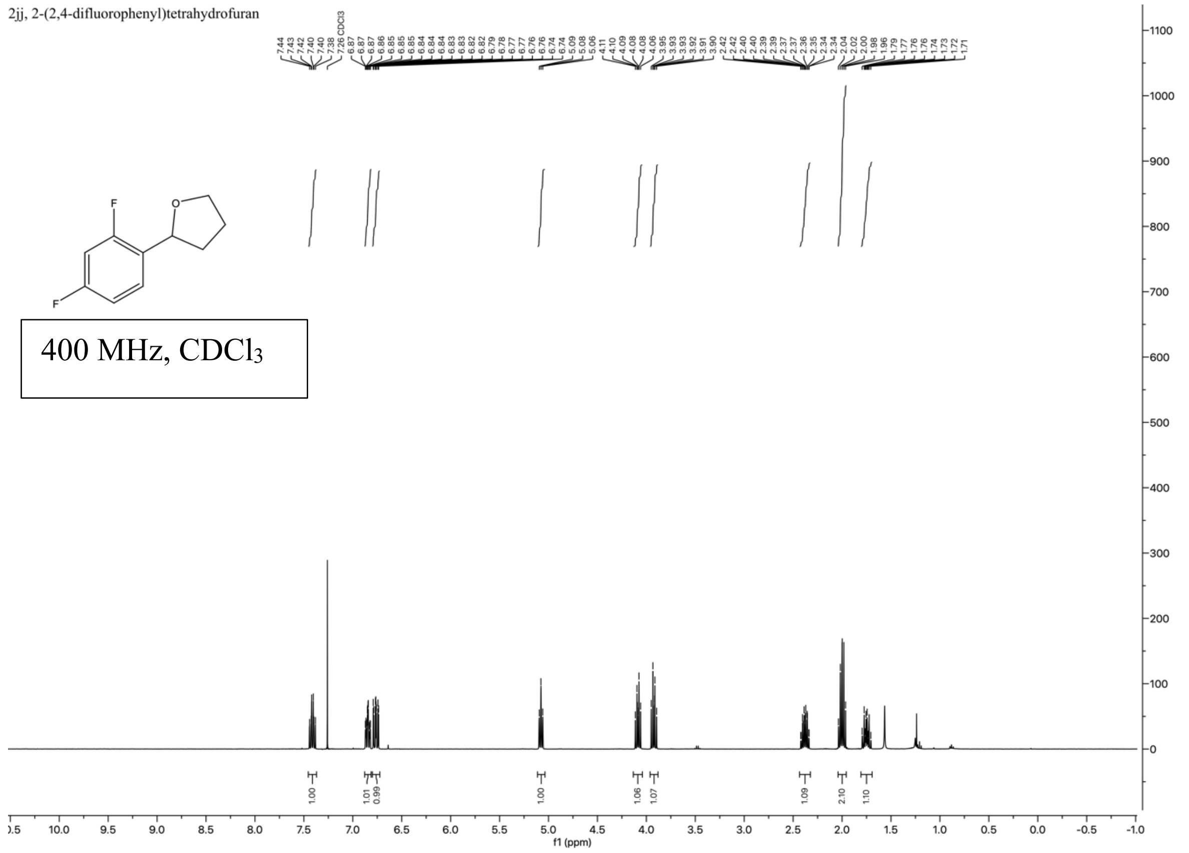

2jj, 2-(2,4-difluorophenyl)tetrahydrofuran

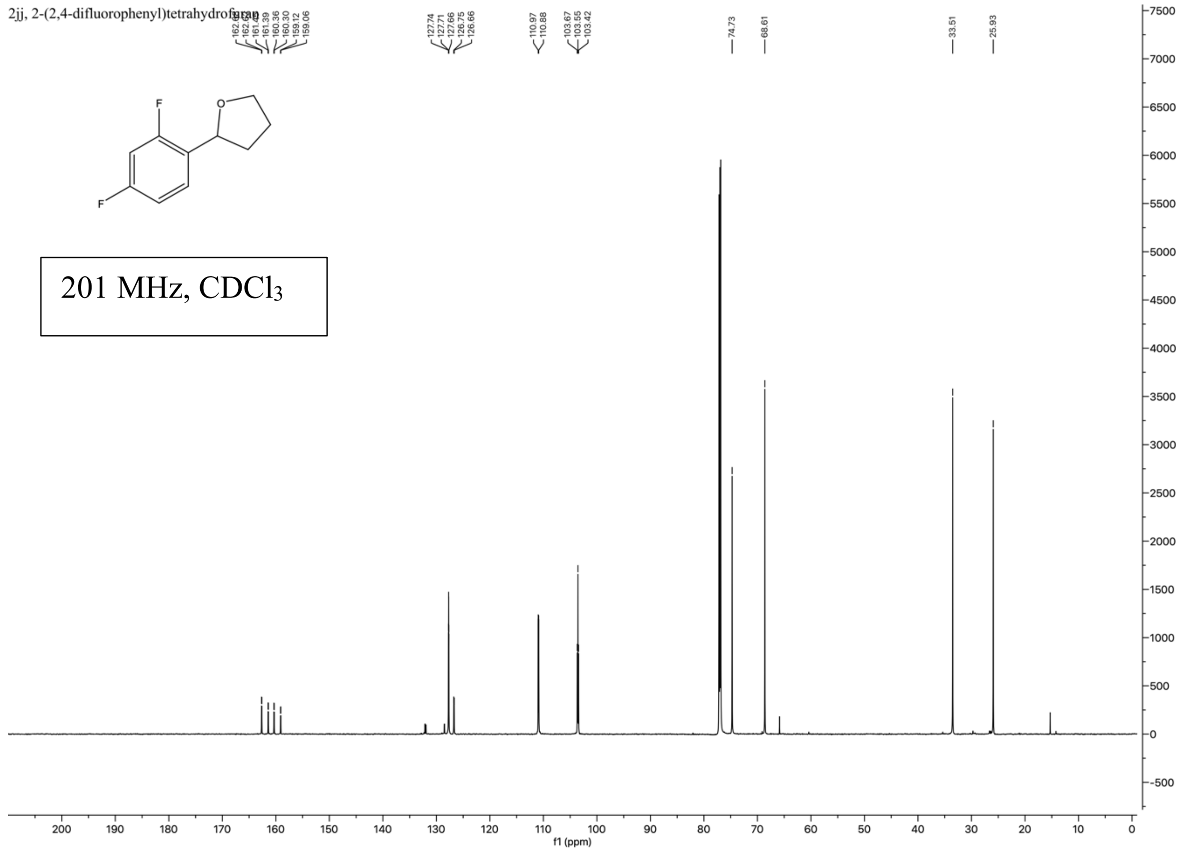

2jj, 2-(2,4-difluorophenyl)tetrahydrofuran

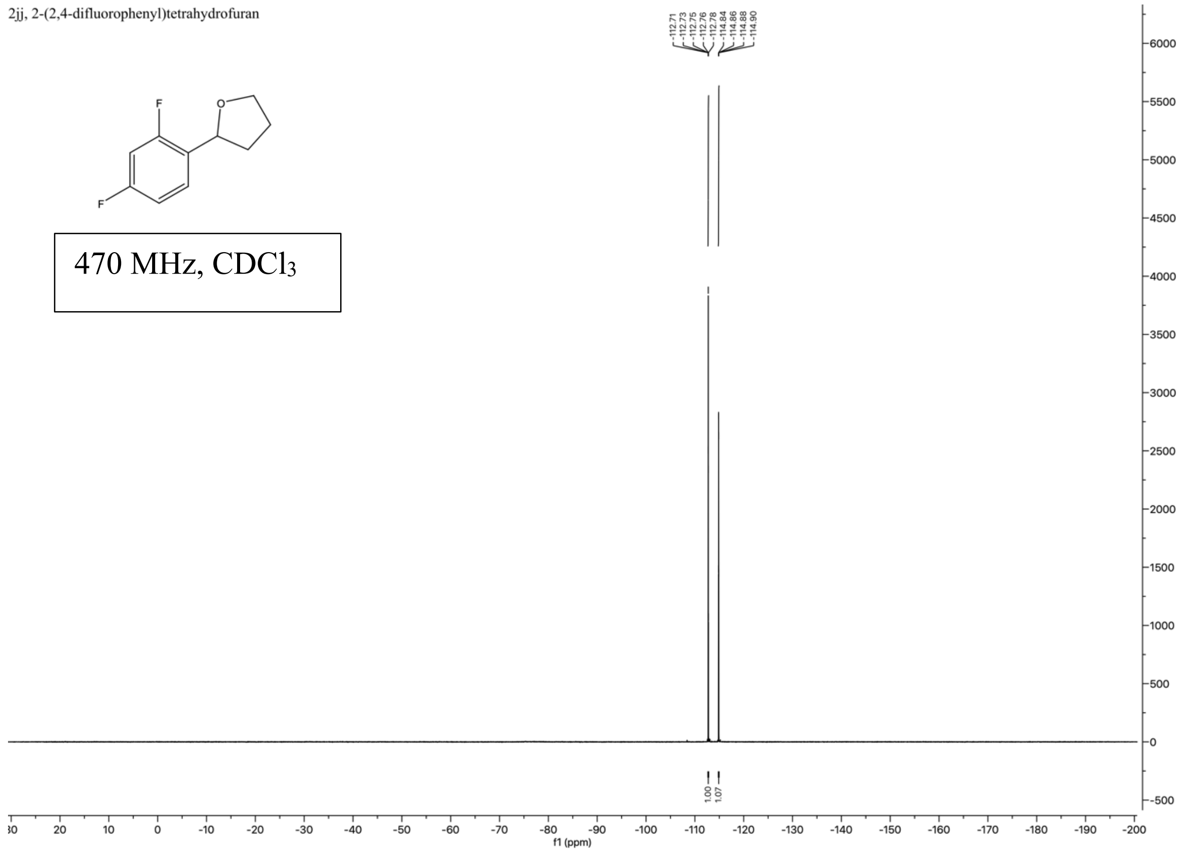

2kk, 2-(3-methoxyphenyl)tetrahydrofuran

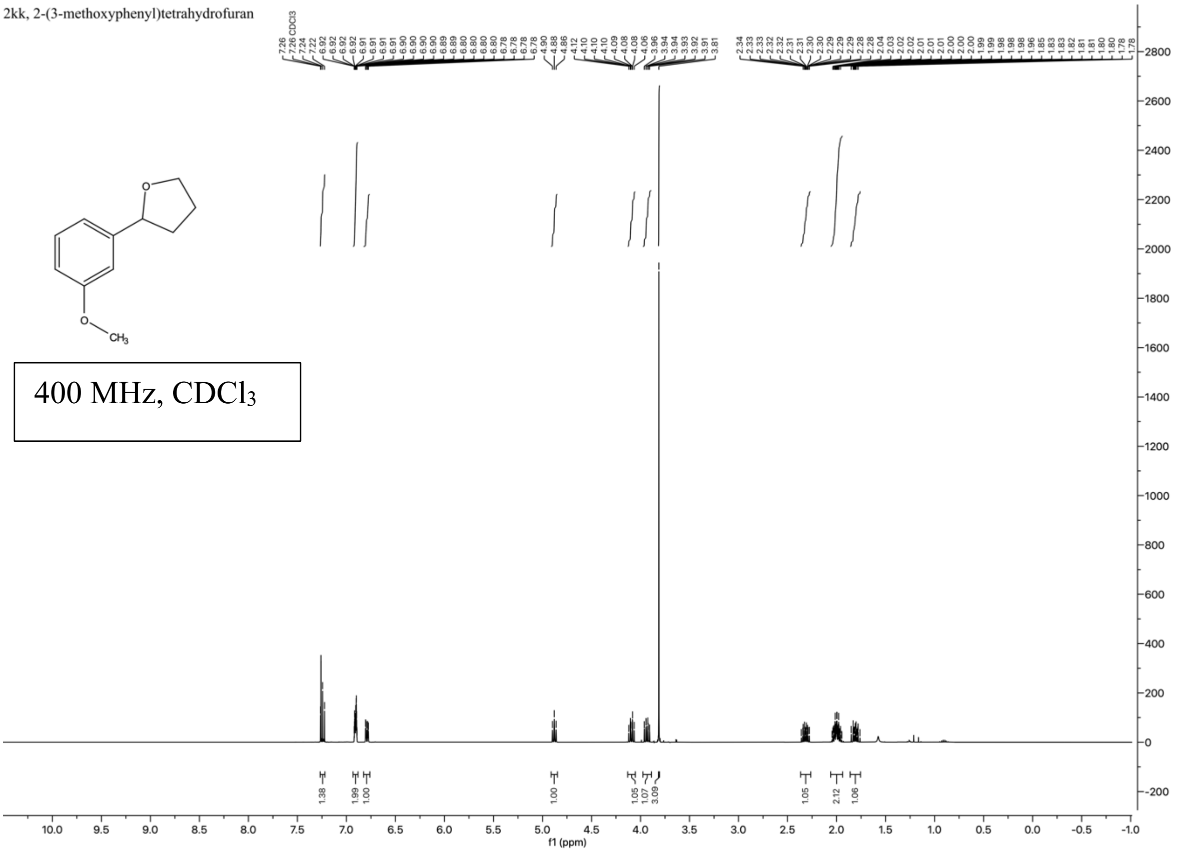

400 MHz, $CDCl_3$

2kk, 2-(3-methoxyphenyl)tetrahydrofuran

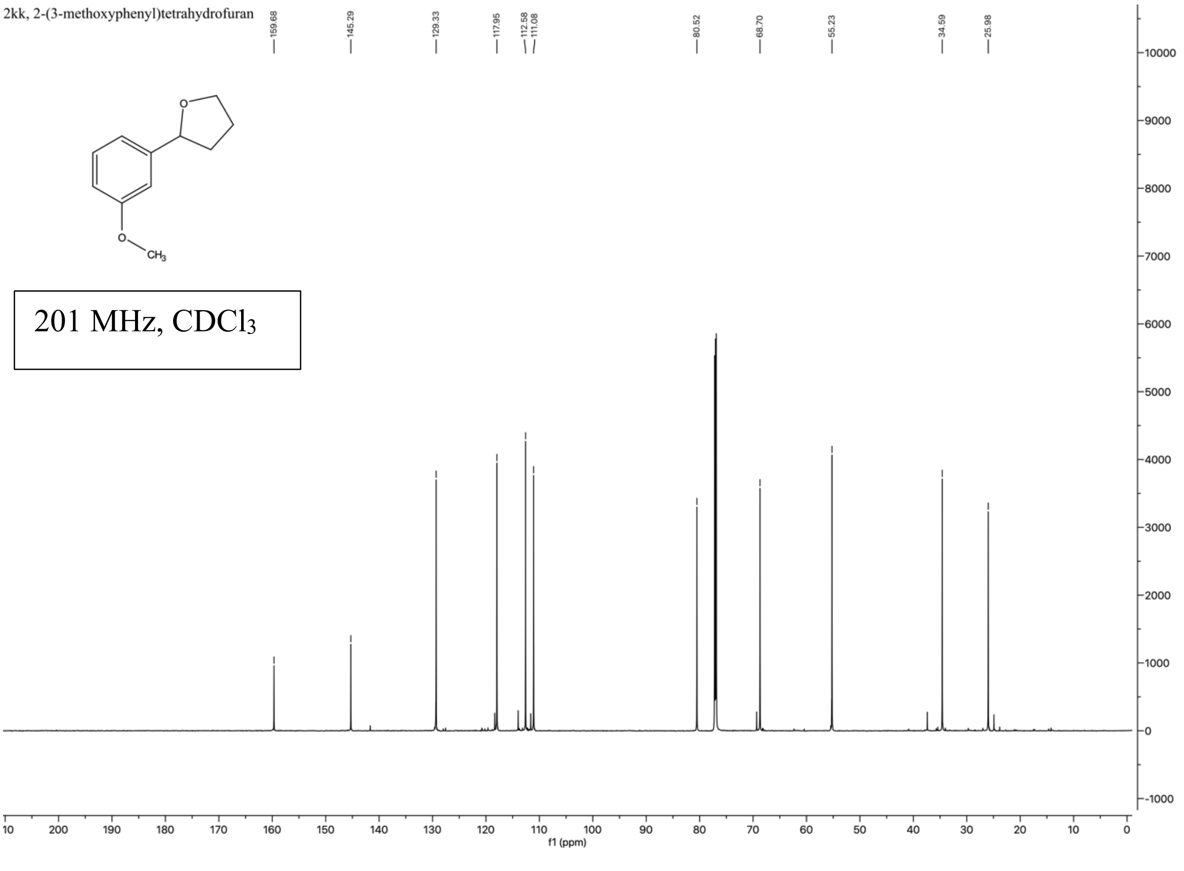

201 MHz, $CDCl_3$

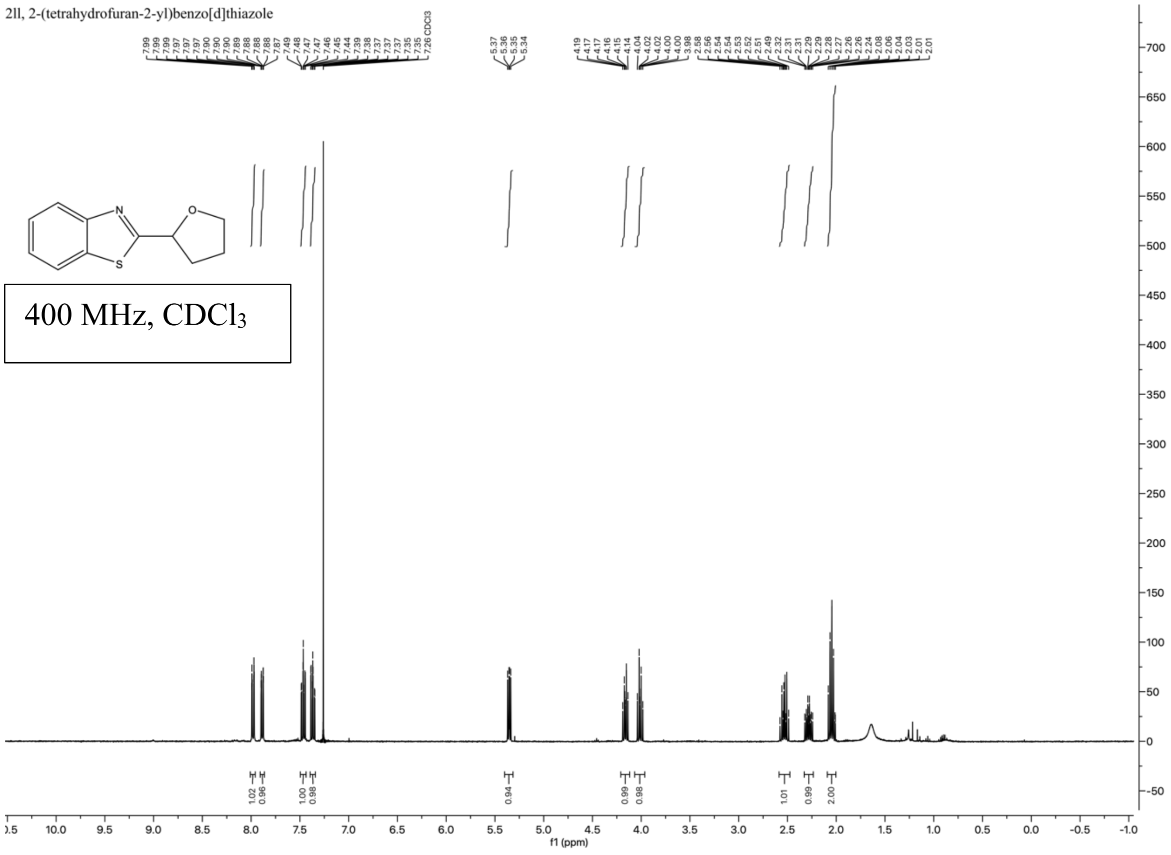
2ll, 2-(tetrahydrofuran-2-yl)benzo[d]thiazole
400 MHz, CDCl3
f1 (ppm)

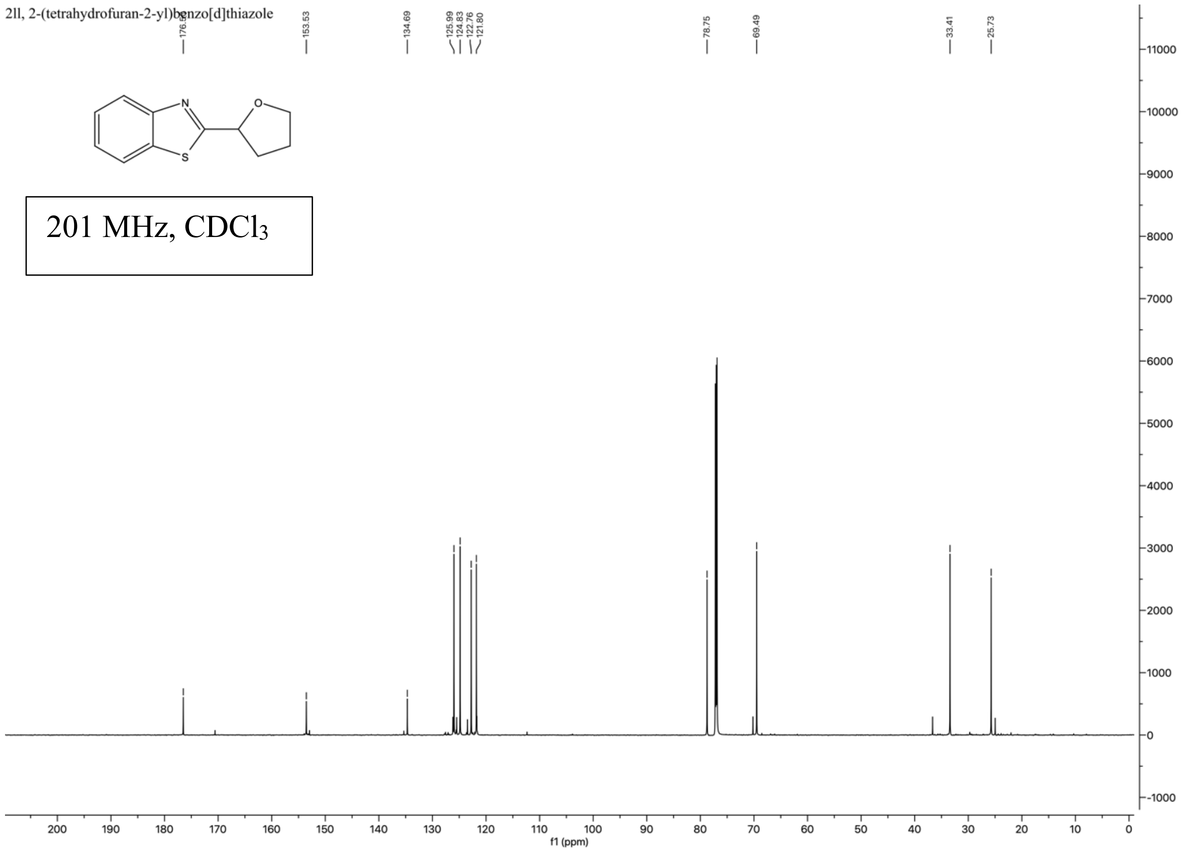
2ll, 2-(tetrahydrofuran-2-yl)benzo[d]thiazole
153.53
134.69
125.99
124.83
122.76
121.80
78.75
69.49
33.41
25.73
201 MHz, CDCl3
f1 (ppm)

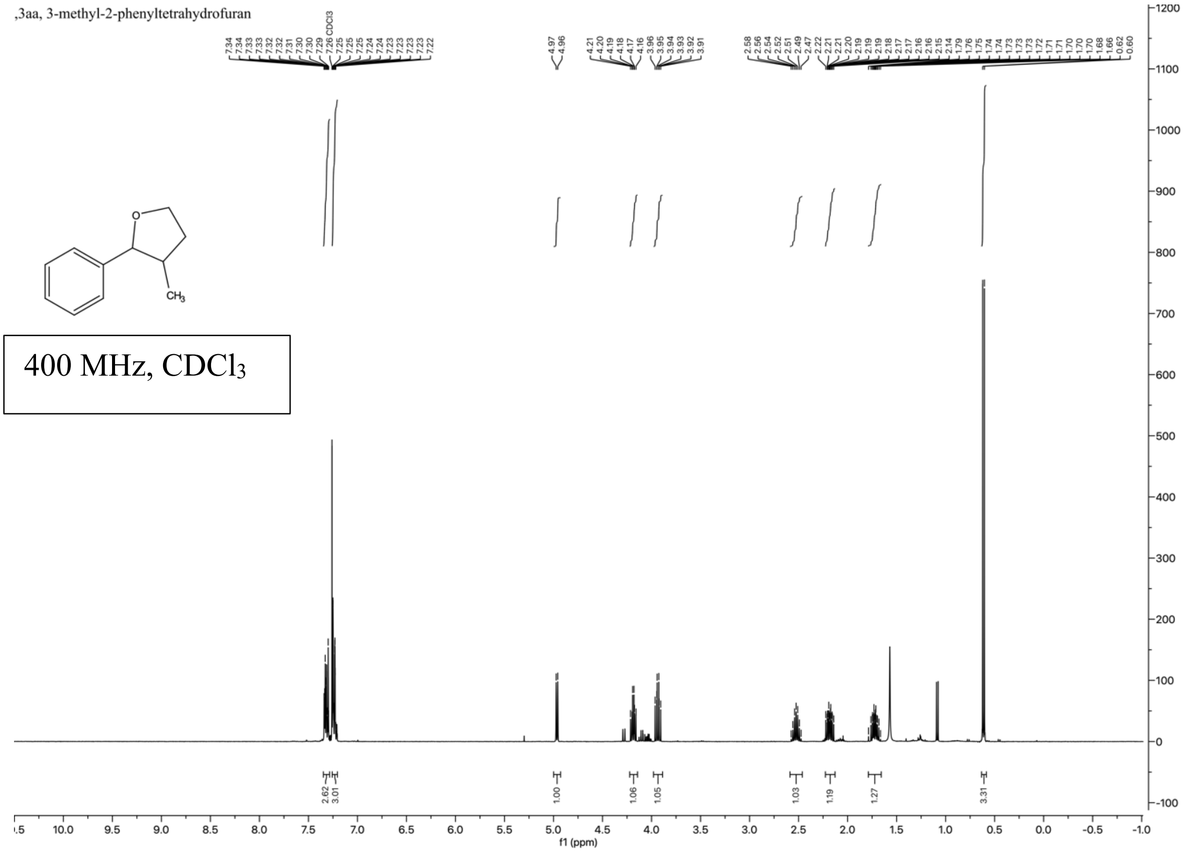
3aa, 3-methyl-2-phenyltetrahydrofuran
400 MHz, CDCl3
f1 (ppm)

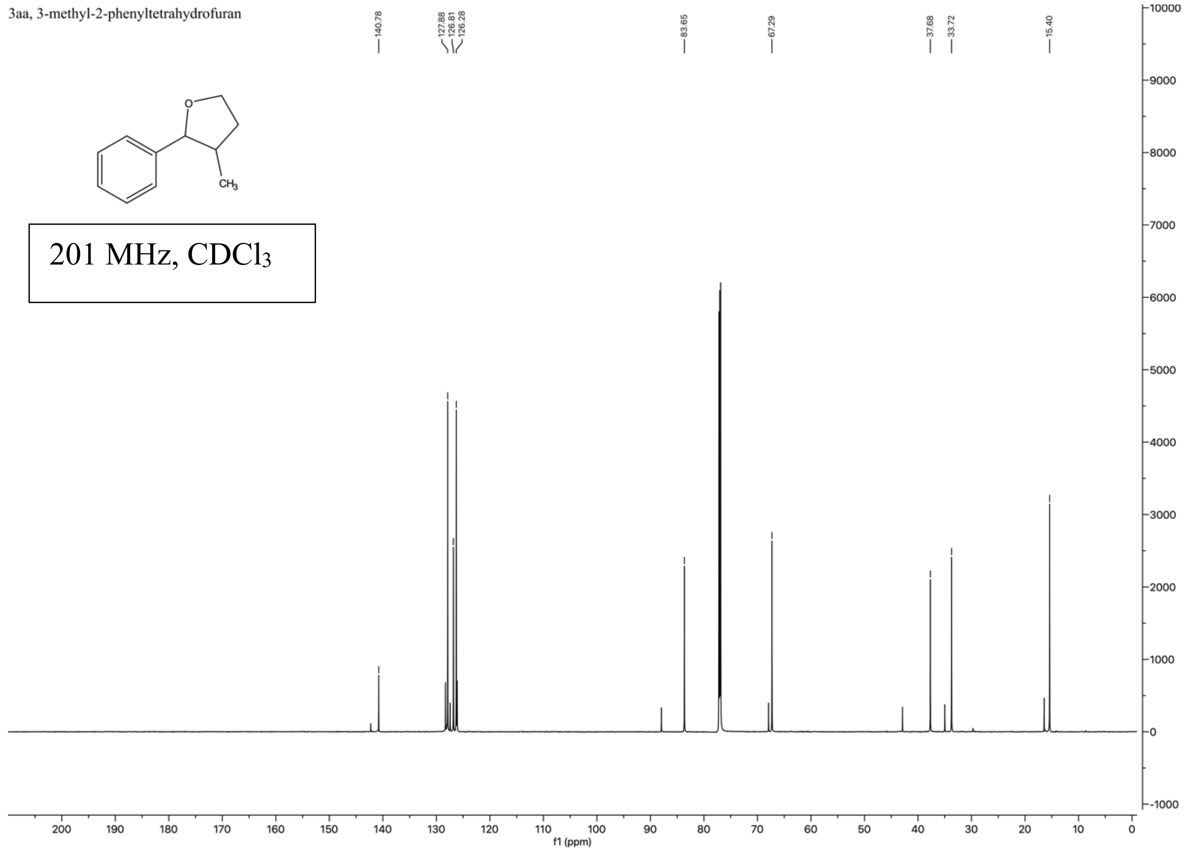
3aa, 3-methyl-2-phenyltetrahydrofuran
140.78
127.88
126.81
126.28
83.65
67.29
37.68
33.72
15.40
201 MHz, CDCl3
f1 (ppm)

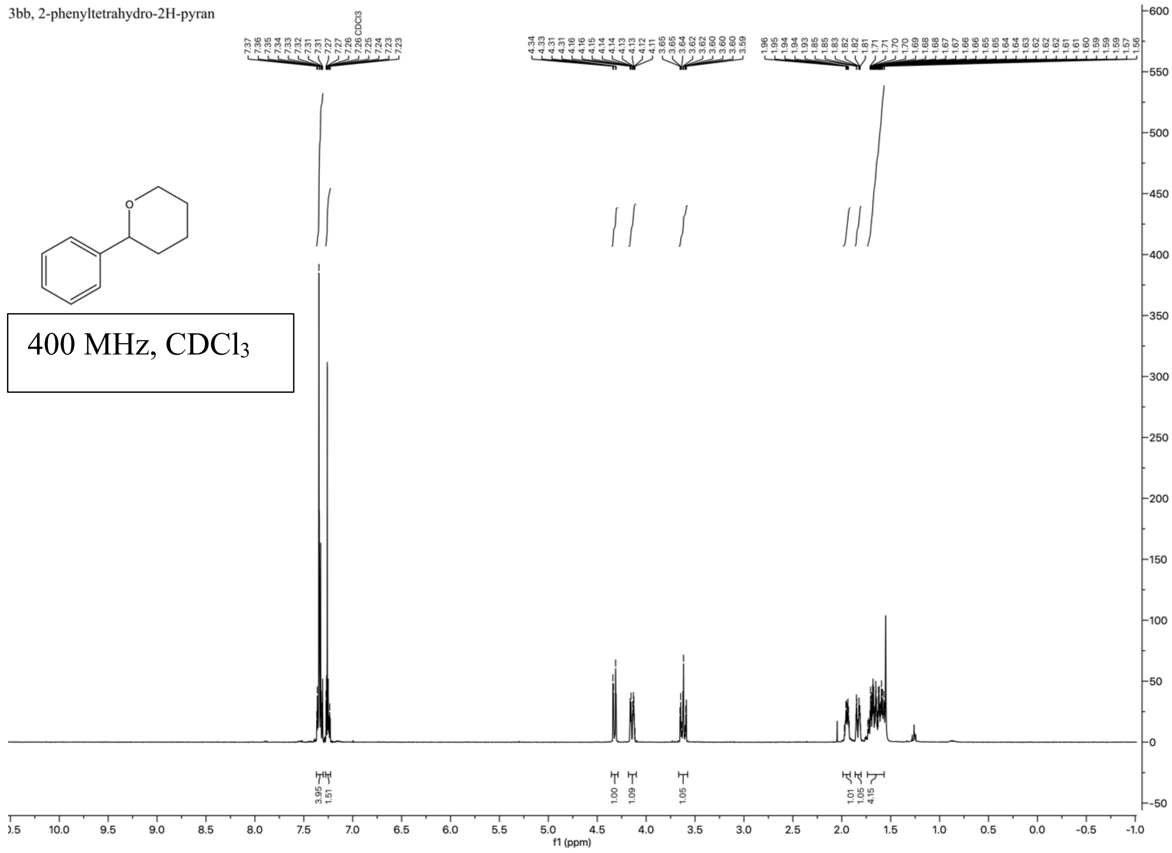
3bb, 2-phenyltetrahydro-2H-pyran
400 MHz, CDCl3
3.95
1.51
1.00
1.09
1.05
1.01
1.05
4.15
f1 (ppm)

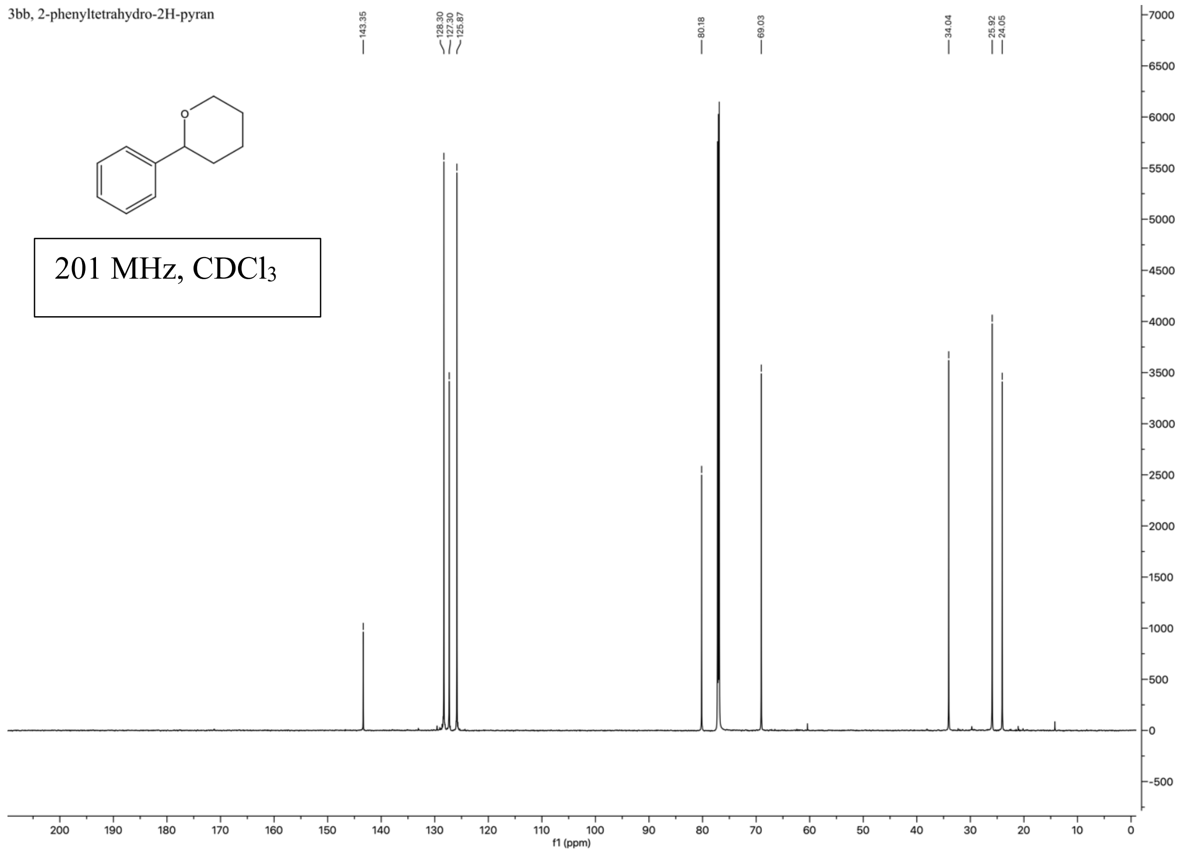
3bb, 2-phenyltetrahydro-2H-pyran
143.35
128.30
127.30
125.87
80.18
69.03
34.04
25.92
24.05
201 MHz, CDCl3
f1 (ppm)

3cc, 2-phenyl-1,4-dioxane

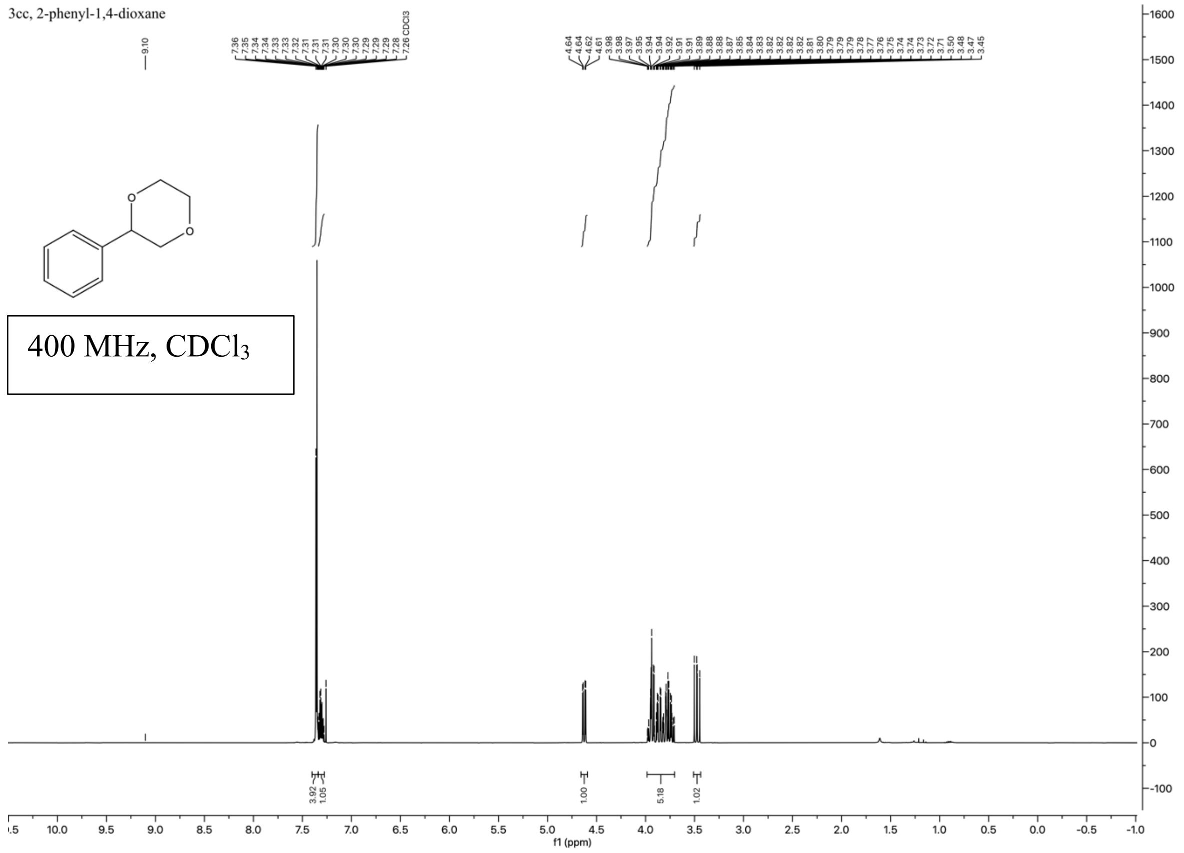

3cc, 2-phenyl-1,4-dioxane

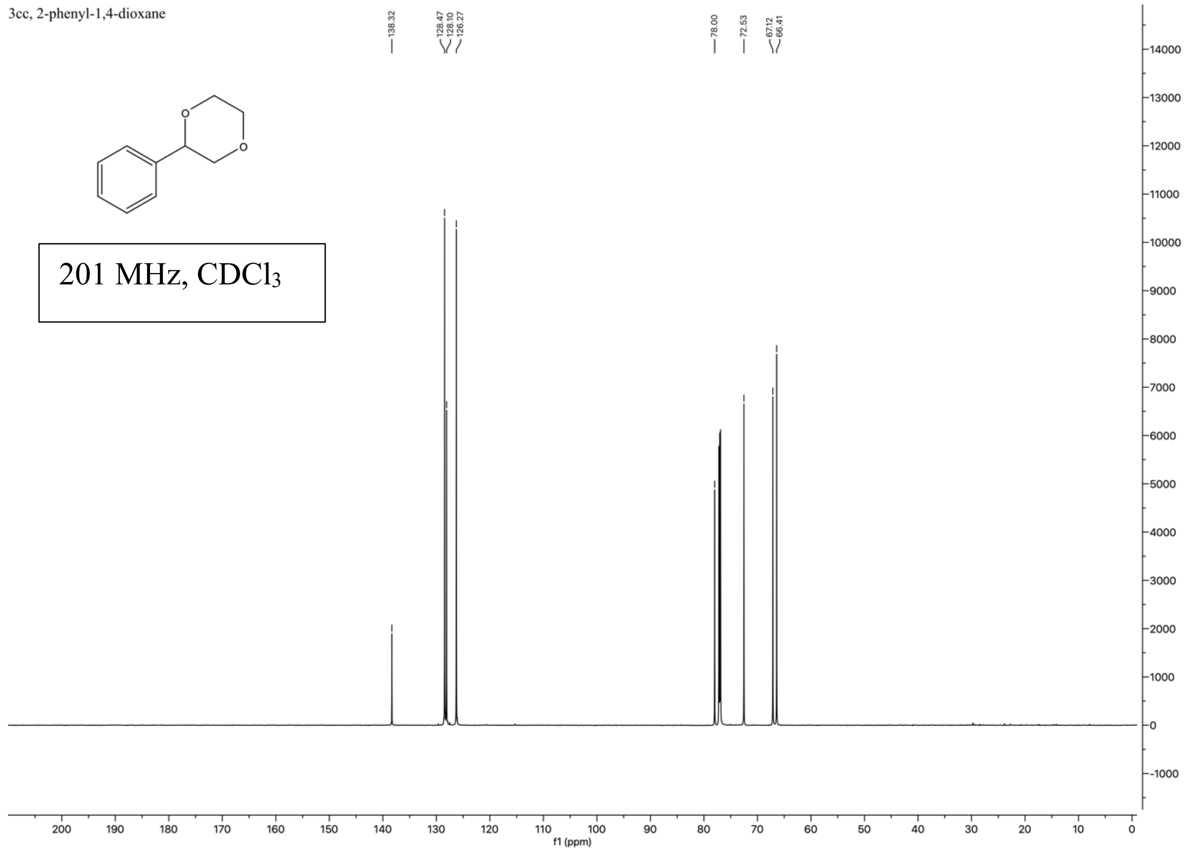

3gg, 2-(3-bromobenzo[b]thiophen-2-yl)-1,3-dioxolane

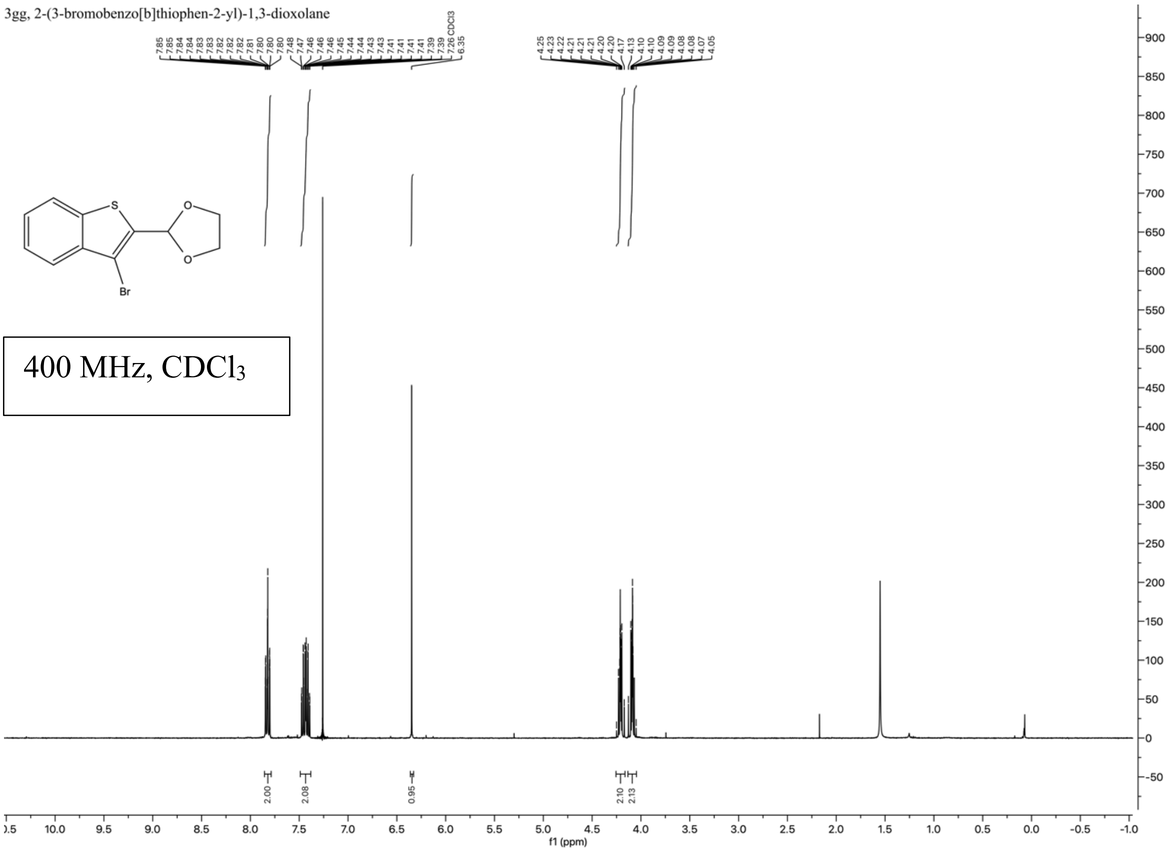

3gg, 2-(3-bromobenzo[b]thiophen-2-yl)-1,3-dioxolane

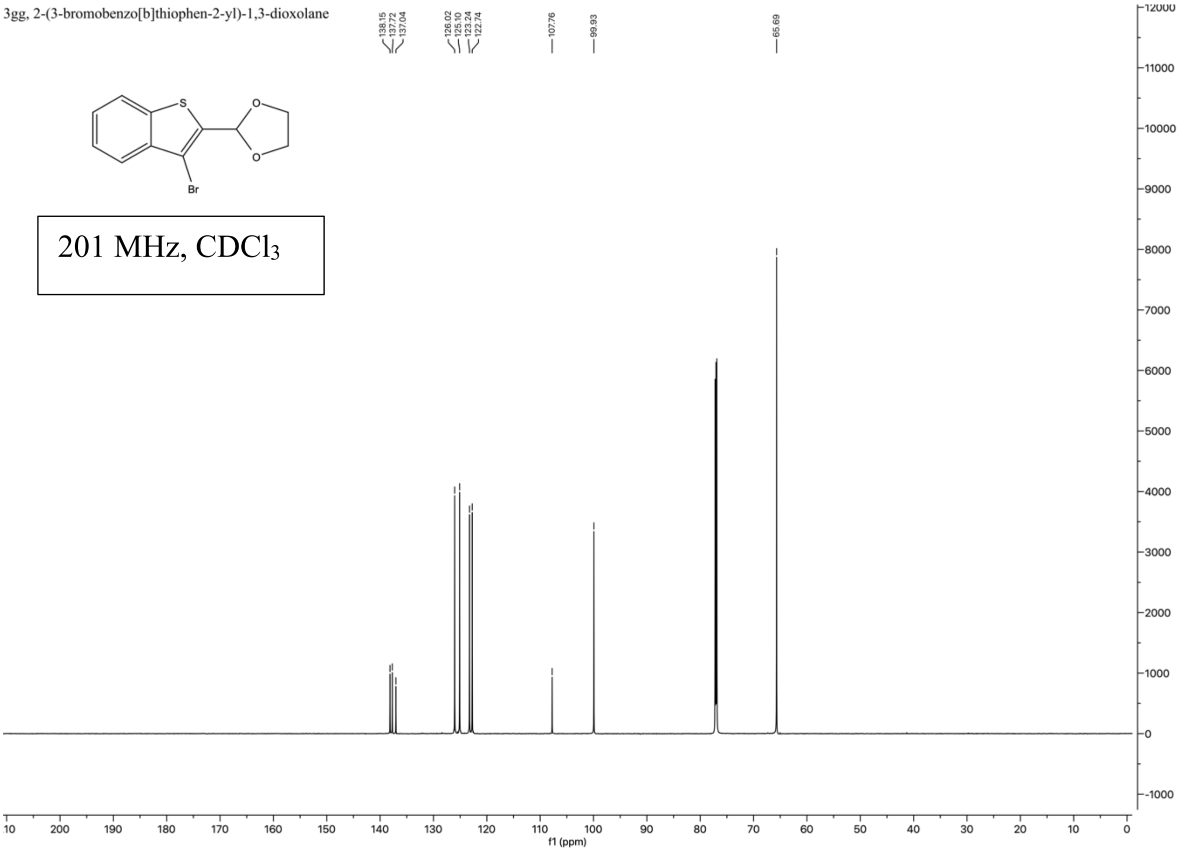

3hh, 2-(1,3-dioxolan-2-yl)benzofuran

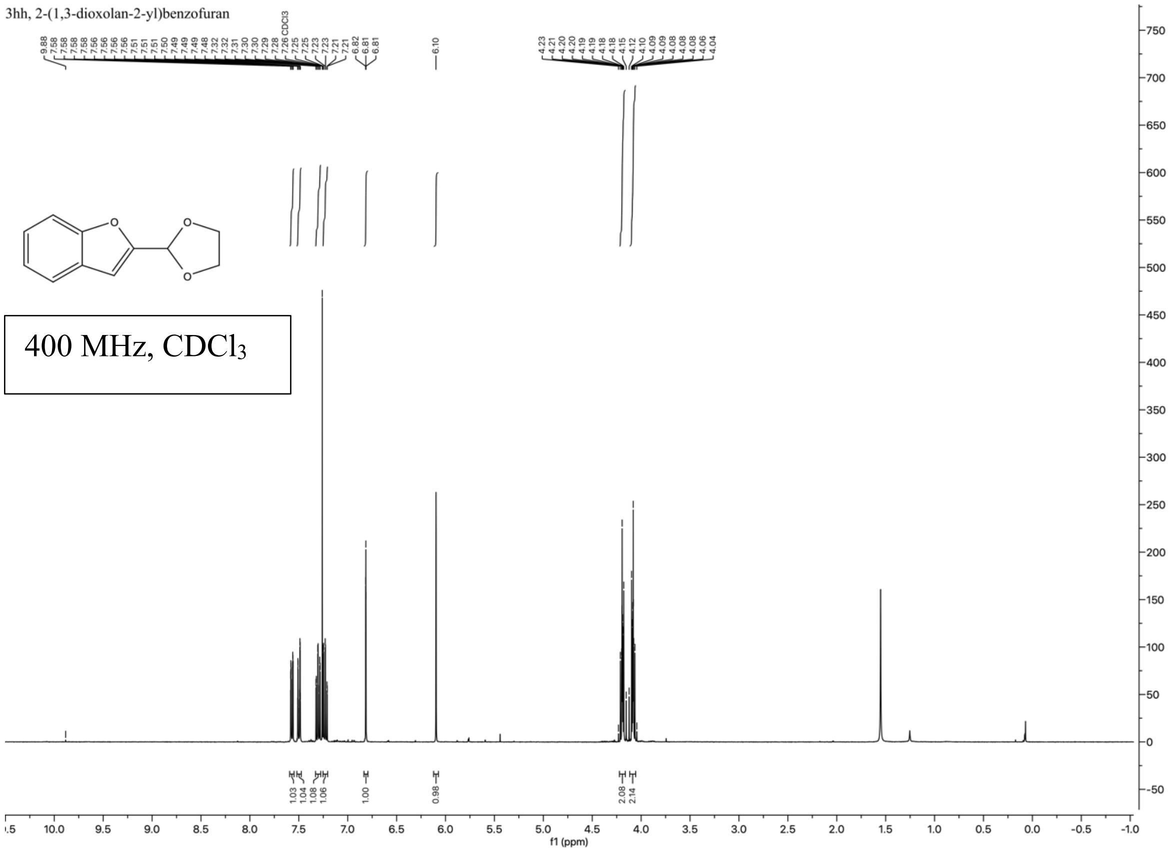

3hh, 2-(1,3-dioxolan-2-yl)benzofuran

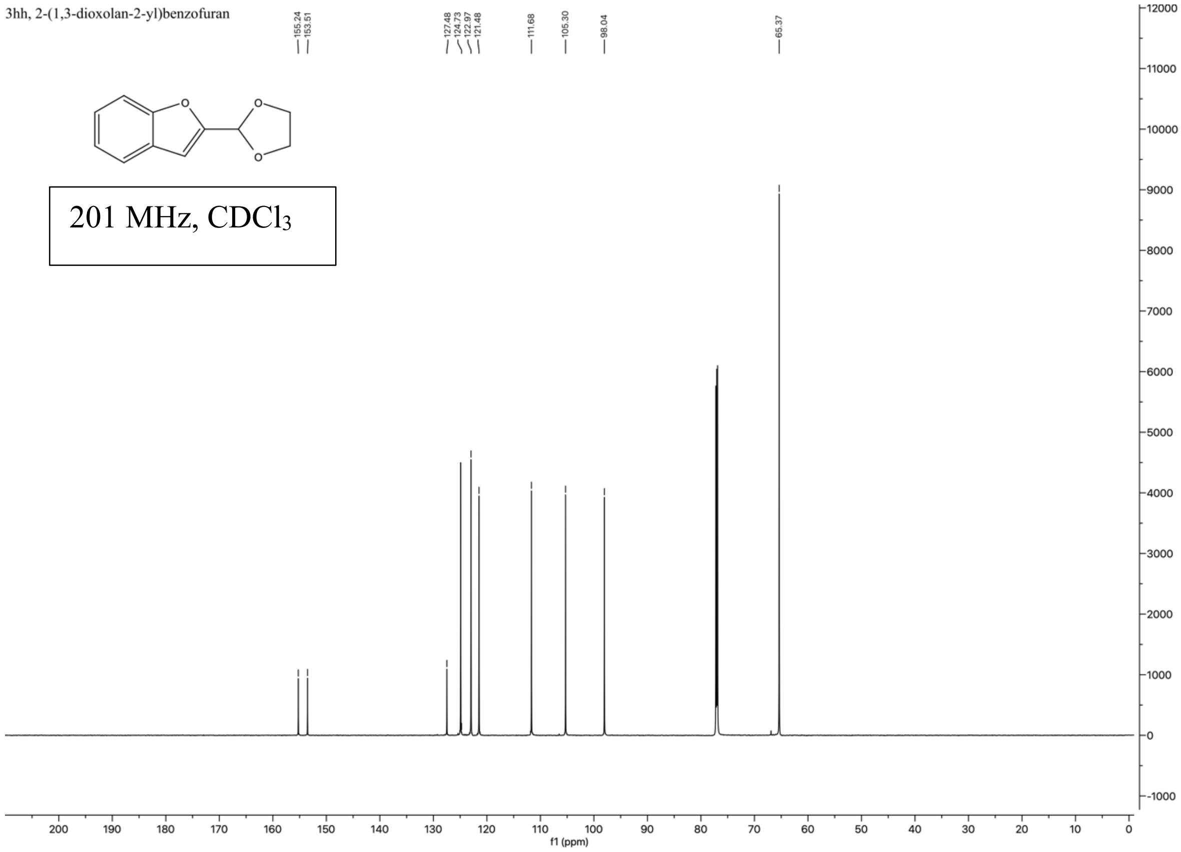

3ii, 2-octyltetrahydrofuran

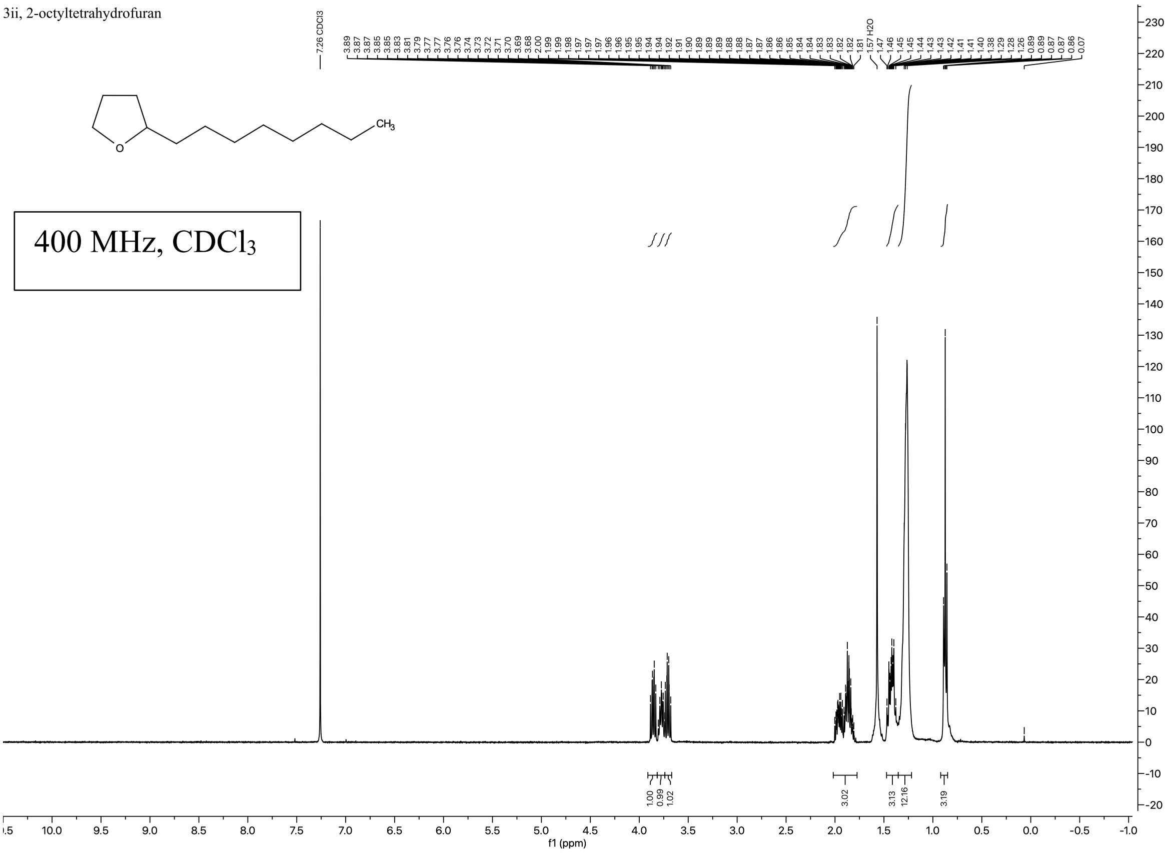

3ii, 2-octyltetrahydrofuran

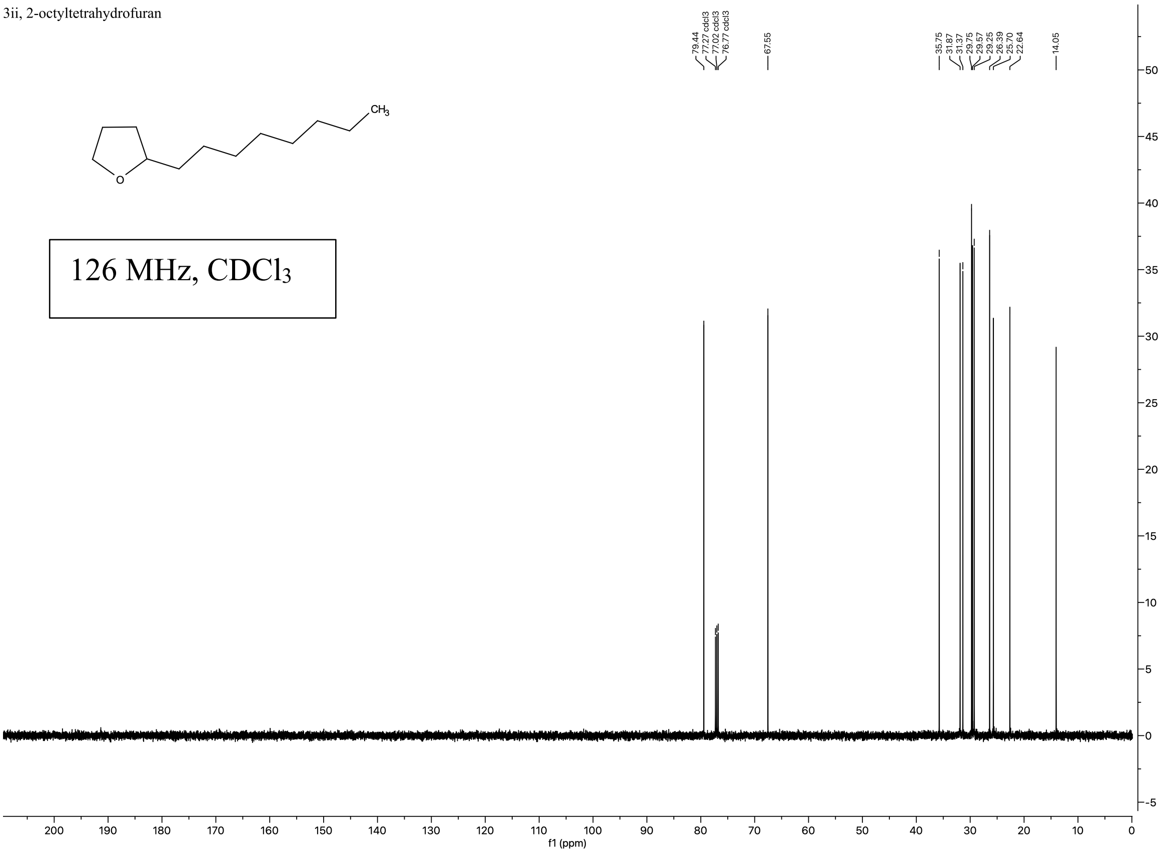

3jj(a) Benzylmethyl ether

400 MHz, $CDCl_3$

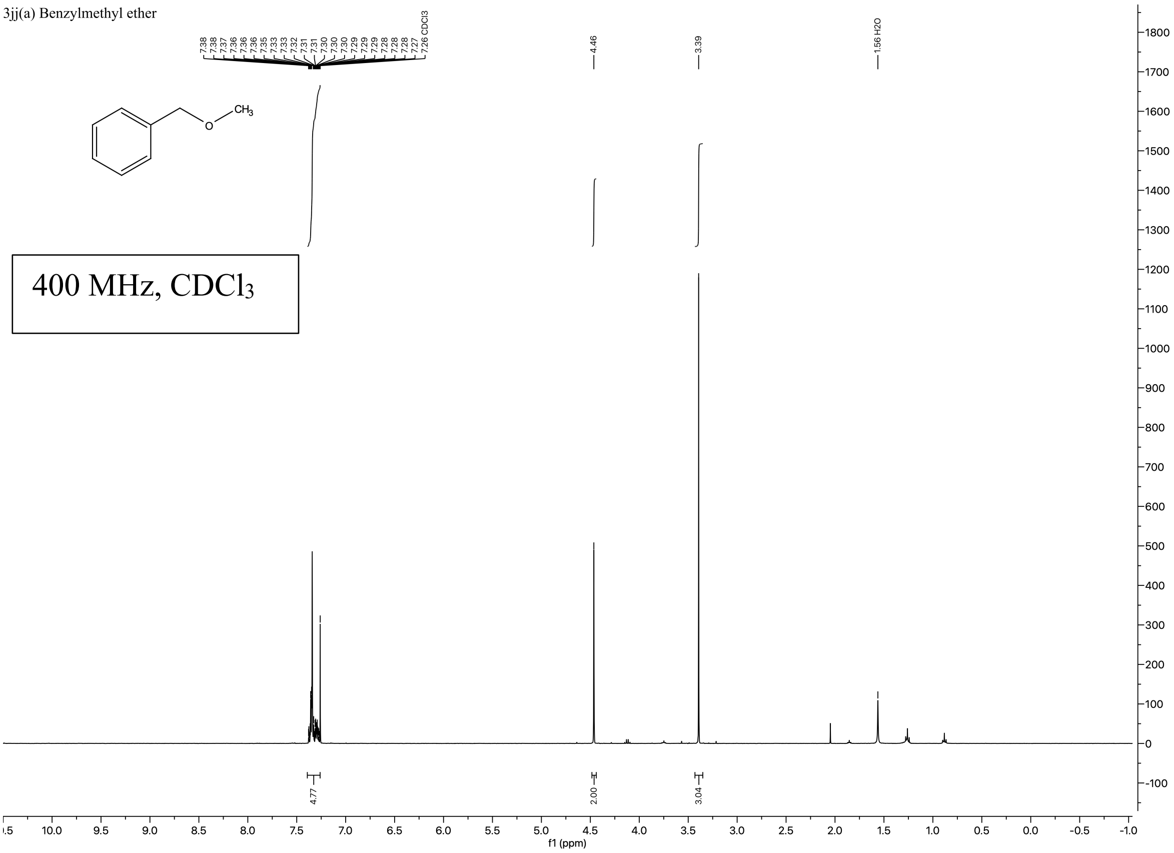

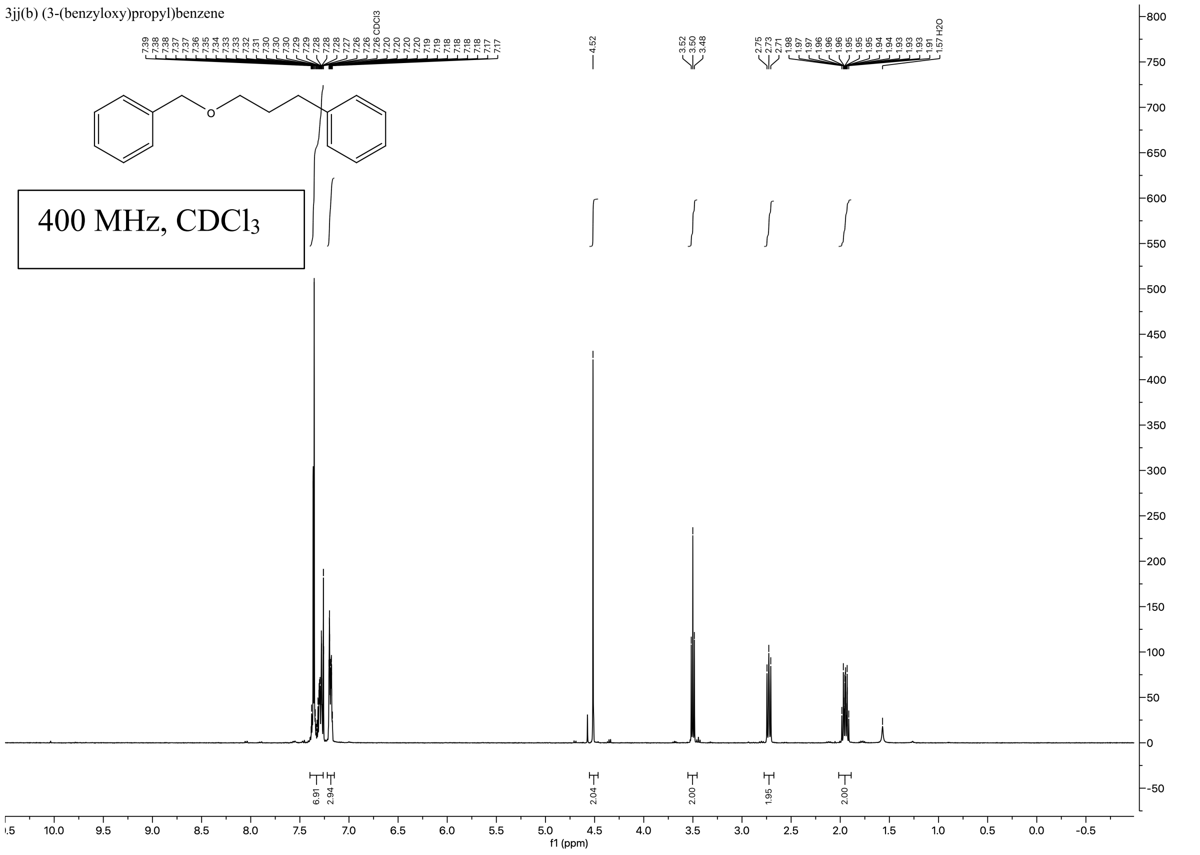
3jj(b) (3-(benzyloxy)propyl)benzene
400 MHz, CDCl3
f1 (ppm)

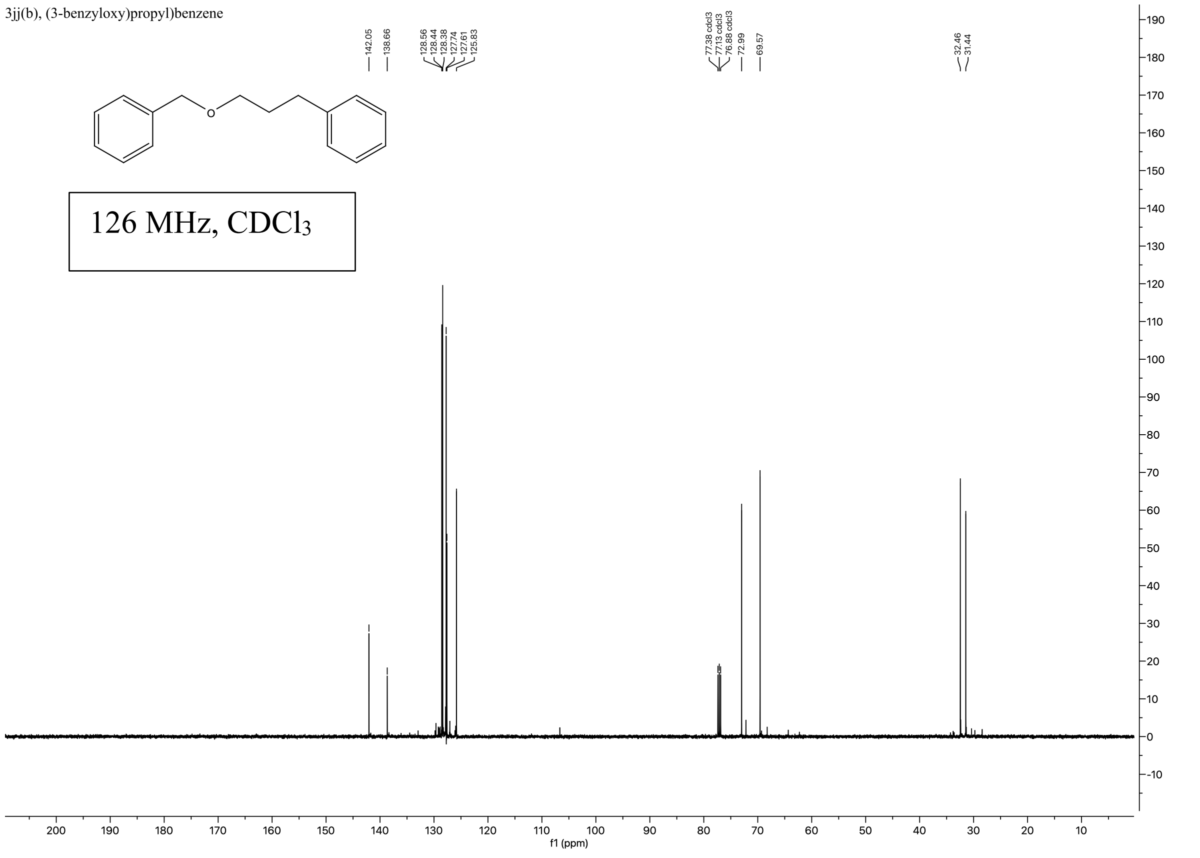
3jj(b), (3-benzyloxy)propyl)benzene
126 MHz, CDCl3
f1 (ppm)

3kk, 1-(1-methoxyethyl)-4-(trifluoromethyl)benzene

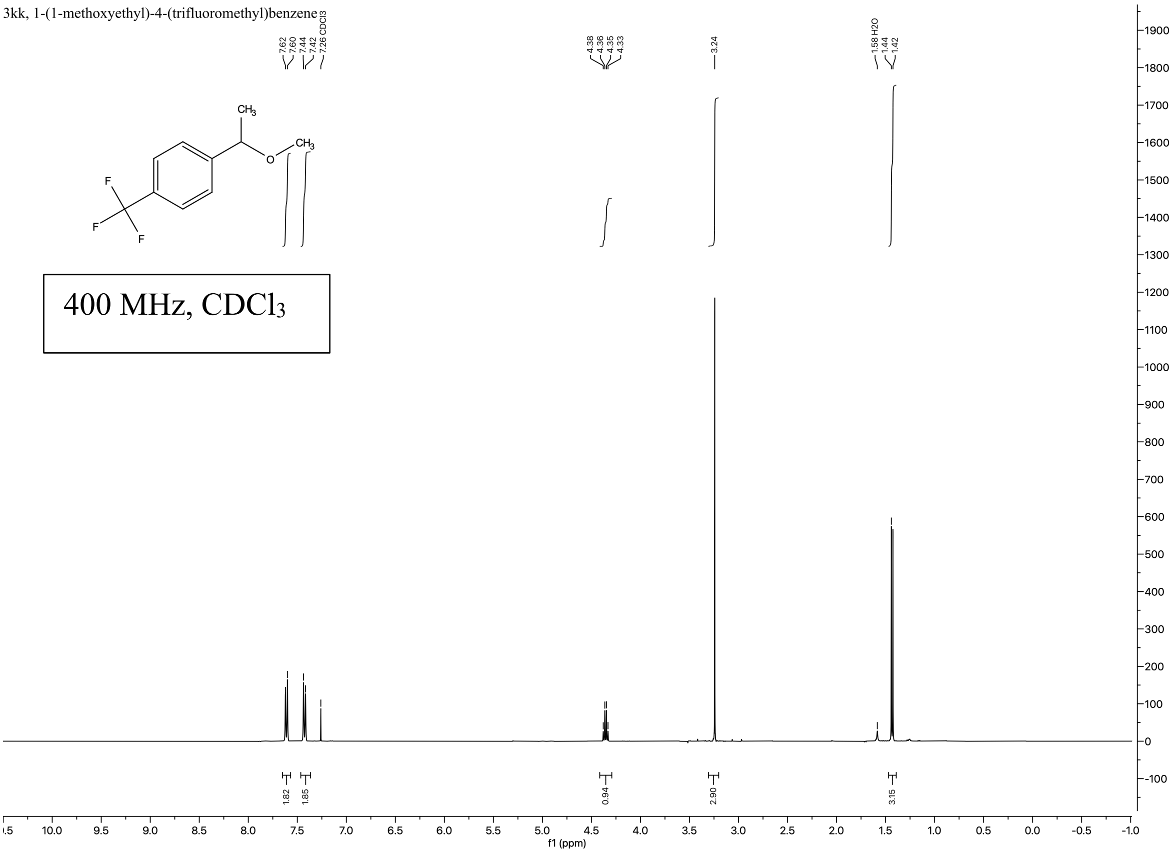

3kk, 1-(1-methoxyethyl)-4-(trifluoromethyl)benzene

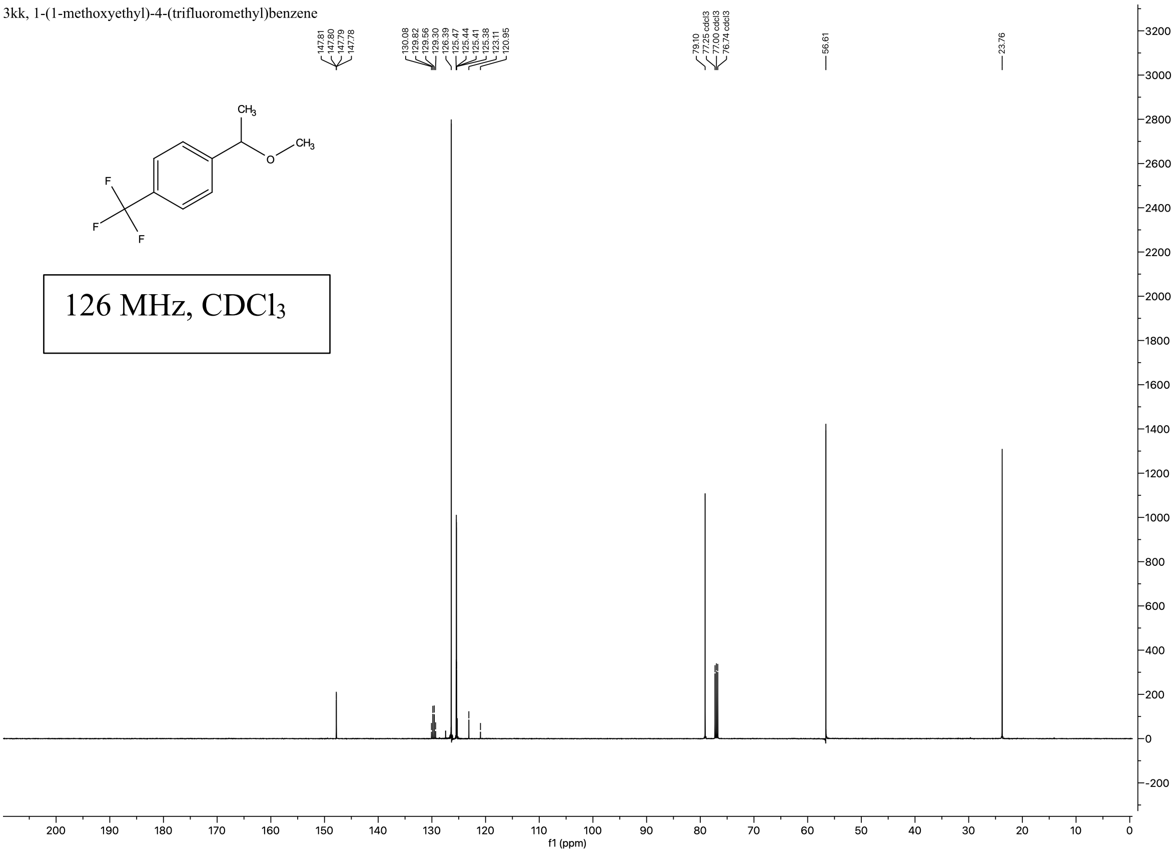

4aa, 2-methyl-2-(4-(tetrahydrofuran-2-yl)phenyl)propanenitrile

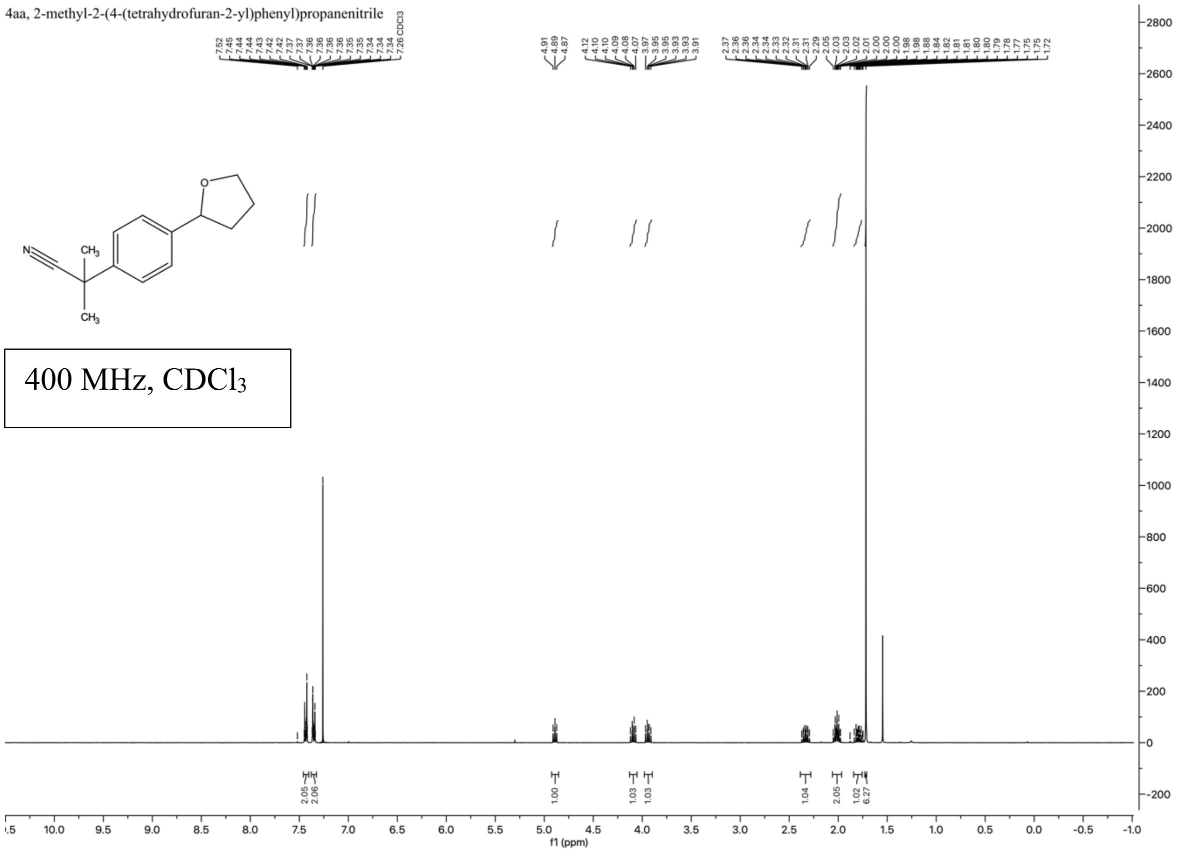

4aa, 2-methyl-2-(4-(tetrahydrofuran-2-yl)phenyl)propanenitrile

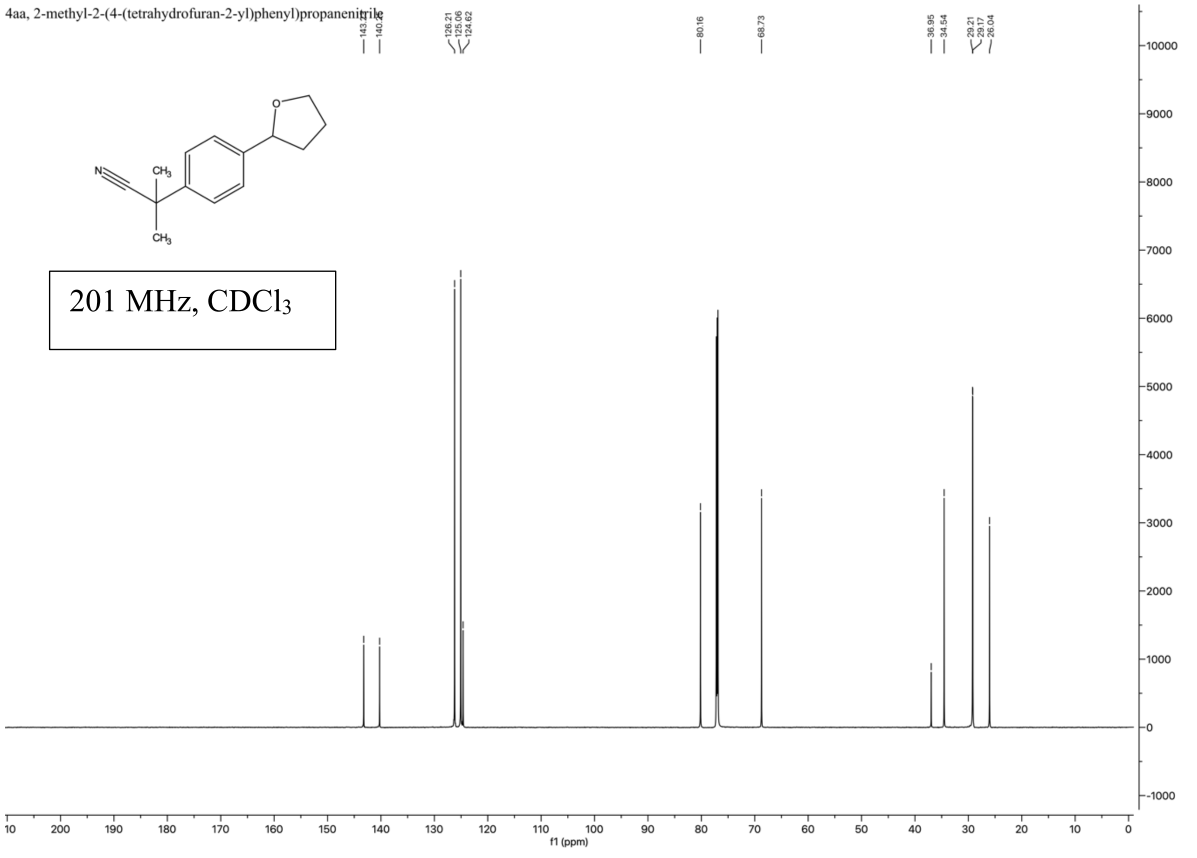

1a, 4-bromo-1-(4-chlorophenyl)butan-1-one

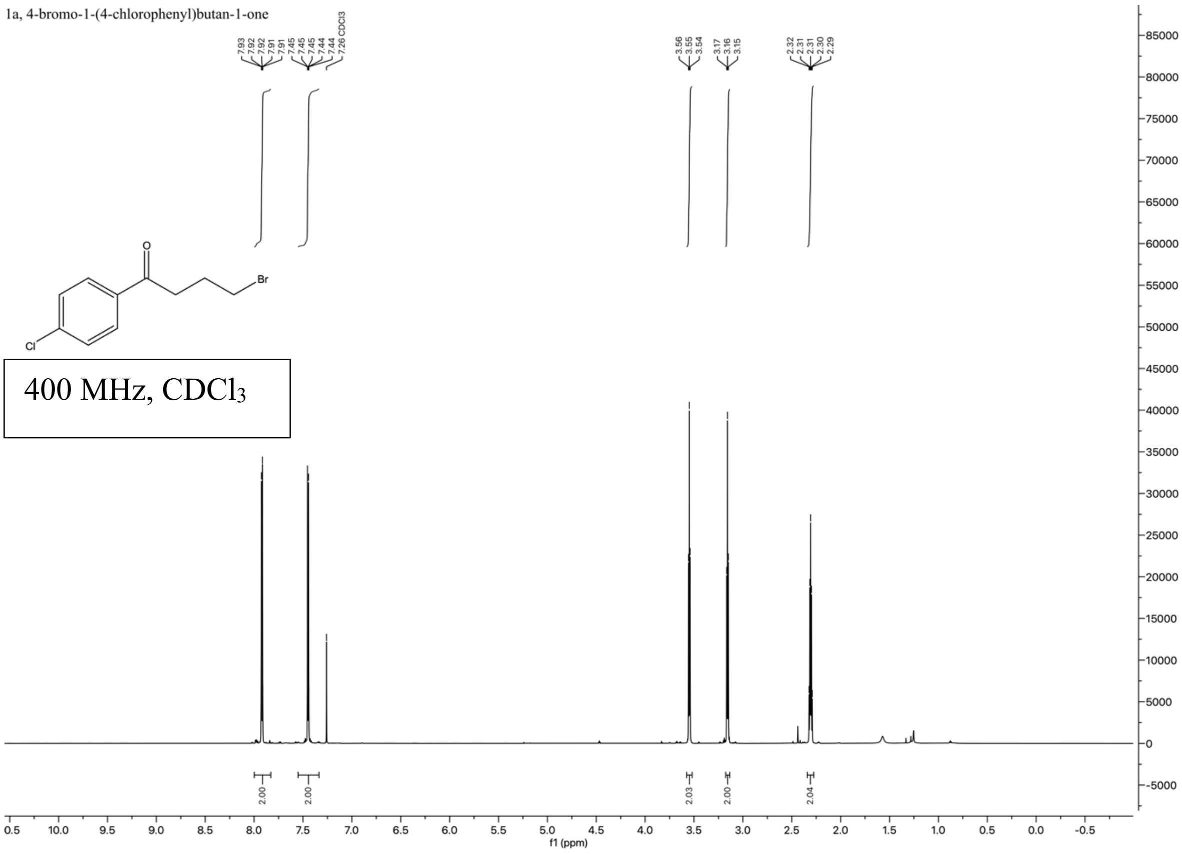

1a, 4-bromo-1-(4-chlorophenyl)butan-1-one

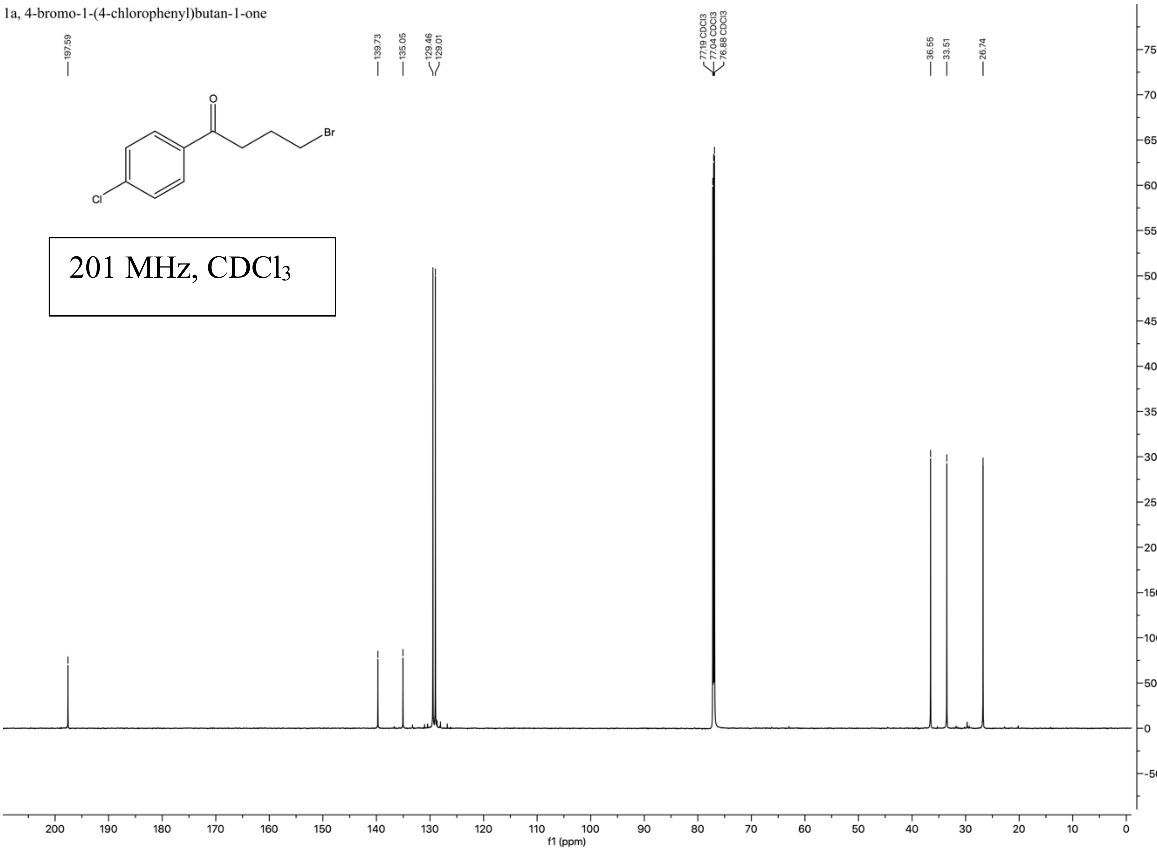

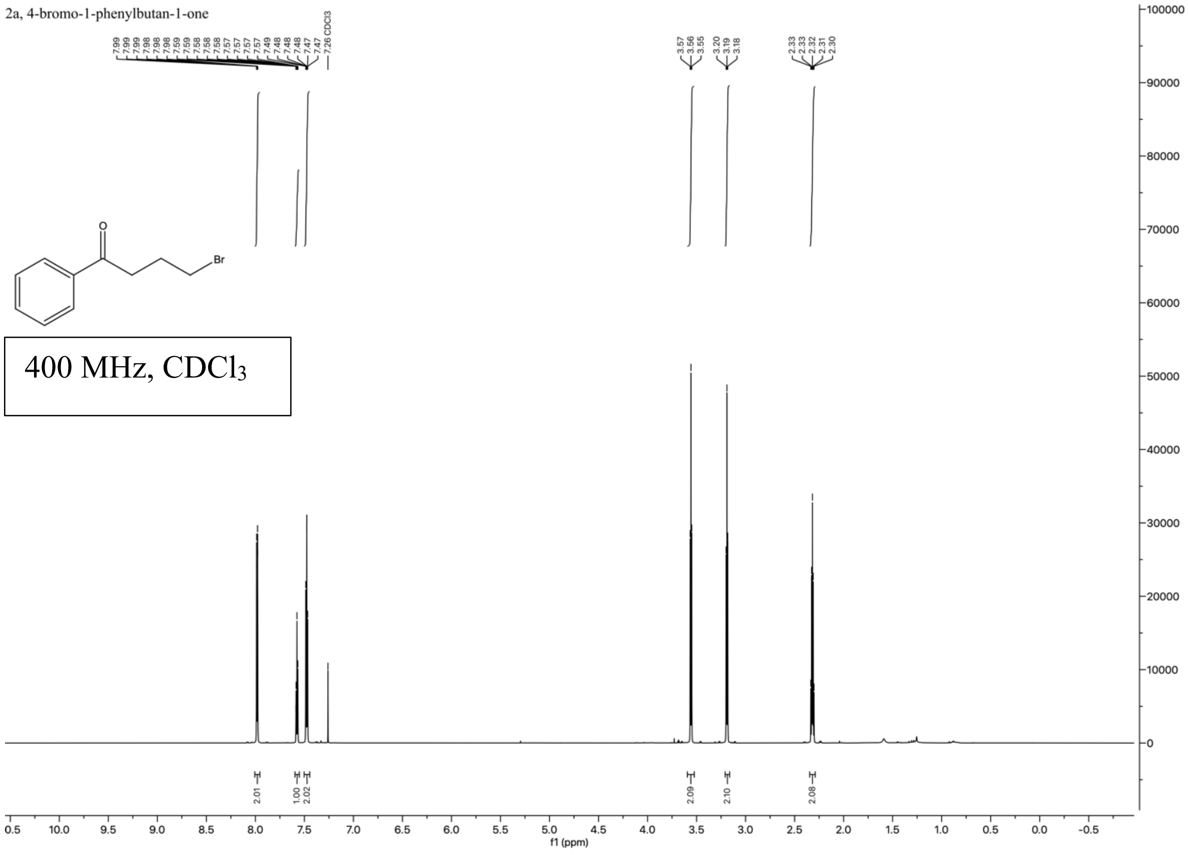
2a, 4-bromo-1-phenylbutan-1-one
Br
O
400 MHz, $CDCl_3$
7.99
7.99
7.99
7.98
7.98
7.98
7.59
7.58
7.58
7.58
7.57
7.57
7.57
7.49
7.48
7.48
7.48
7.47
7.47
7.26 CDCl3
3.57
3.56
3.55
3.20
3.19
3.18
2.33
2.33
2.32
2.31
2.30
2.01
1.00
2.02
2.09
2.10
2.08
f1 (ppm)

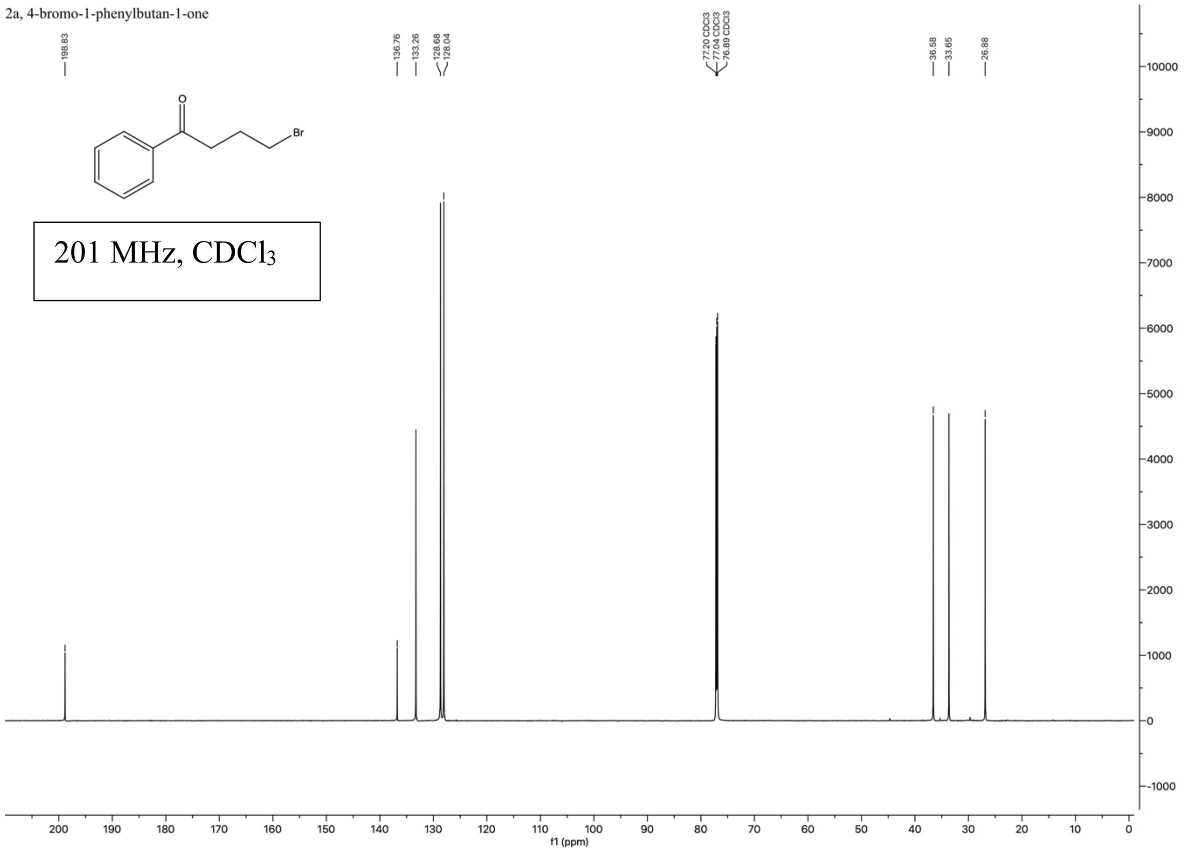
2a, 4-bromo-1-phenylbutan-1-one
Br
O
201 MHz, $CDCl_3$
198.83
136.76
133.26
128.68
128.04
77.20 CDCl3
77.04 CDCl3
76.89 CDCl3
36.58
33.65
26.88
f1 (ppm)

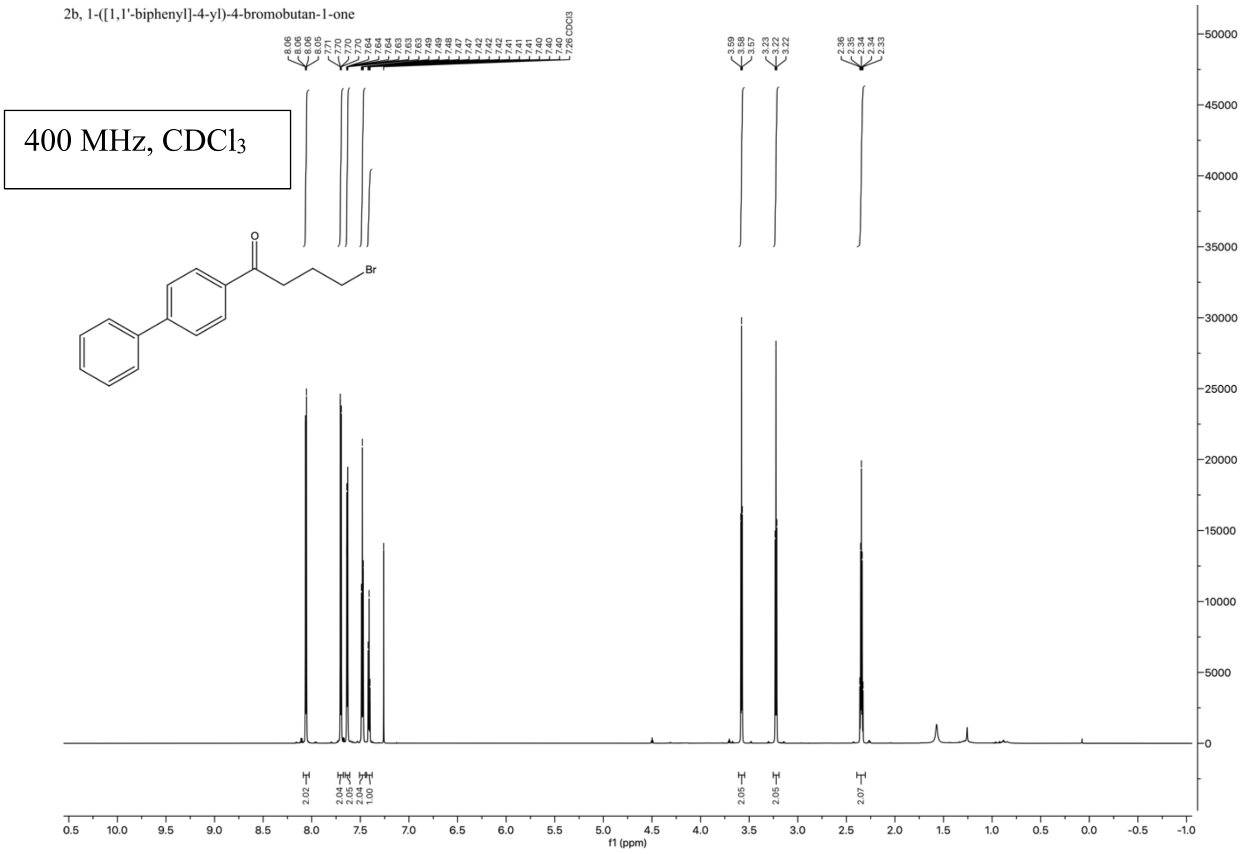
2b, 1-([1,1'-biphenyl]-4-yl)-4-bromobutan-1-one
400 MHz, CDCl3
O
Br
f1 (ppm)

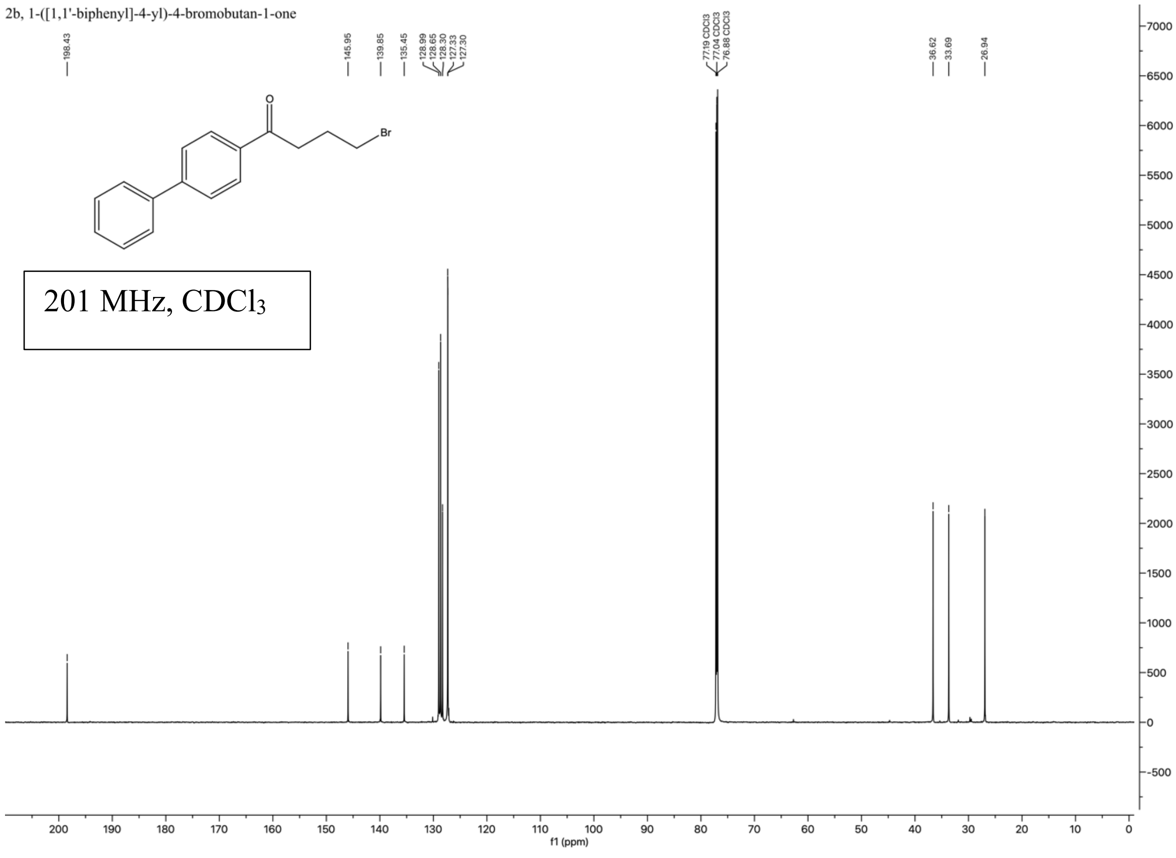
2b, 1-([1,1'-biphenyl]-4-yl)-4-bromobutan-1-one
201 MHz, CDCl3
O
Br
f1 (ppm)

2c, 4-(4-bromobutanoyl)benzonitrile

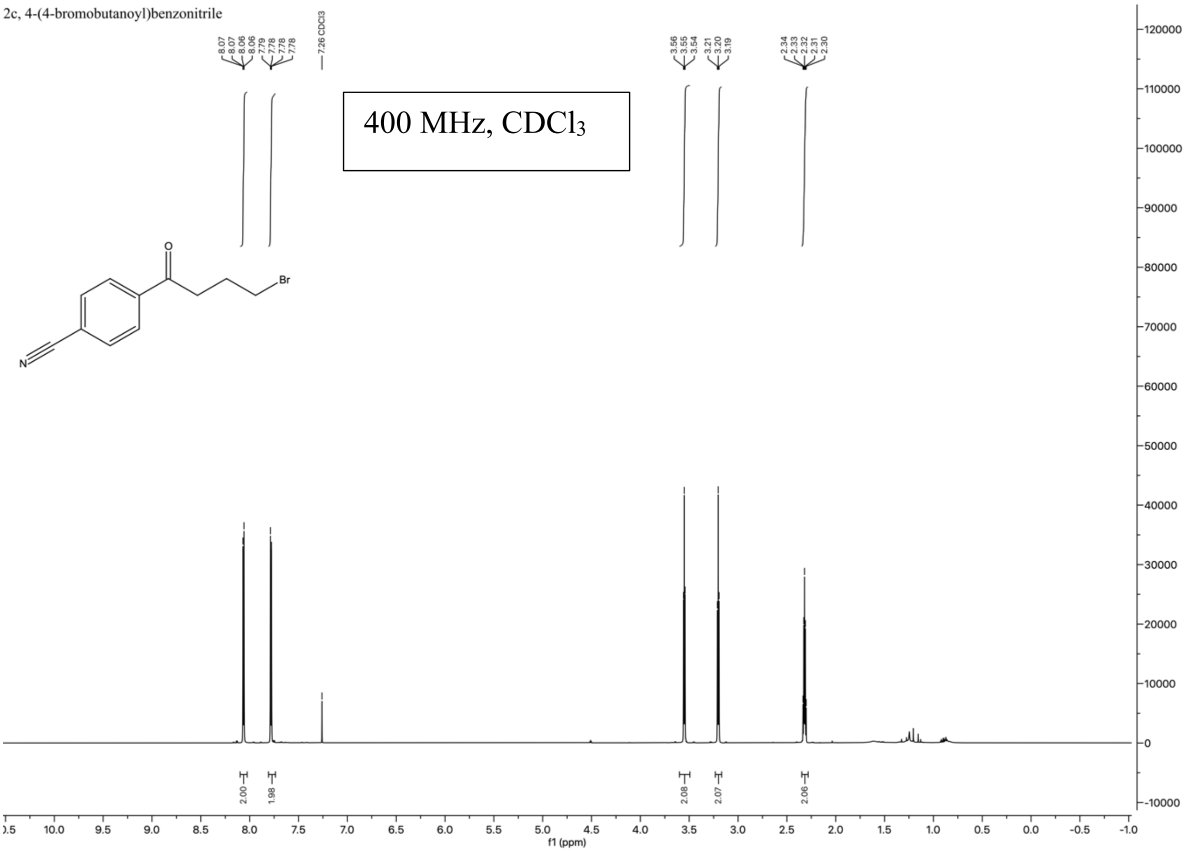

2c, 4-(4-bromobutanoyl)benzonitrile

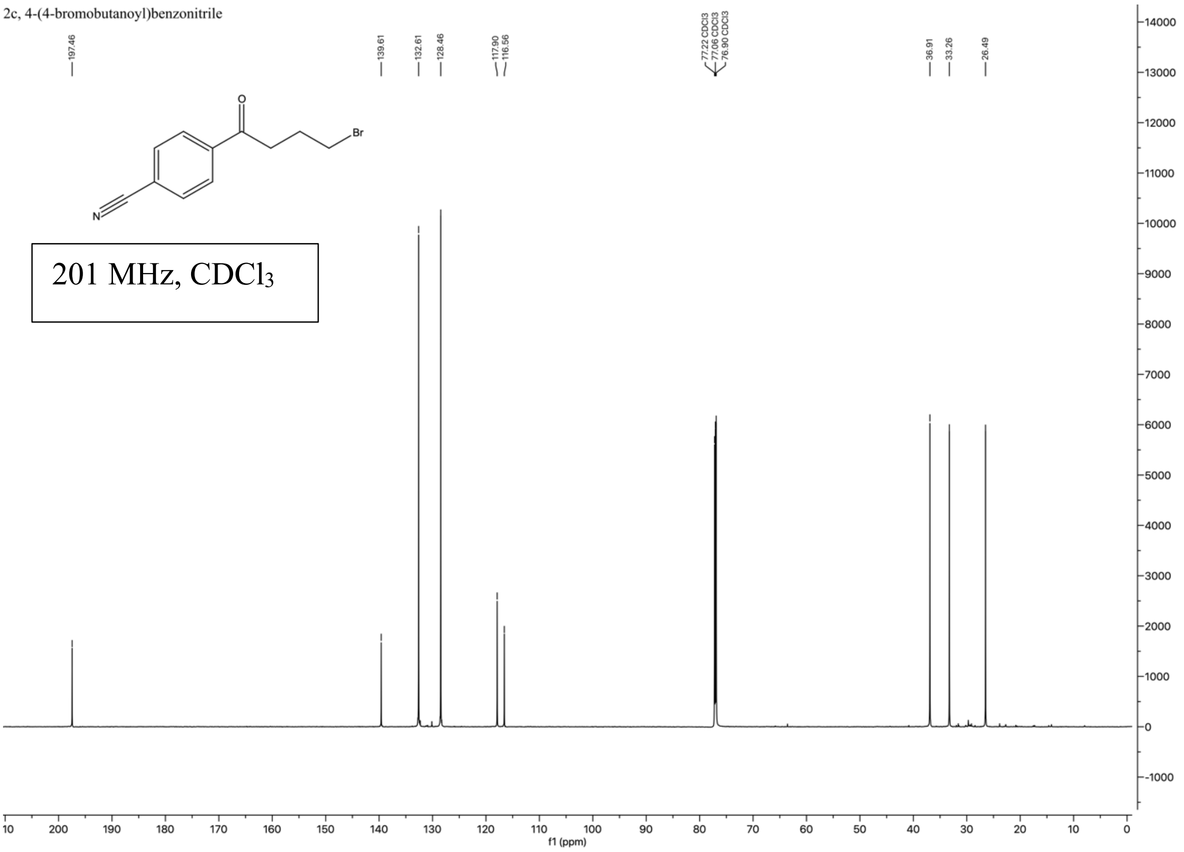

2d, 4-bromo-1-(4-methoxyphenyl)butan-1-one

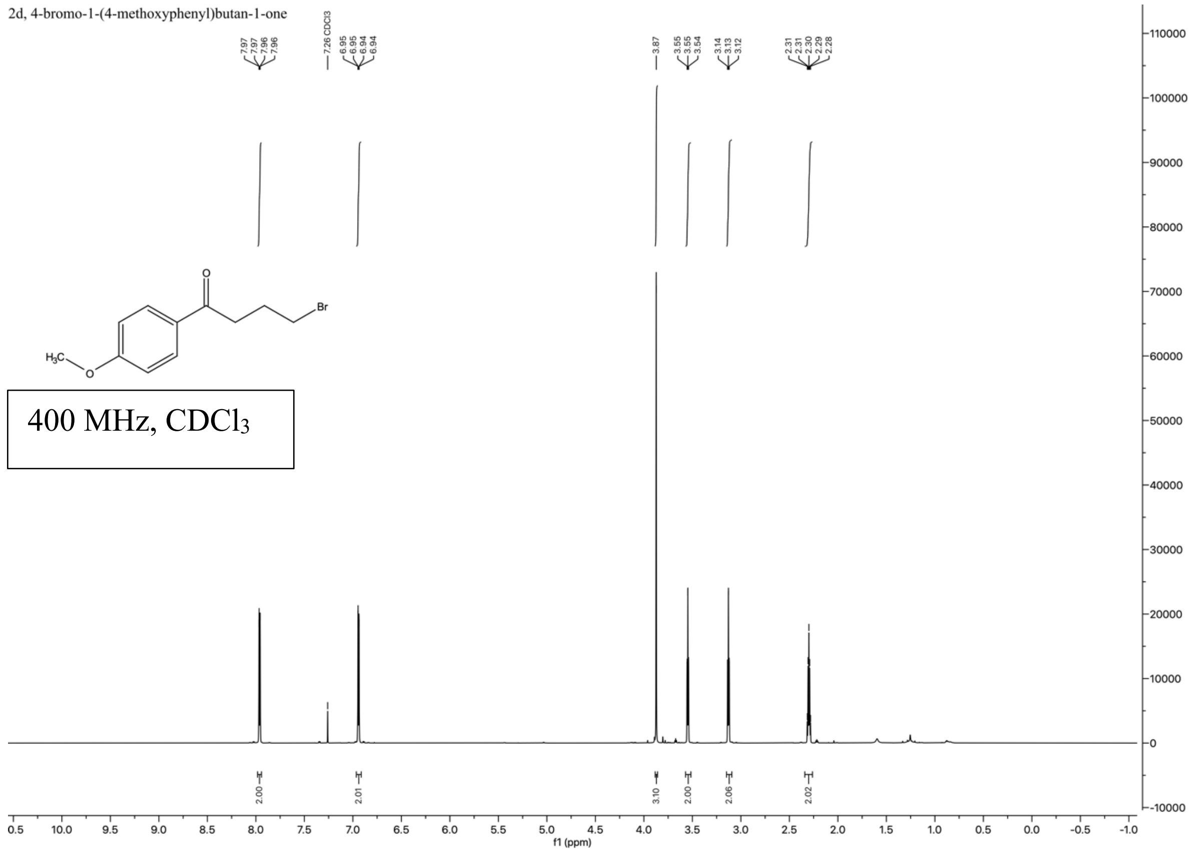

2d, 4-bromo-1-(4-methoxyphenyl)butan-1-one

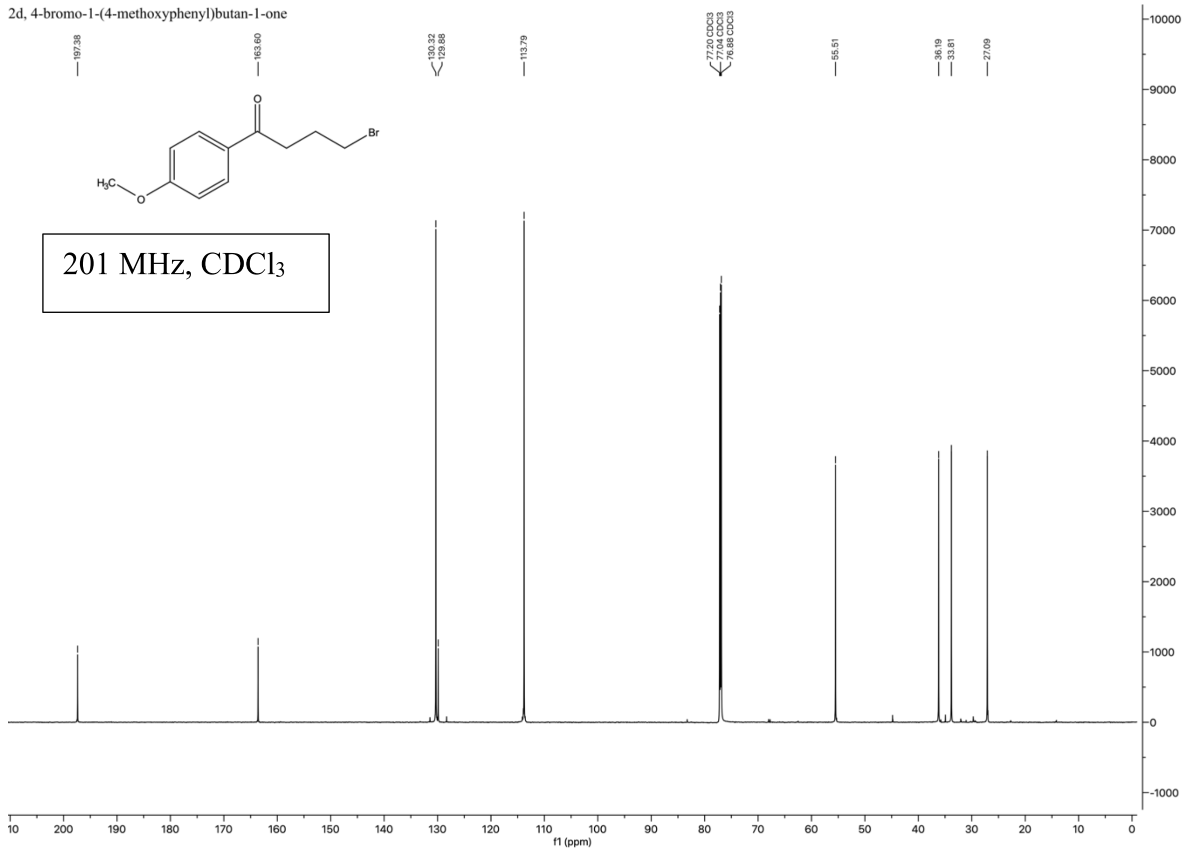

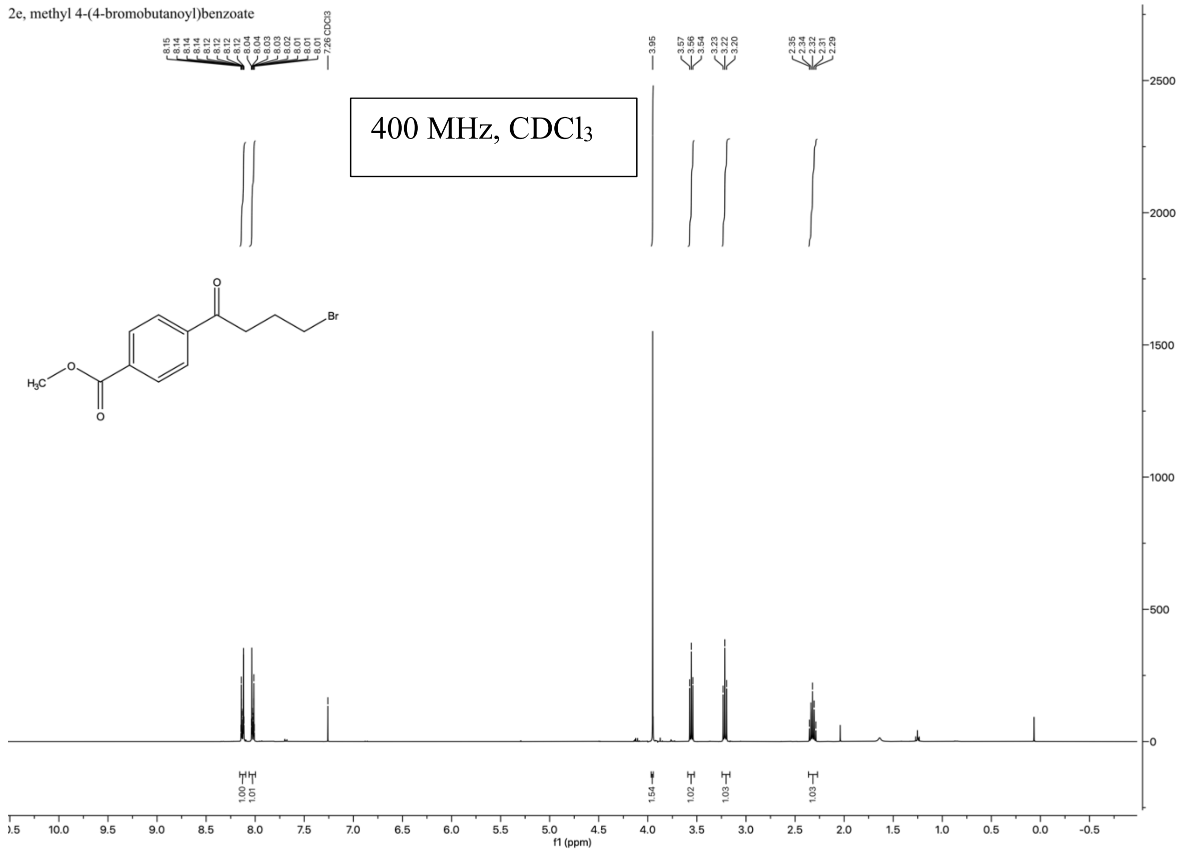
2e, methyl 4-(4-bromobutanoyl)benzoate
8.15
8.14
8.14
8.12
8.12
8.12
8.04
8.04
8.03
8.02
8.01
8.01
7.26 CDCl3
3.95
3.57
3.56
3.54
3.23
3.22
3.20
2.35
2.34
2.32
2.31
2.29
400 MHz, CDCl3
1.00
1.01
1.54
1.02
1.03
1.03
f1 (ppm)

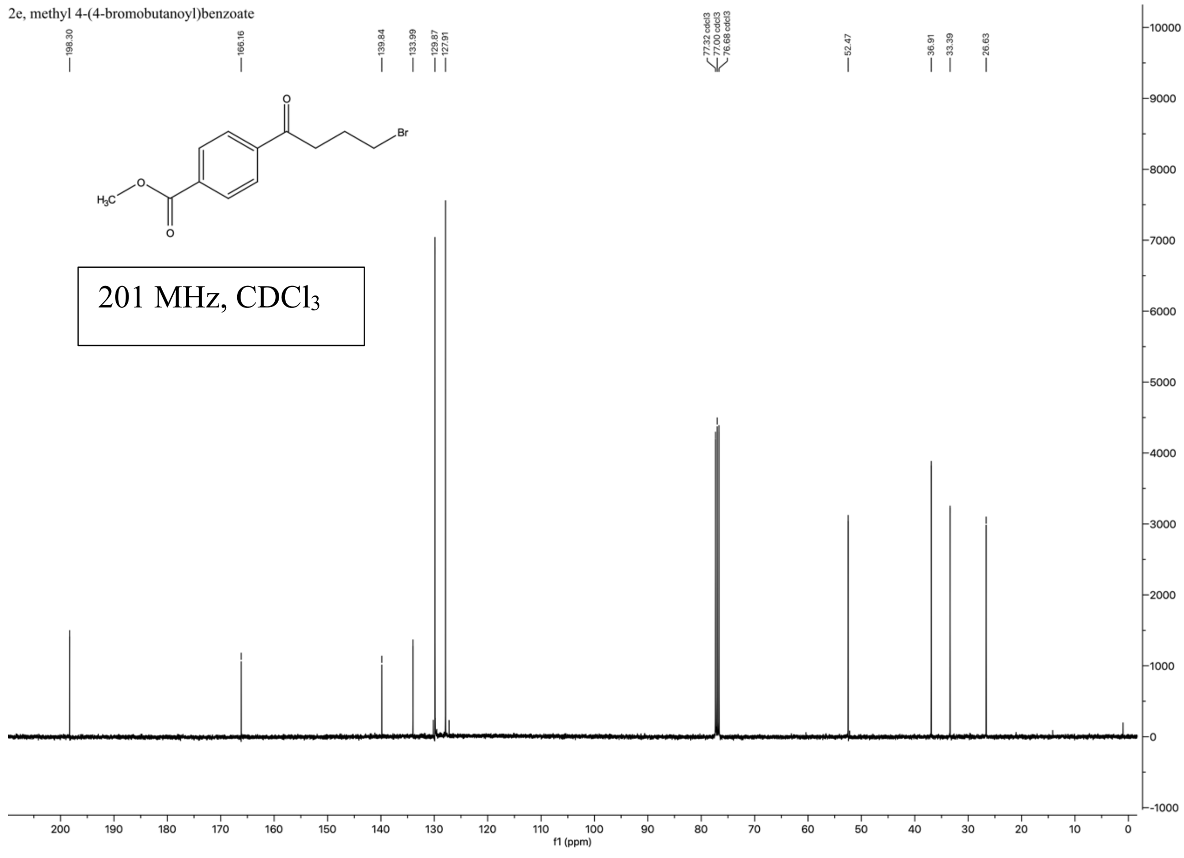
2e, methyl 4-(4-bromobutanoyl)benzoate
198.30
166.16
139.84
133.99
129.87
127.91
77.32 cdcl3
77.00 cdcl3
76.68 cdcl3
52.47
36.91
33.39
26.63
201 MHz, CDCl3
f1 (ppm)

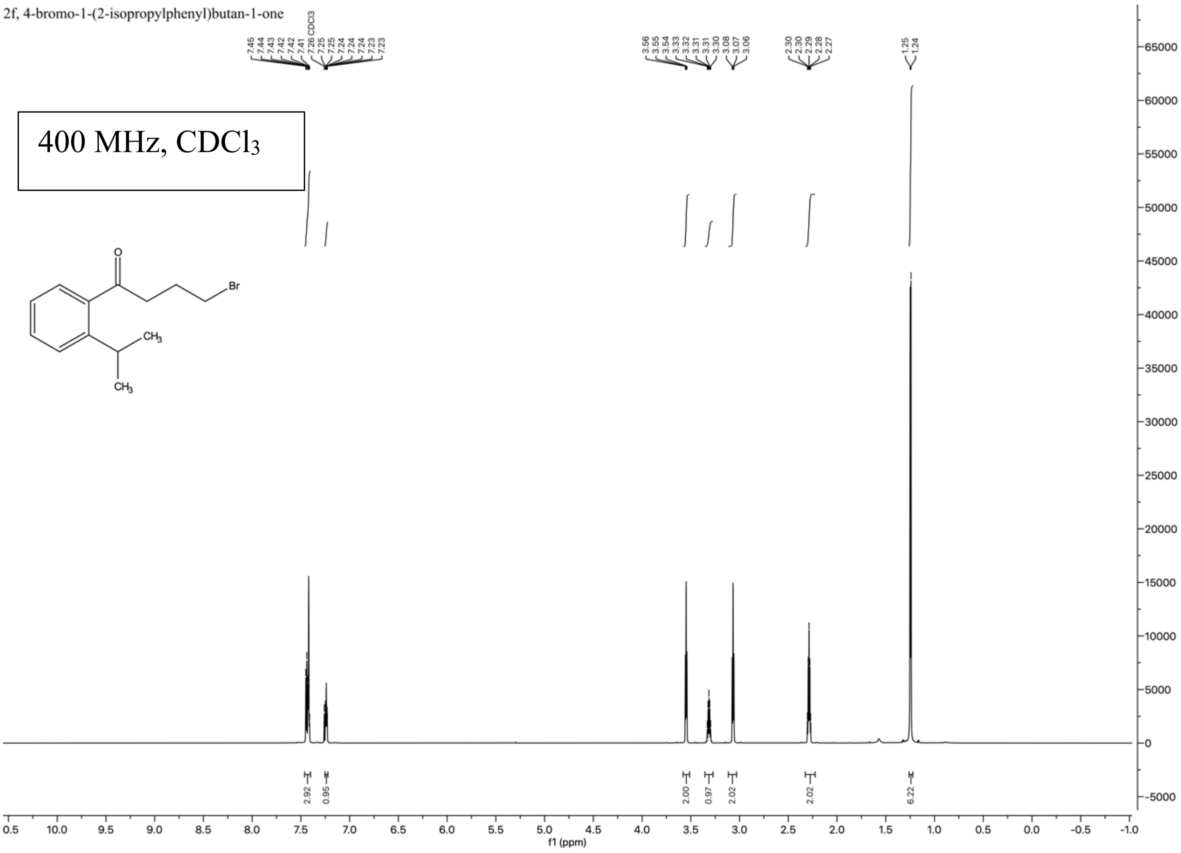
2f, 4-bromo-1-(2-isopropylphenyl)butan-1-one
400 MHz, CDCl3
7.45 7.44 7.43 7.42 7.42 7.41 7.26 CDCl3 7.25 7.25 7.24 7.24 7.24 7.23 7.23
3.56 3.55 3.54 3.33 3.32 3.31 3.31 3.30 3.08 3.07 3.06
2.30 2.30 2.29 2.28 2.27
1.25 1.24
2.92 0.95 2.00 0.97 2.02 2.02 6.22
f1 (ppm)

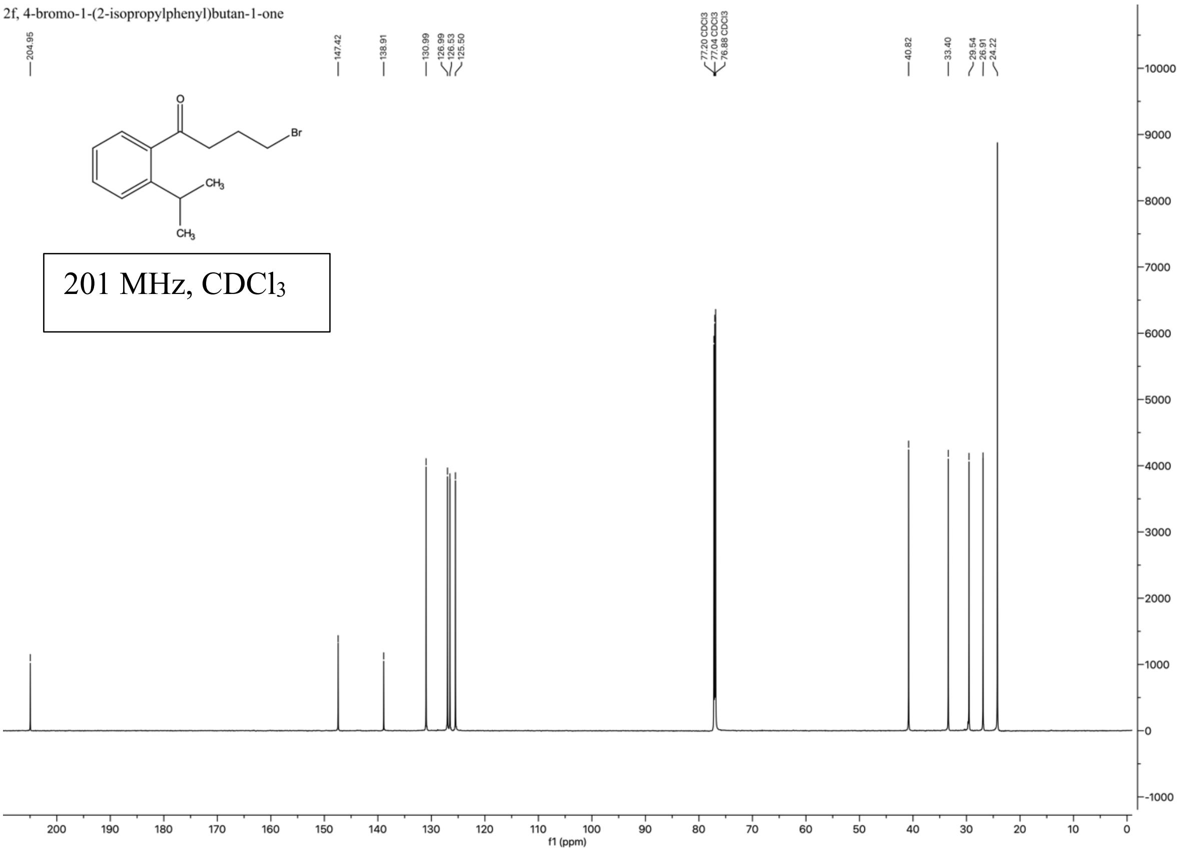
2f, 4-bromo-1-(2-isopropylphenyl)butan-1-one
201 MHz, CDCl3
204.95
147.42
138.91
130.99 126.99 126.53 125.50
77.20 CDCl3 77.04 CDCl3 76.88 CDCl3
40.82
33.40 29.54 26.91 24.22
f1 (ppm)

2g, 4-bromo-1-(3,4,5-trimethoxyphenyl)butan-1-one

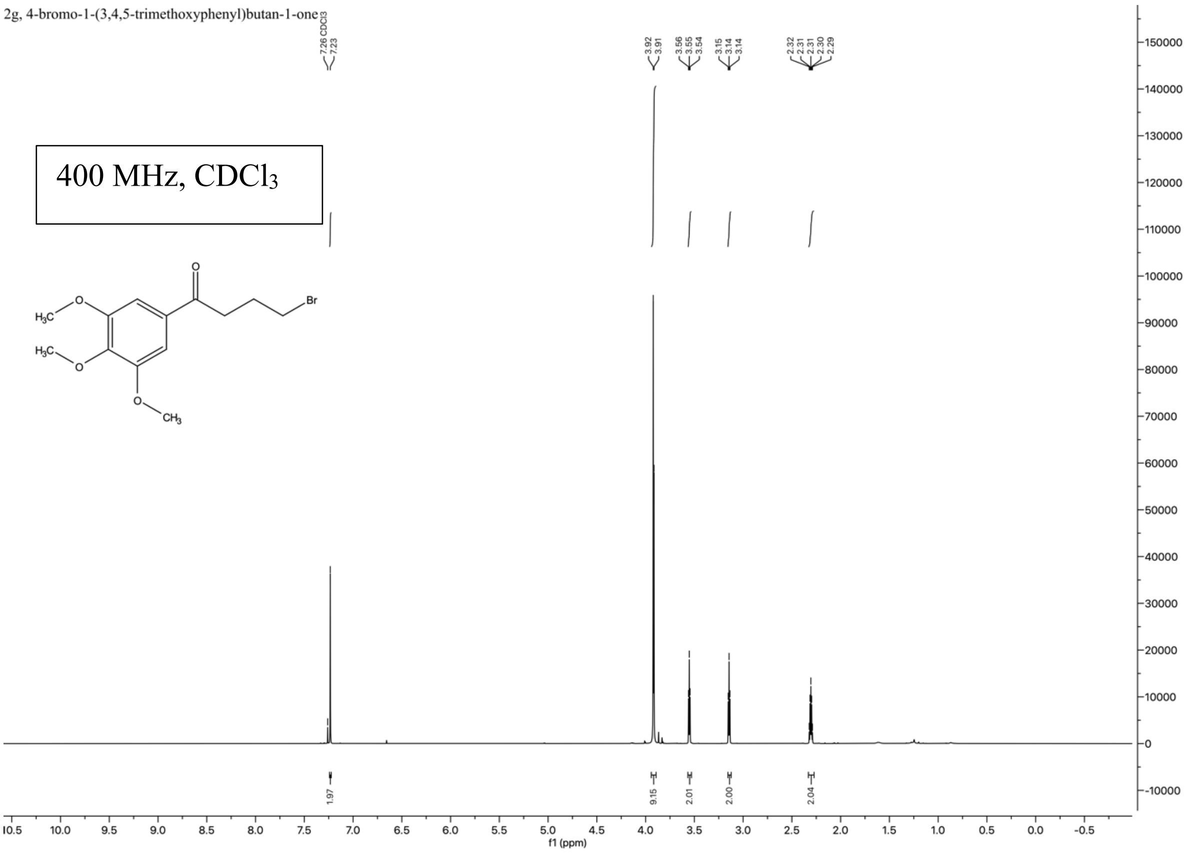

2g, 4-bromo-1-(3,4,5-trimethoxyphenyl)butan-1-one

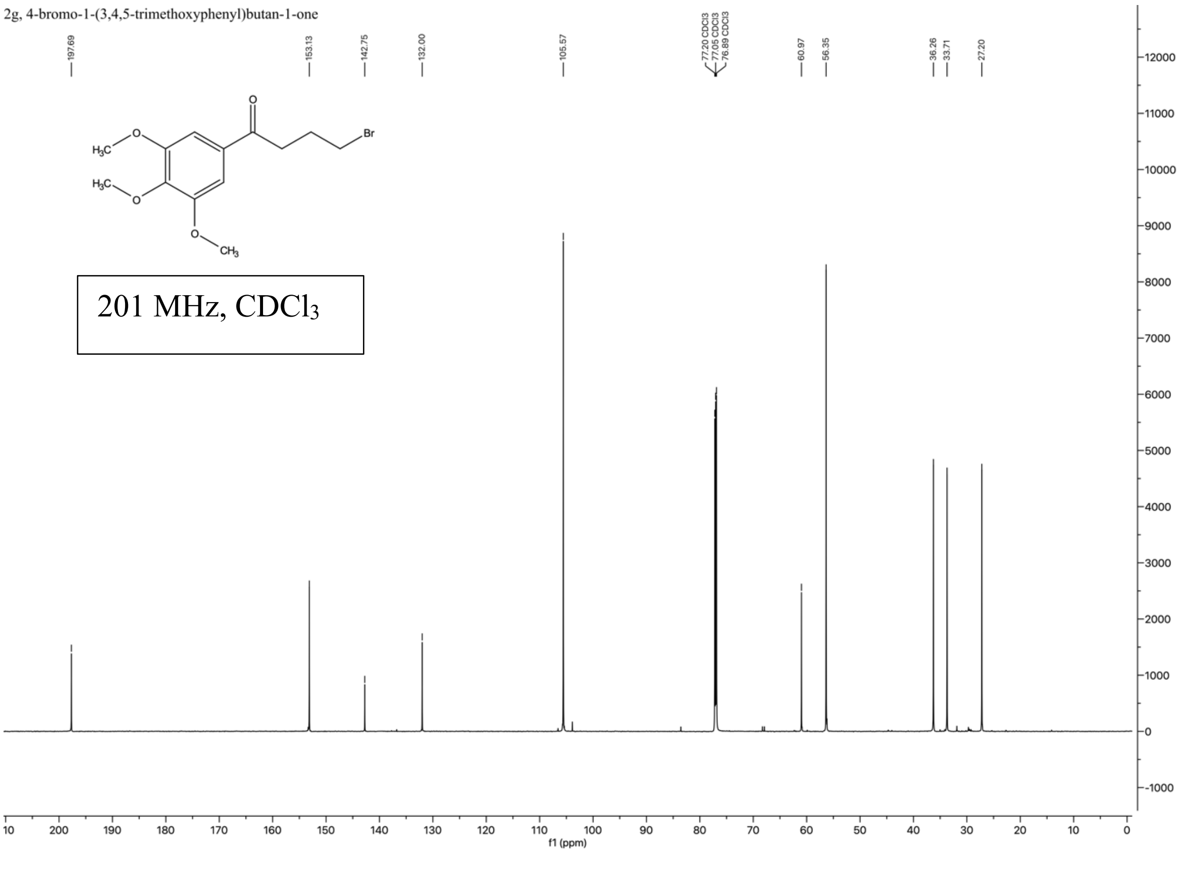

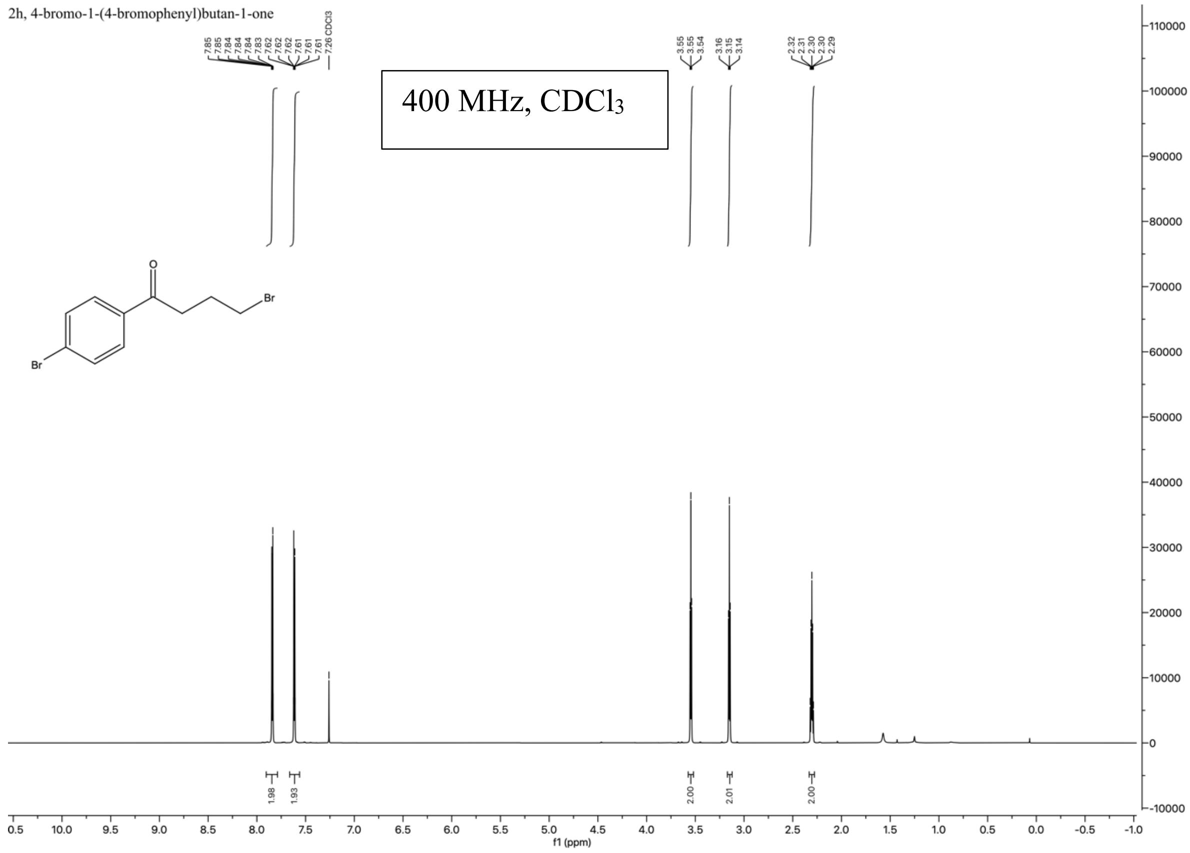
2h, 4-bromo-1-(4-bromophenyl)butan-1-one
400 MHz, CDCl3
7.85
7.85
7.84
7.84
7.84
7.83
7.62
7.62
7.62
7.61
7.61
7.61
7.26 CDCl3
3.55
3.55
3.54
3.16
3.15
3.14
2.32
2.31
2.30
2.30
2.29
1.98
1.93
2.00
2.01
2.00
f1 (ppm)

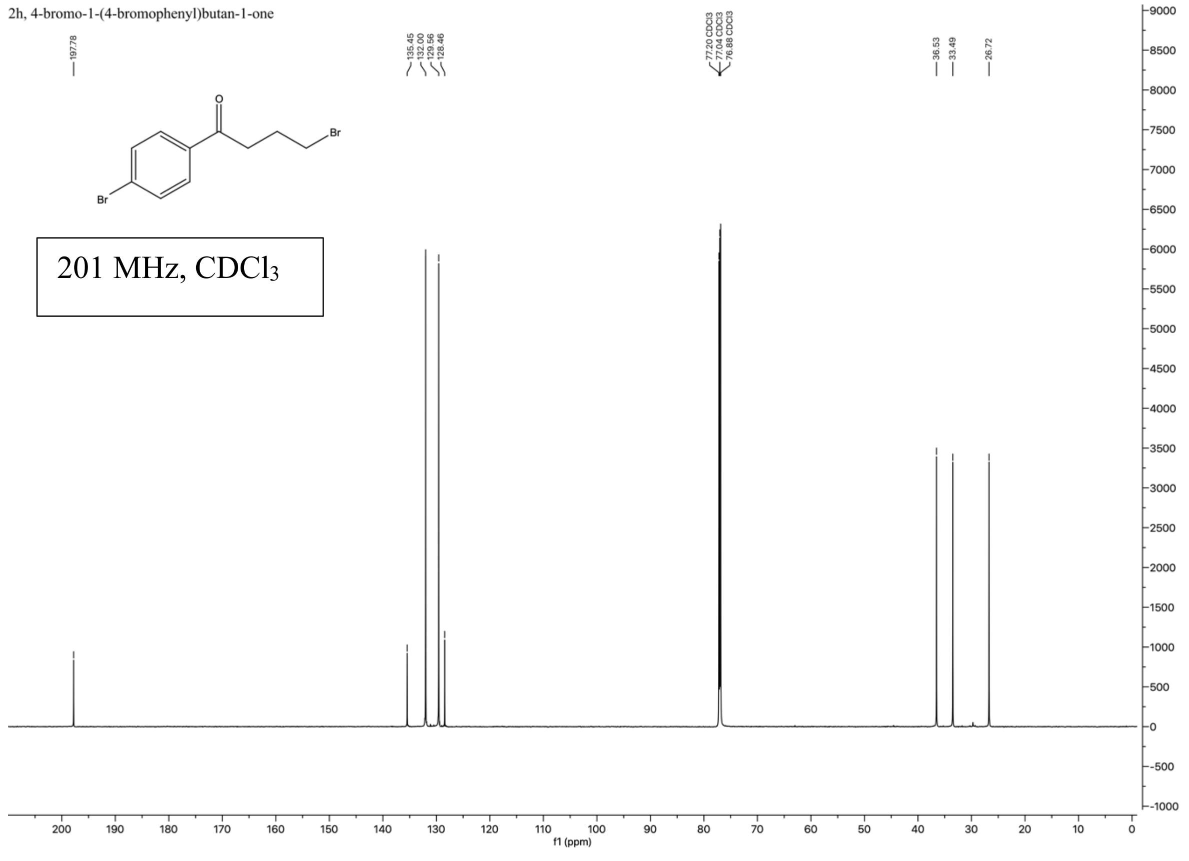
2h, 4-bromo-1-(4-bromophenyl)butan-1-one
201 MHz, CDCl3
197.78
135.45
132.00
129.56
128.46
77.20 CDCl3
77.04 CDCl3
76.88 CDCl3
36.53
33.49
26.72
f1 (ppm)

2i, 4-bromo-1-(p-tolyl)butan-1-one

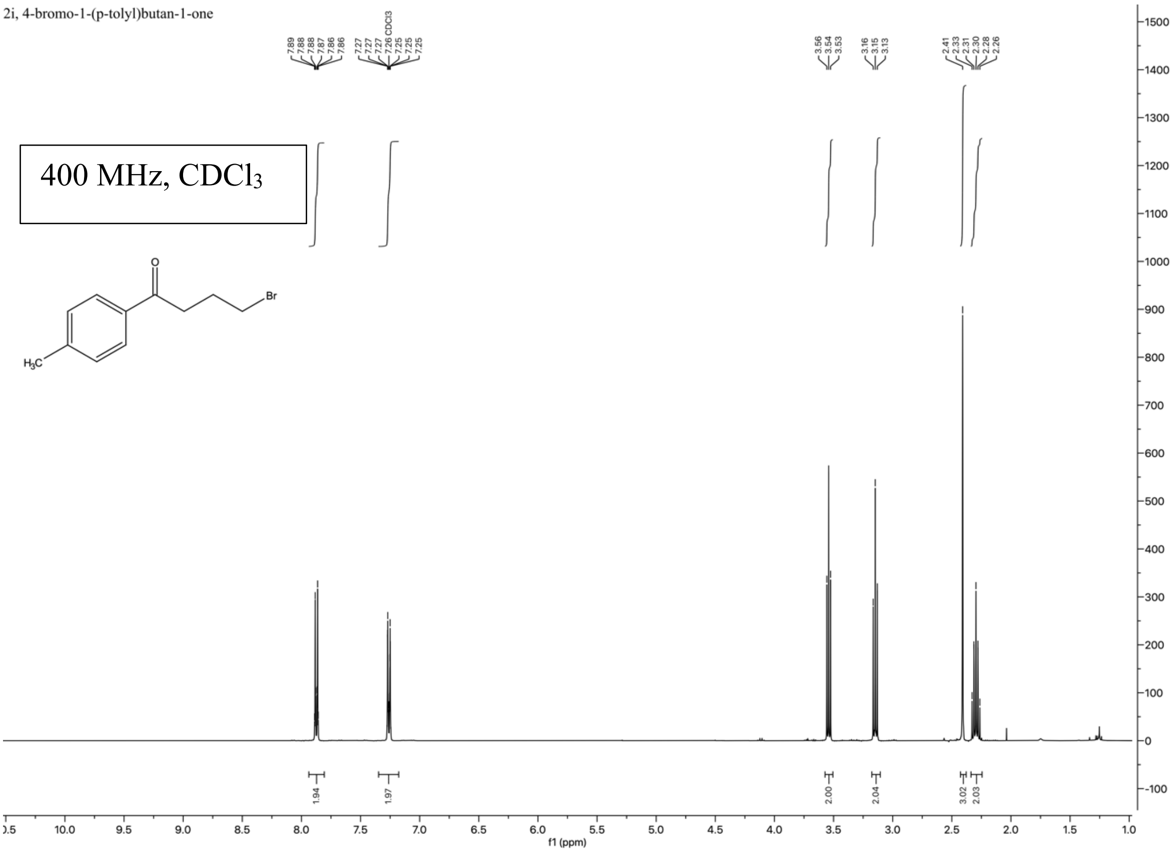

2i, 4-bromo-1-(p-tolyl)butan-1-one

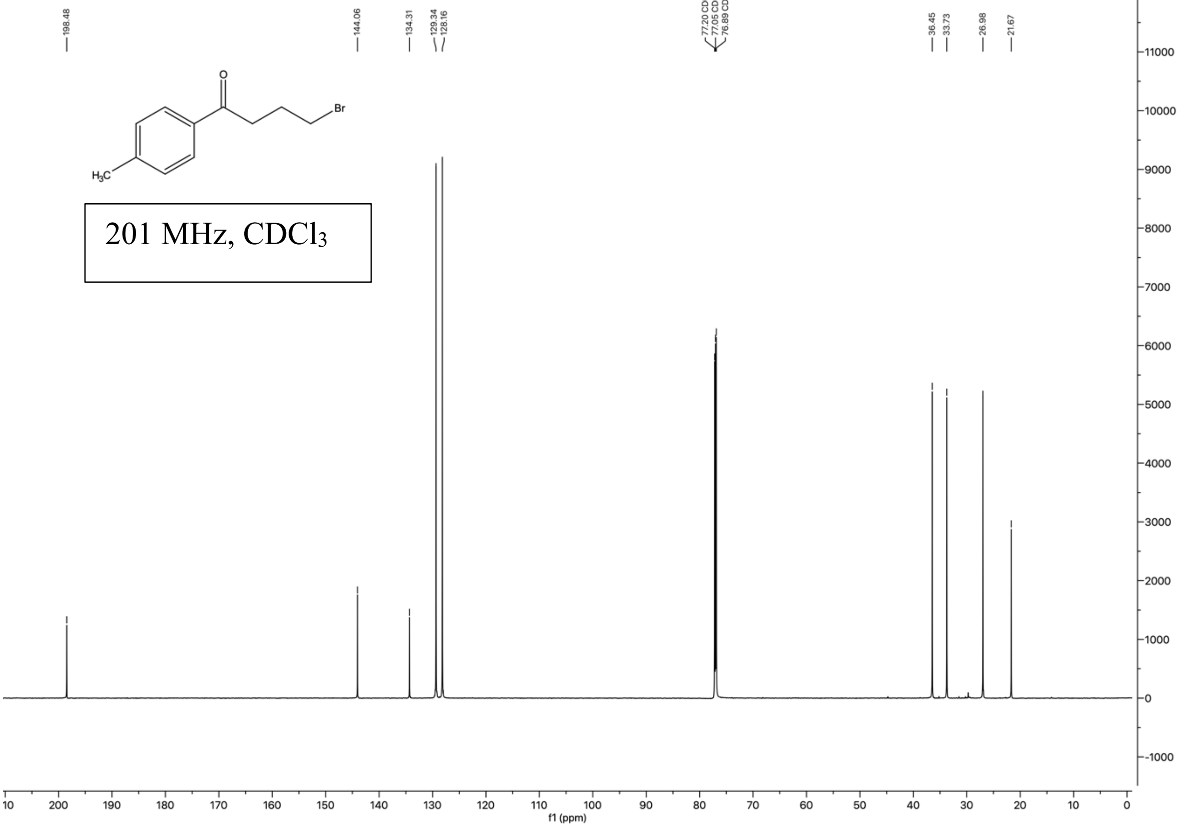

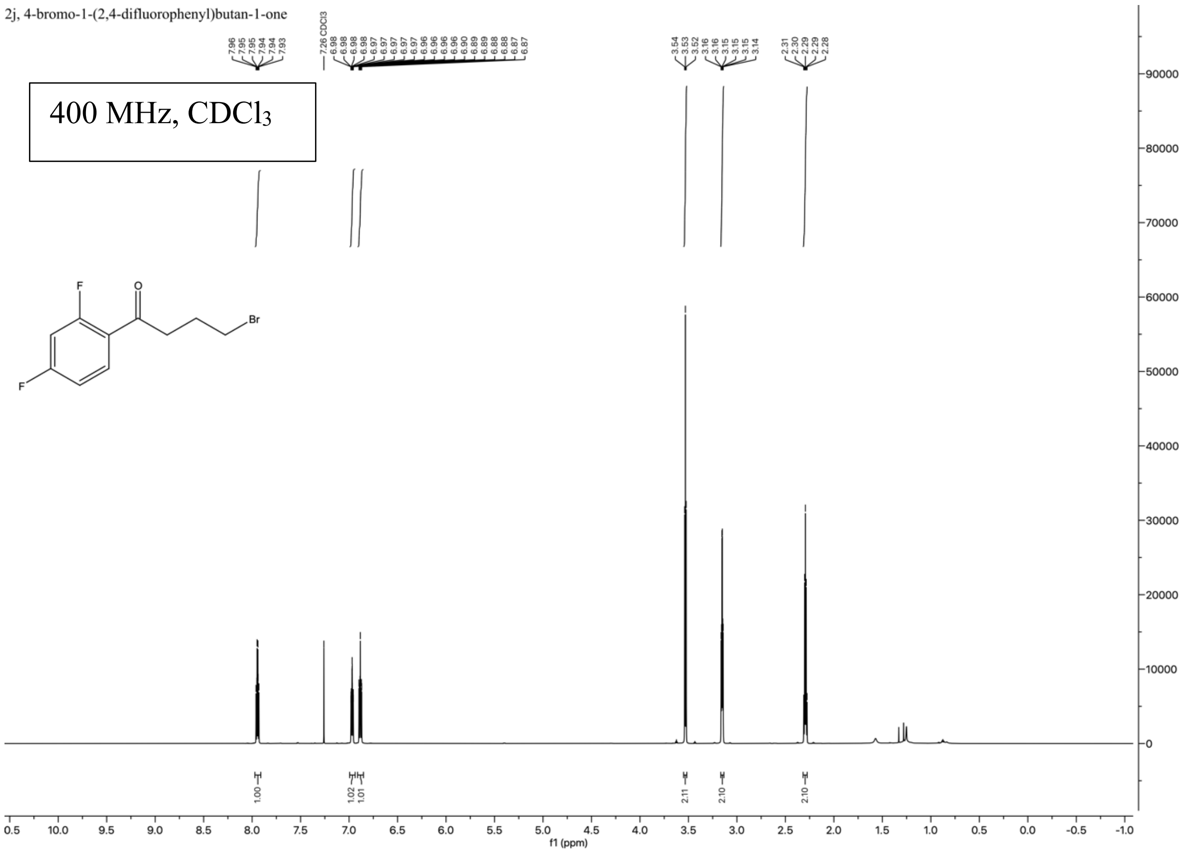
2j, 4-bromo-1-(2,4-difluorophenyl)butan-1-one
400 MHz, CDCl3
7.96 7.95 7.95 7.94 7.94 7.93
7.26 CDCl3
3.54 3.53 3.52 3.16 3.16 3.15 3.15 3.15 3.14
2.31 2.30 2.29 2.29 2.28
f1 (ppm)

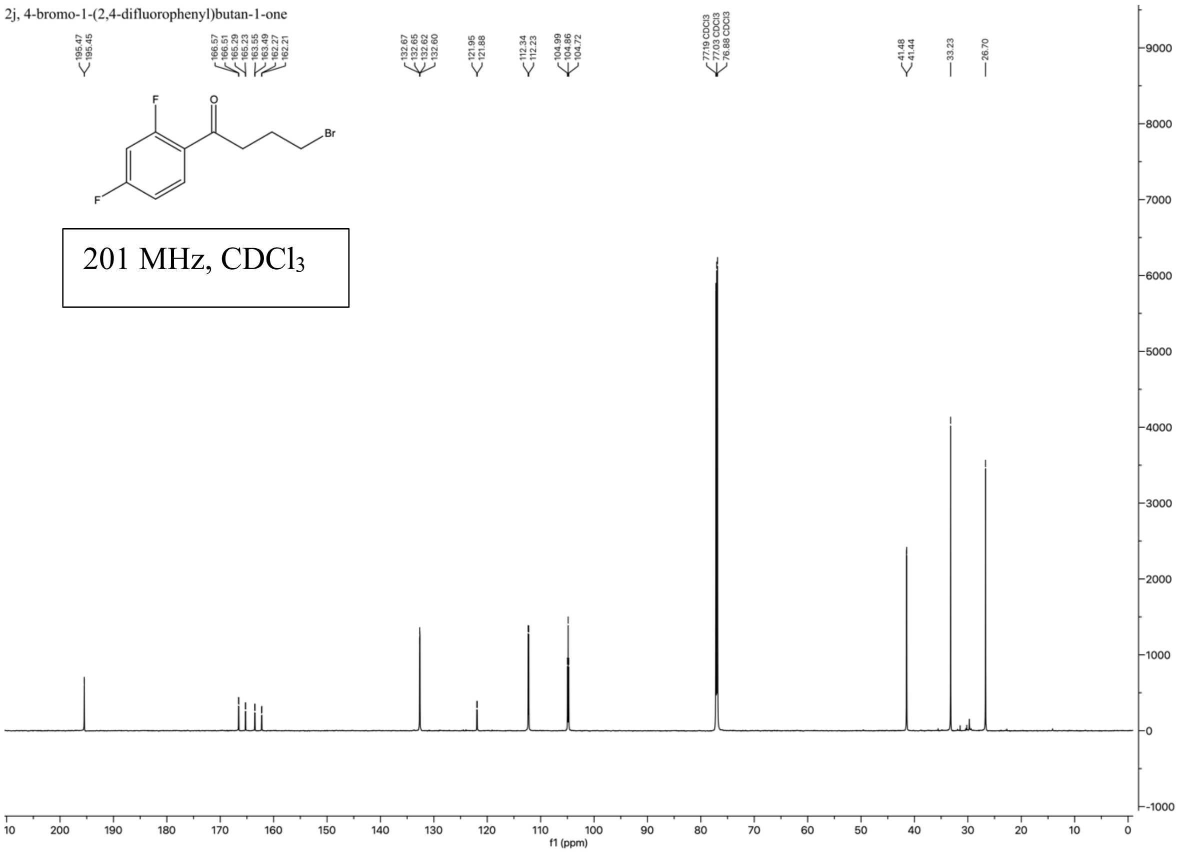
2j, 4-bromo-1-(2,4-difluorophenyl)butan-1-one
201 MHz, CDCl3
195.47 195.45
166.57 166.51 165.29 165.23 163.55 163.49 162.27 162.21
132.67 132.65 132.62 132.60
121.95 121.88
112.34 112.23
104.99 104.86 104.72
77.19 CDCl3 77.03 CDCl3 76.88 CDCl3
41.48 41.44
33.23
26.70
f1 (ppm)

2j, 4-bromo-1-(2,4-difluorophenyl)butan-1-one

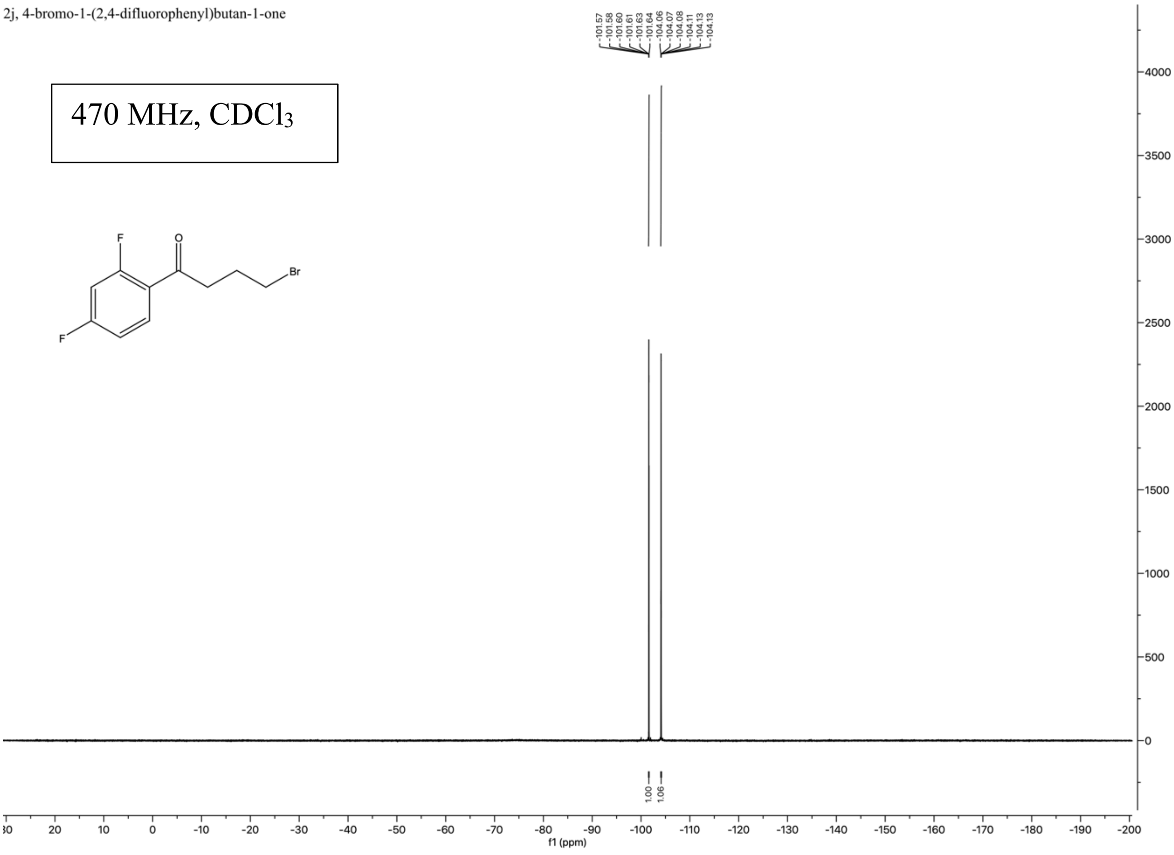

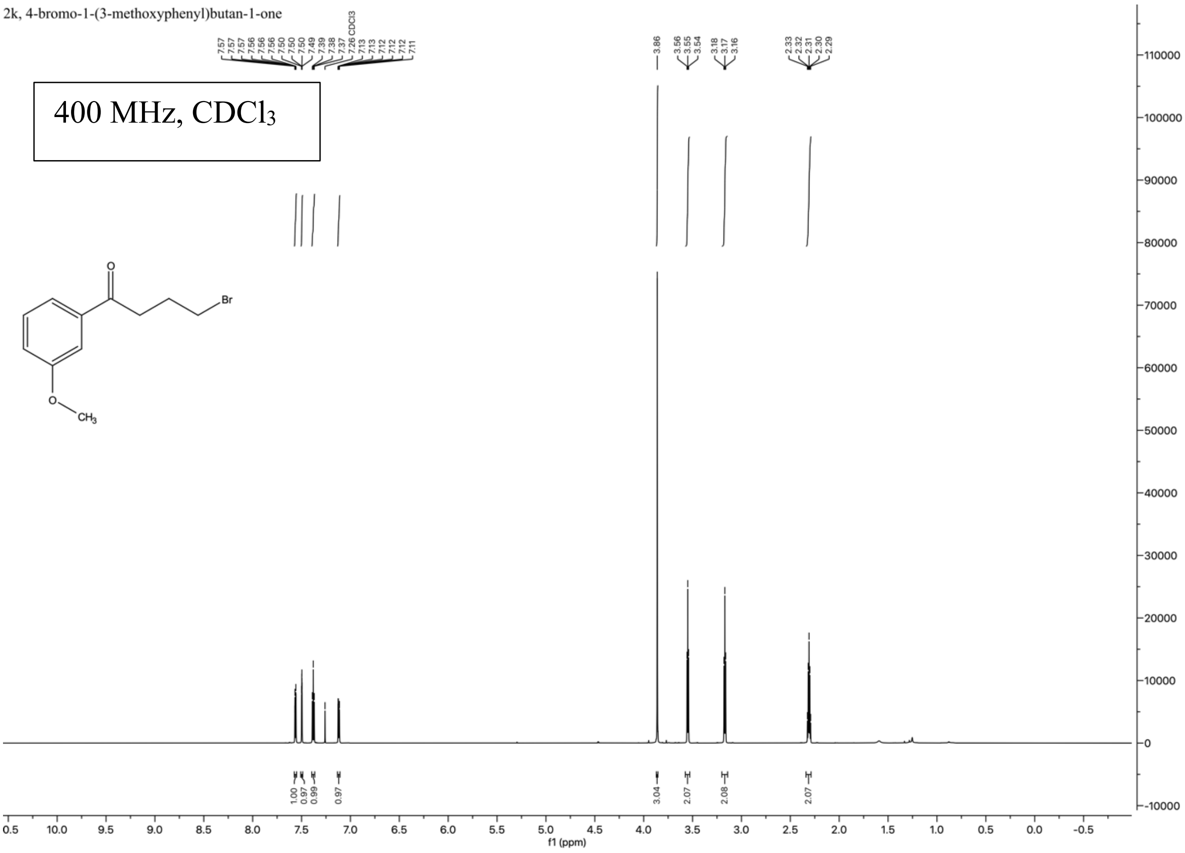
2k, 4-bromo-1-(3-methoxyphenyl)butan-1-one
400 MHz, CDCl3
f1 (ppm)

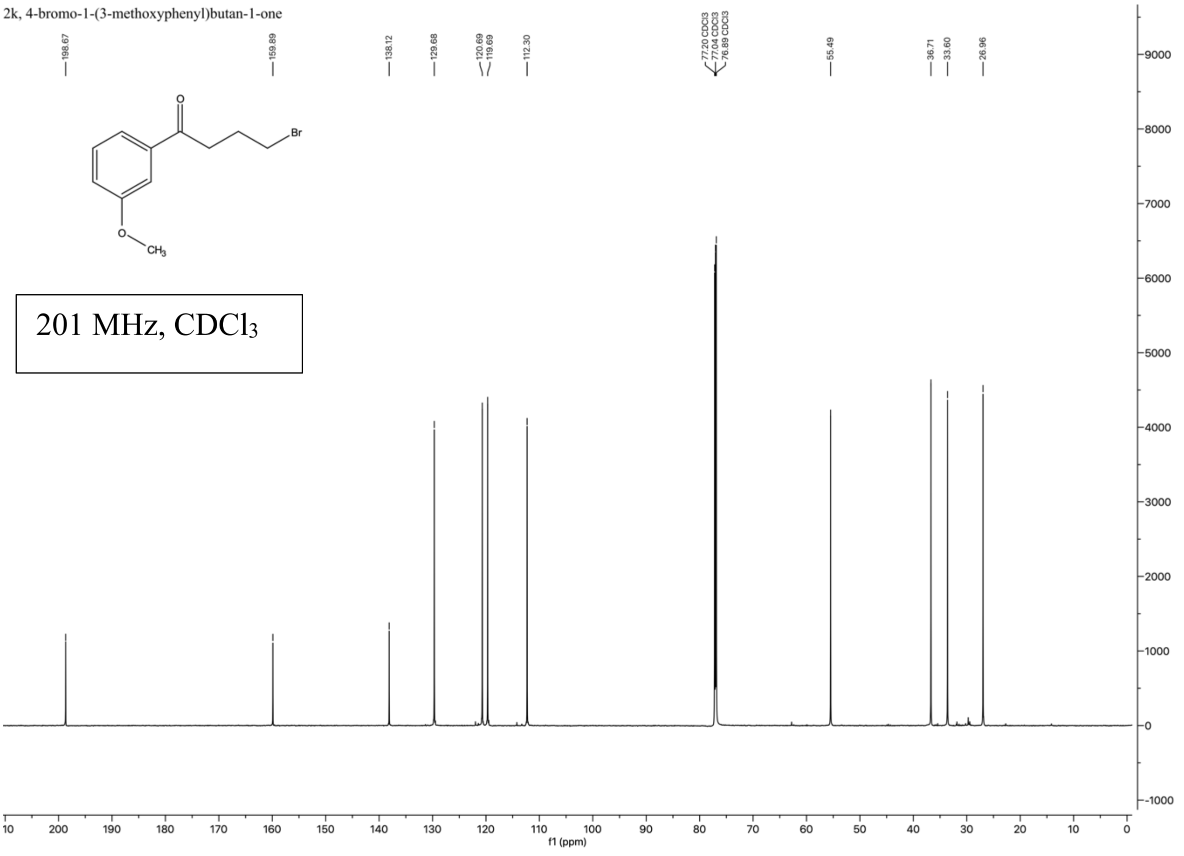
2k, 4-bromo-1-(3-methoxyphenyl)butan-1-one
201 MHz, CDCl3
f1 (ppm)

2l, 2-(3-(trichloromethyl)tetrahydrofuran-2-yl)benzo[d]thiazole

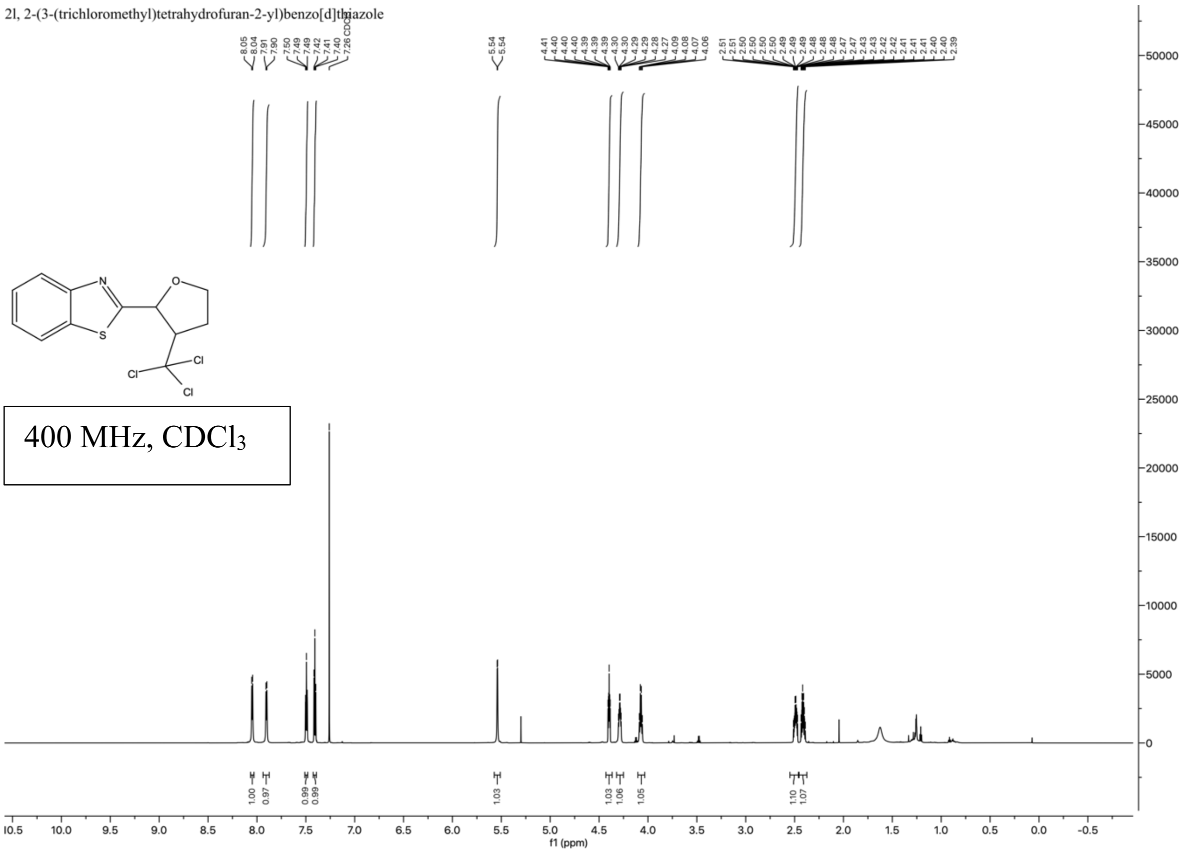

2l, 2-(3-(trichloromethyl)tetrahydrofuran-2-yl)benzo[d]thiazole

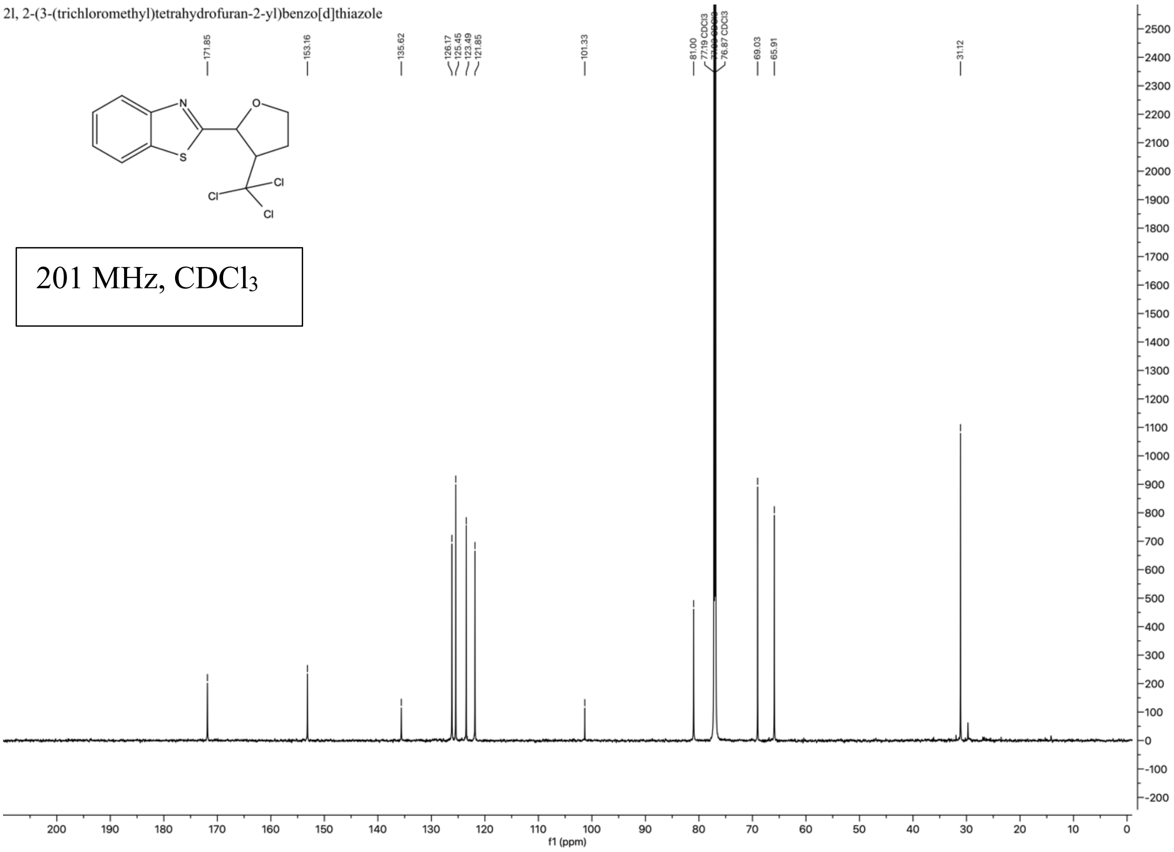

3a, 4-bromo-2-methyl-1-phenylbutan-1-one

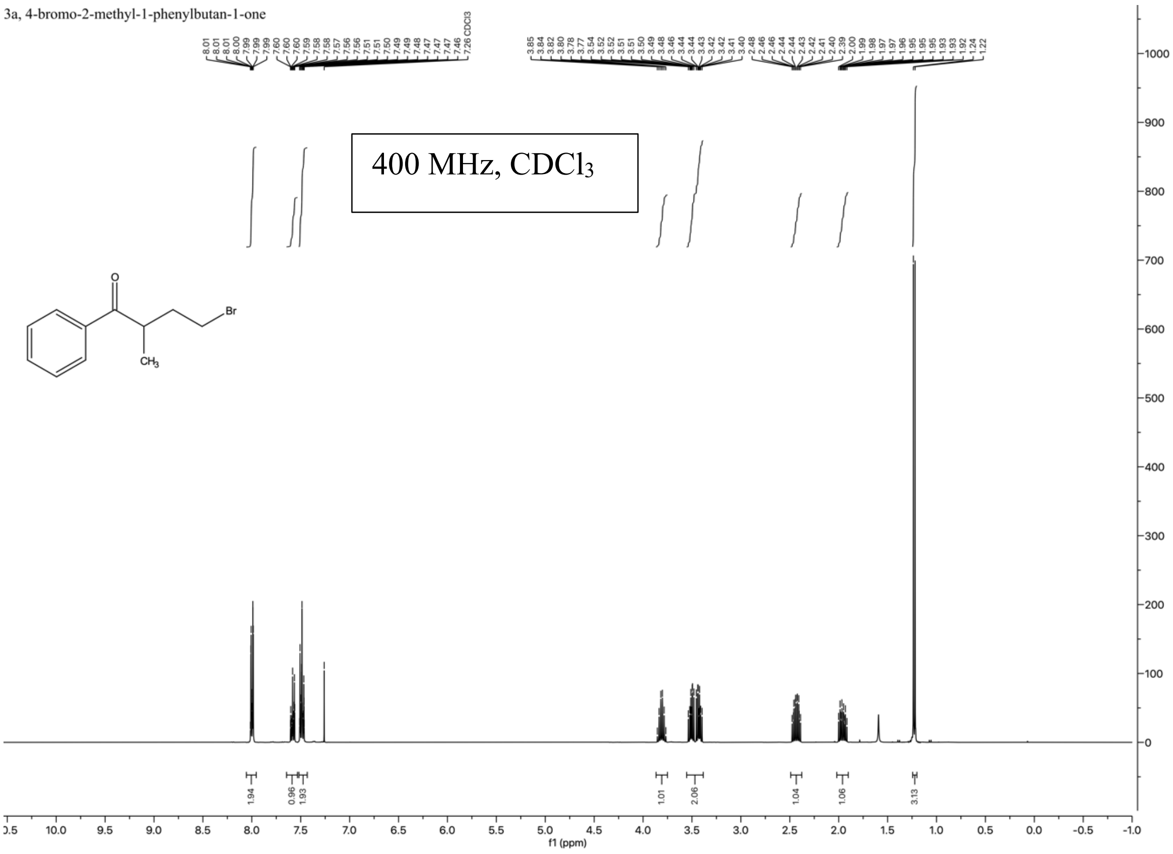

3a, 4-bromo-2-methyl-1-phenylbutan-1-one

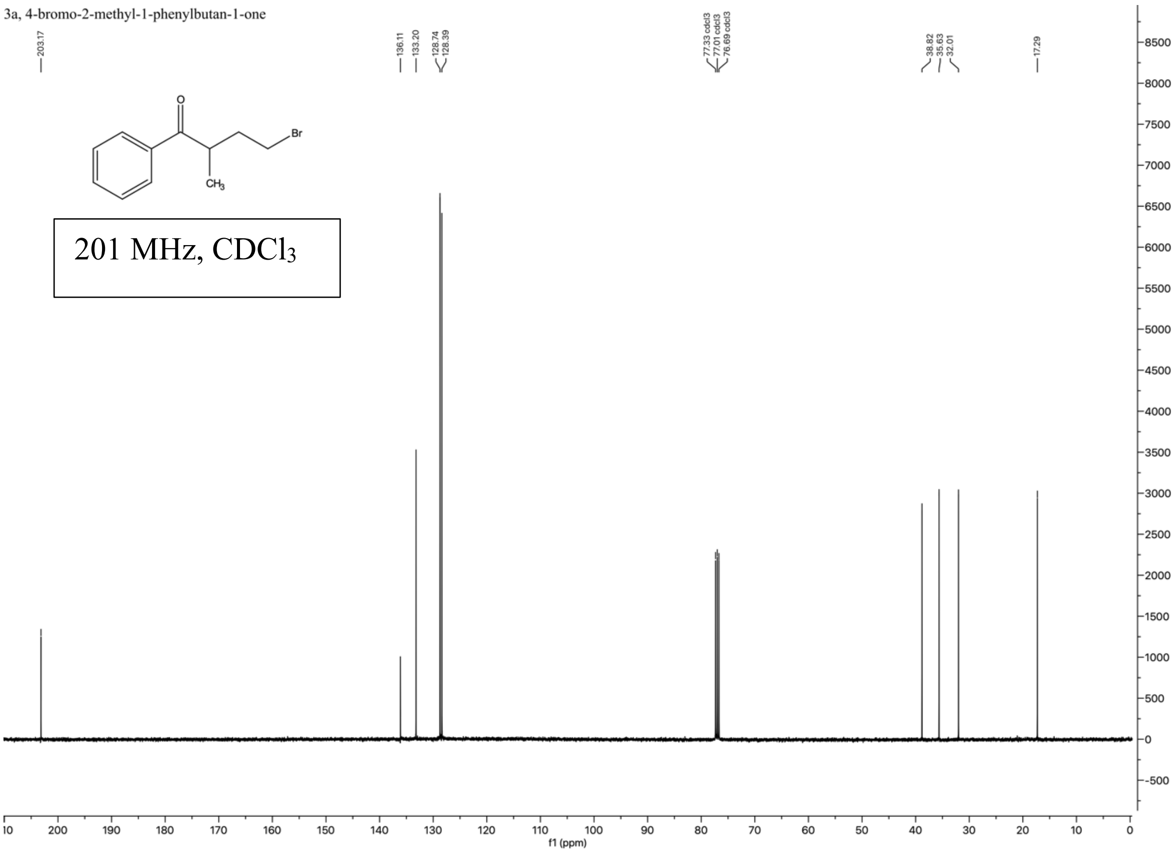

3b, 5-bromo-1-phenylpentan-1-one

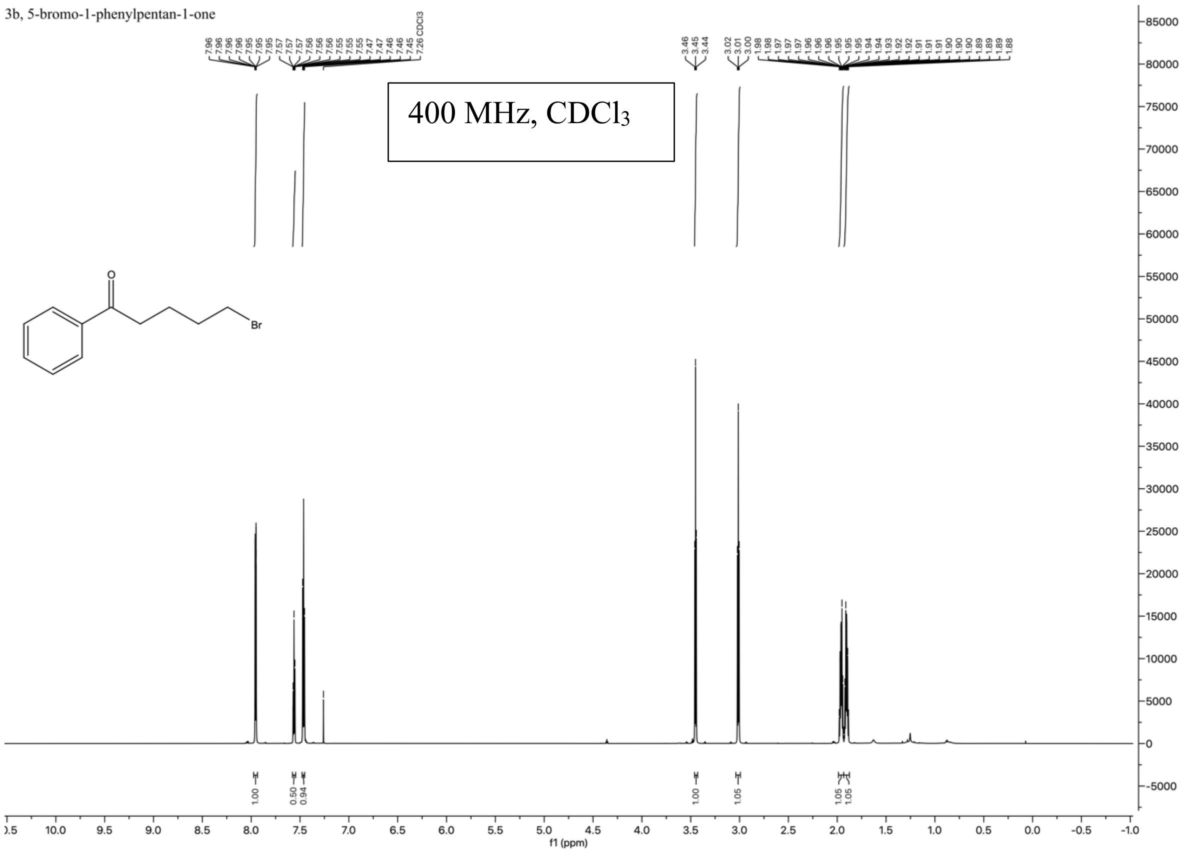

3b, 5-bromo-1-phenylpentan-1-one

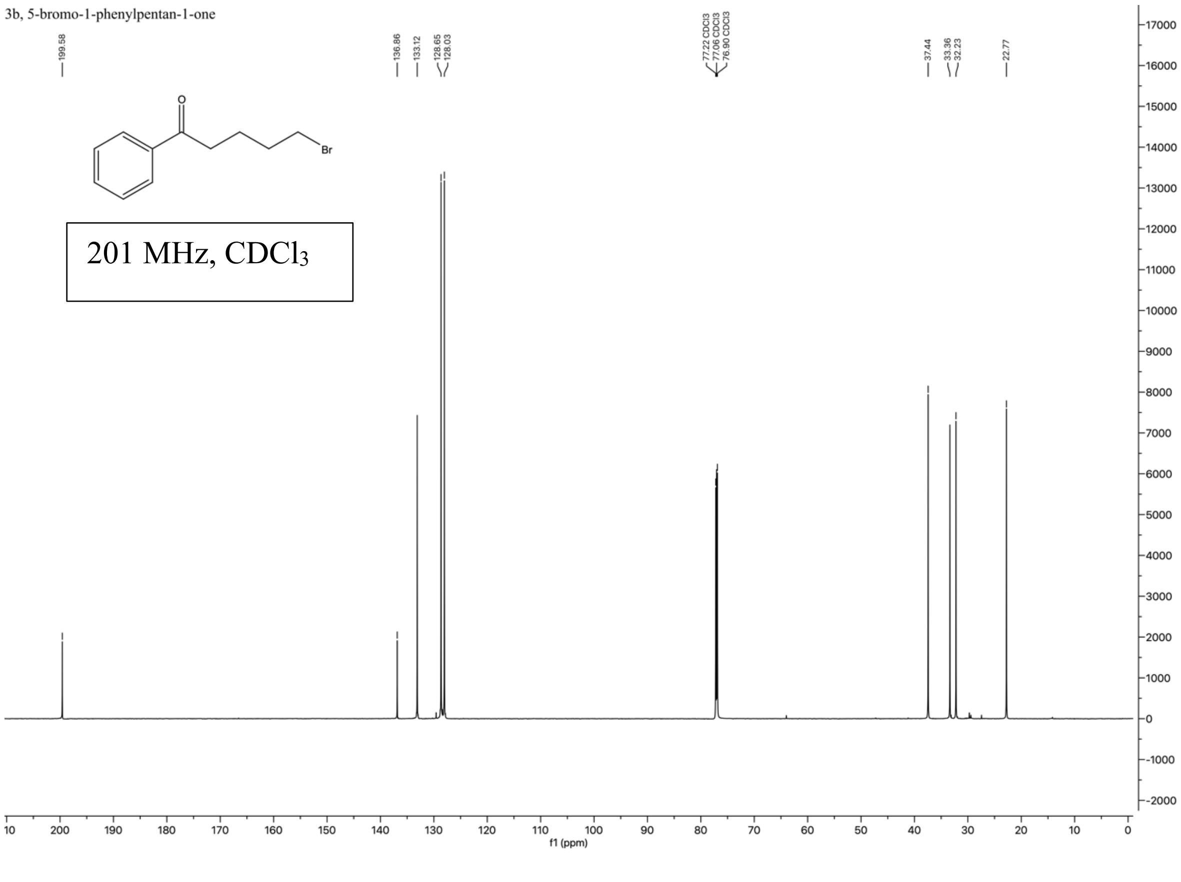

3d, methyl benzoate

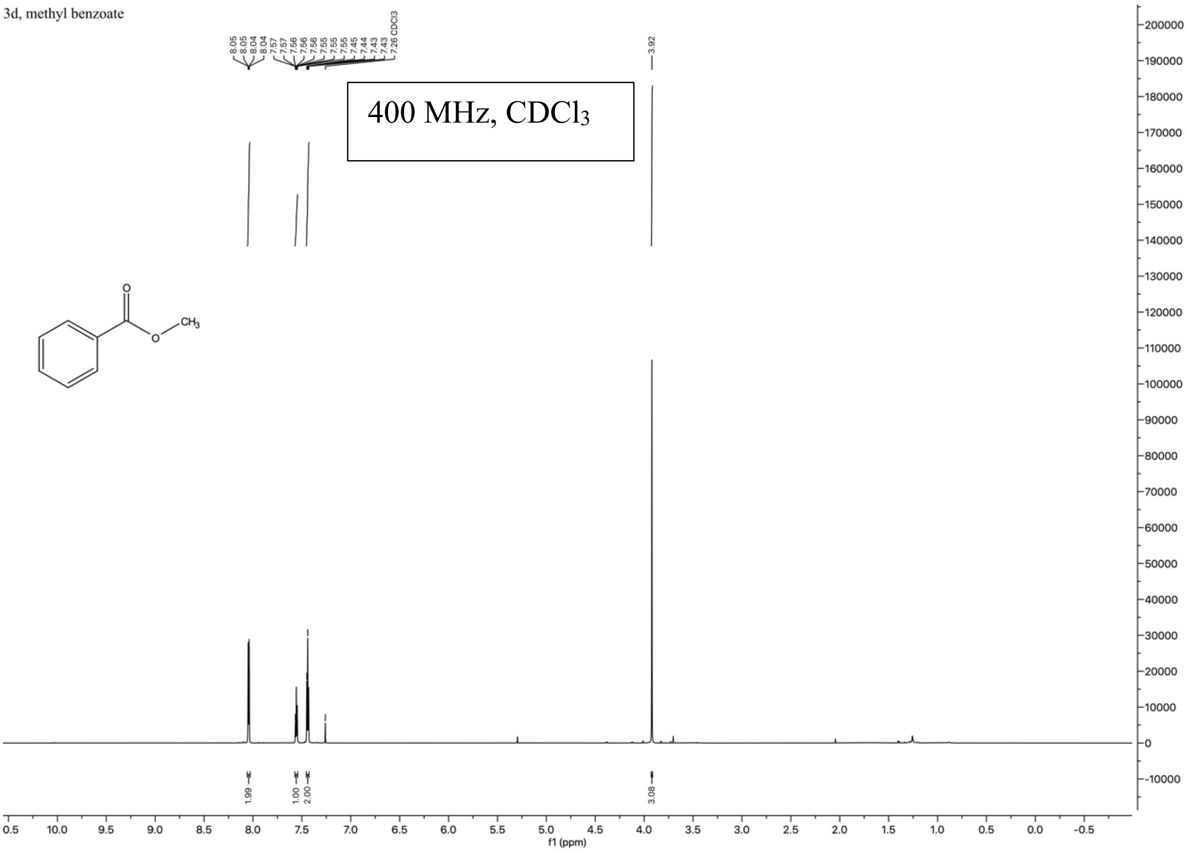

3d, methyl benzoate

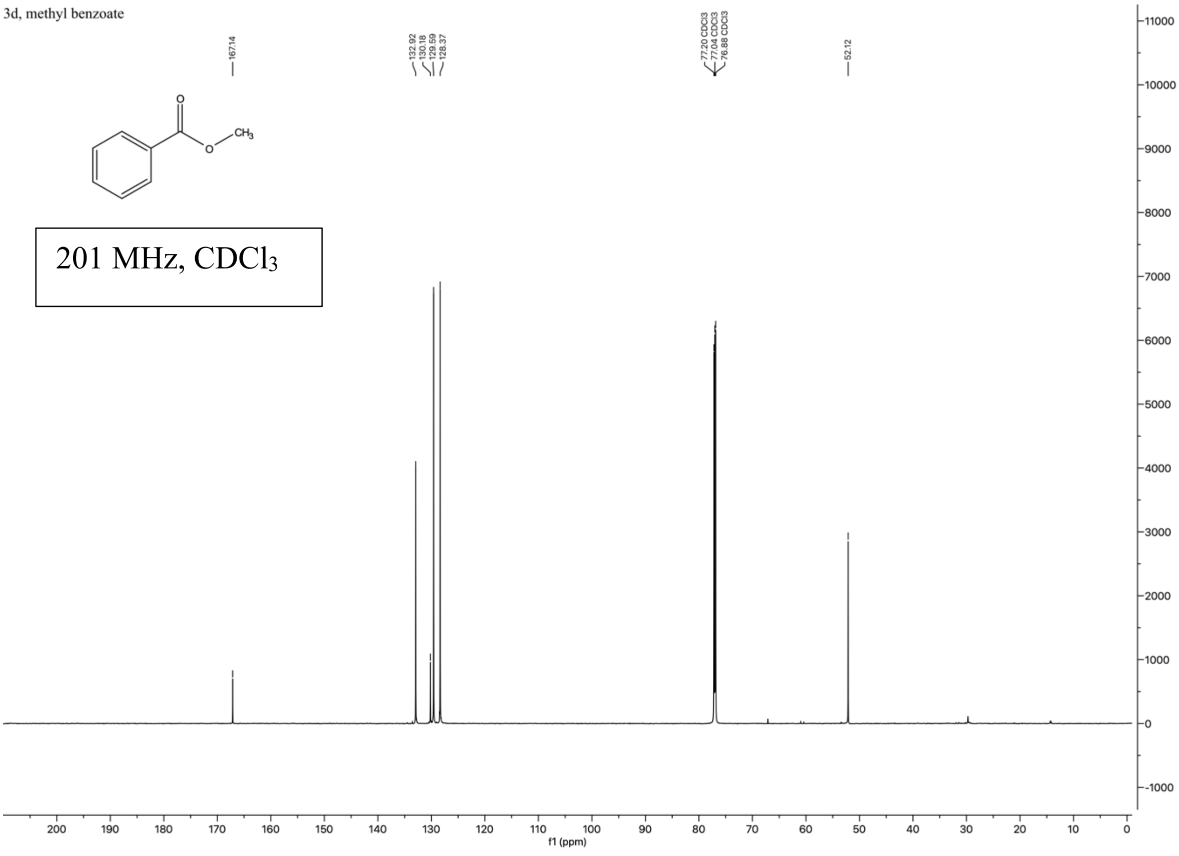

3e, 2-bromoethyl 3-bromobenzoate

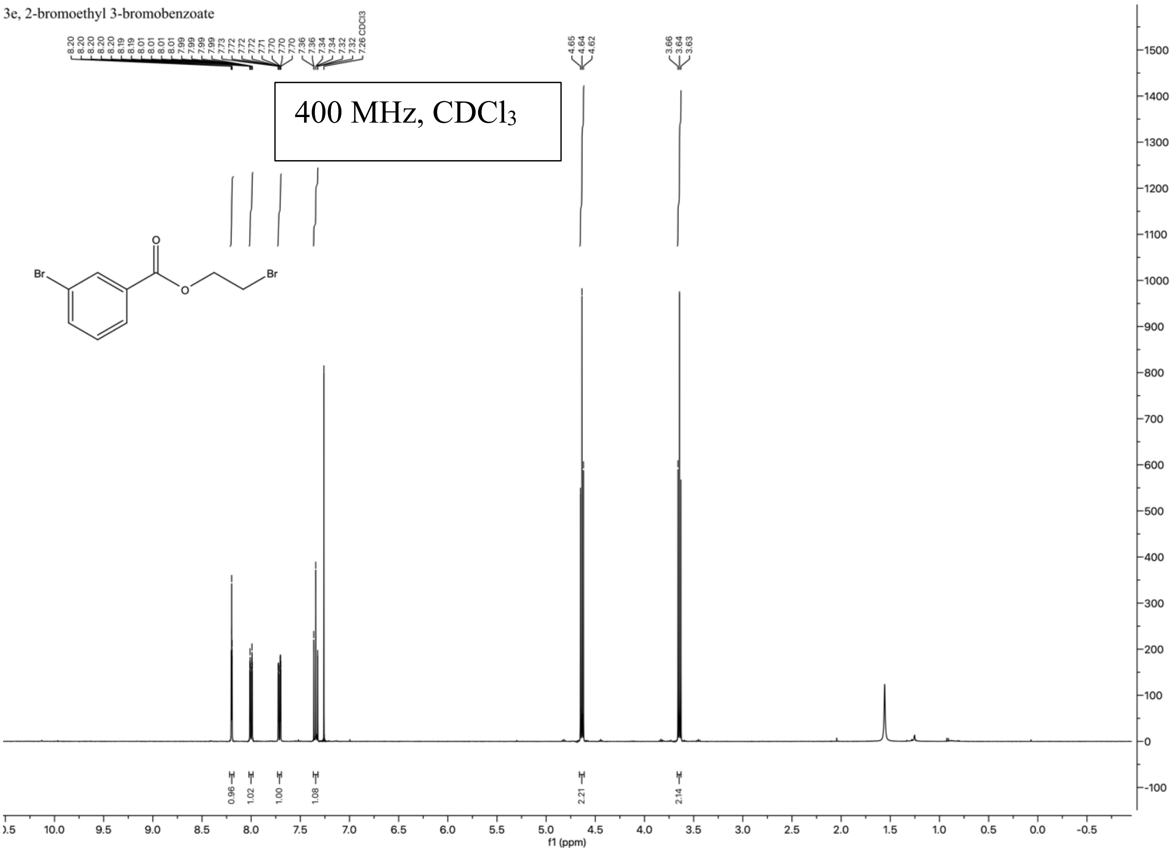

3e, 2-bromoethyl 3-bromobenzoate

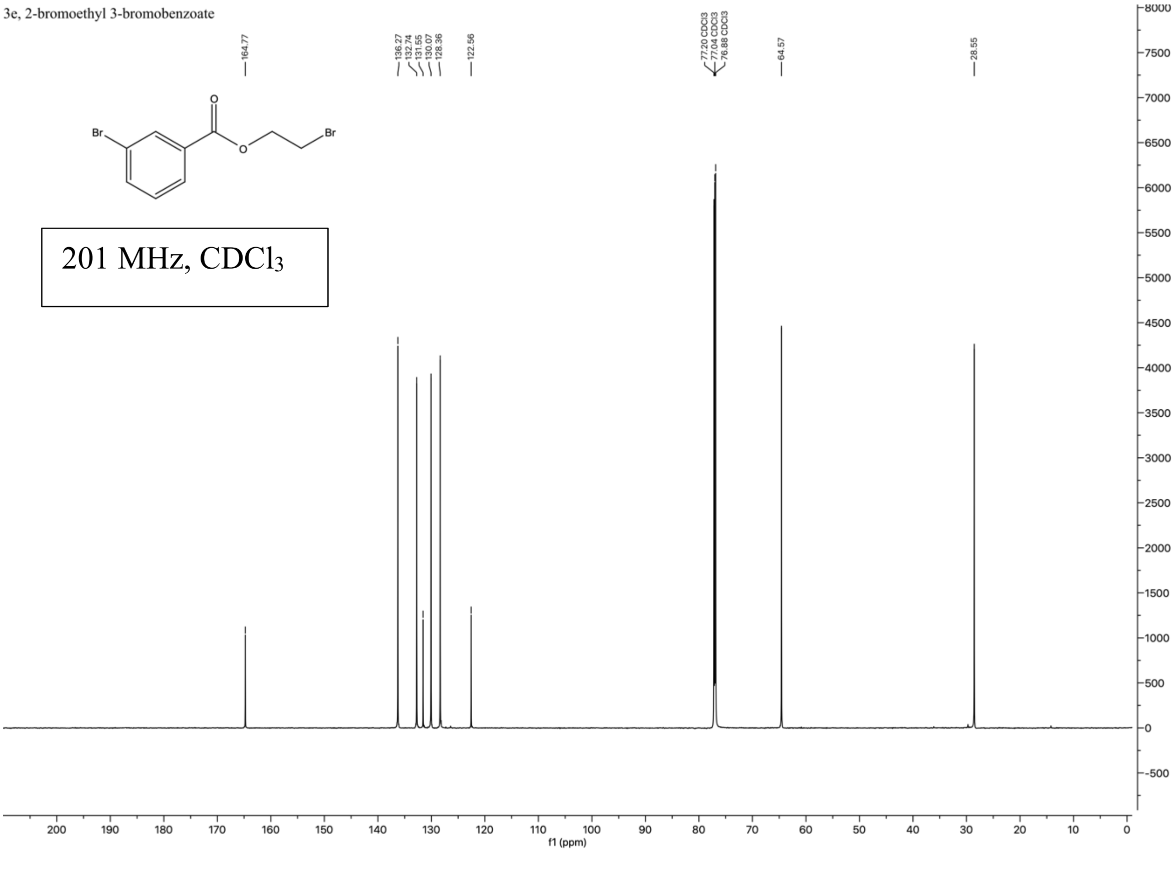

3f, 1-bromopropan-2-yl benzoate

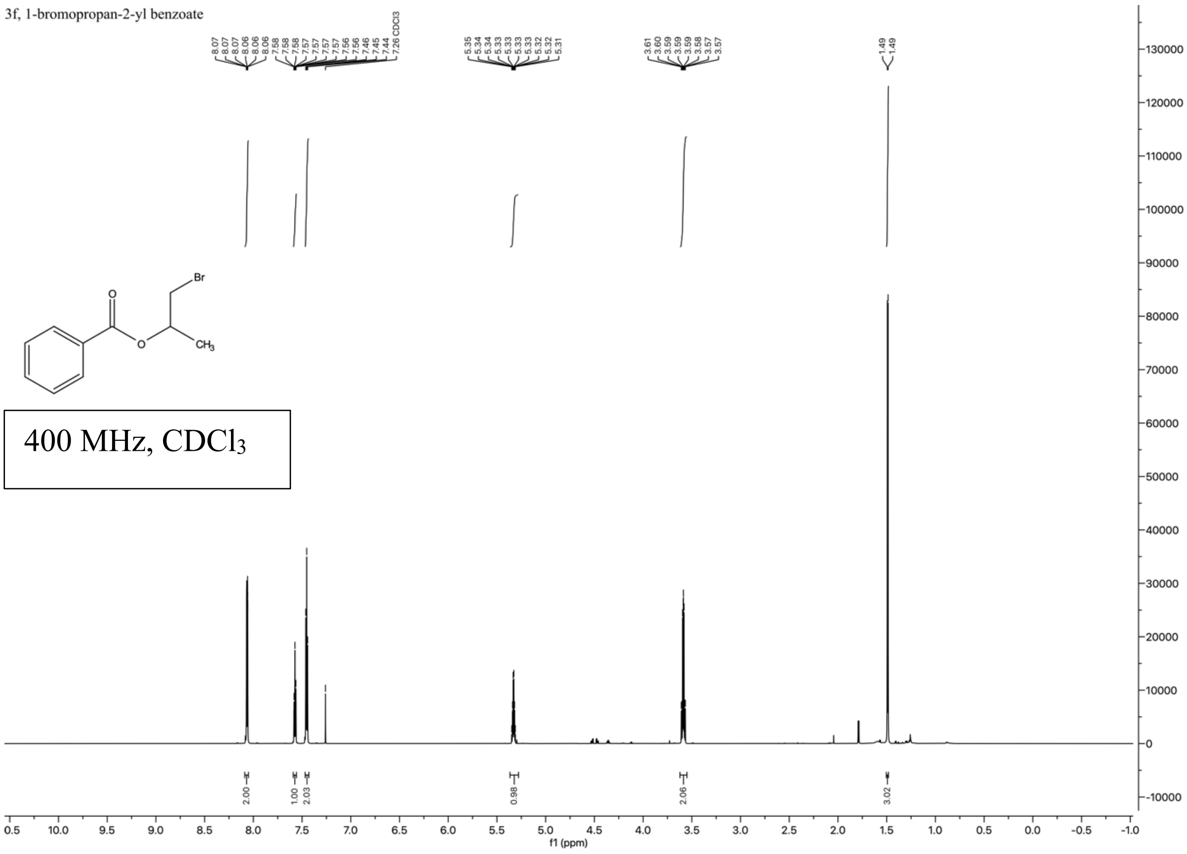

3f, 1-bromopropan-2-yl benzoate

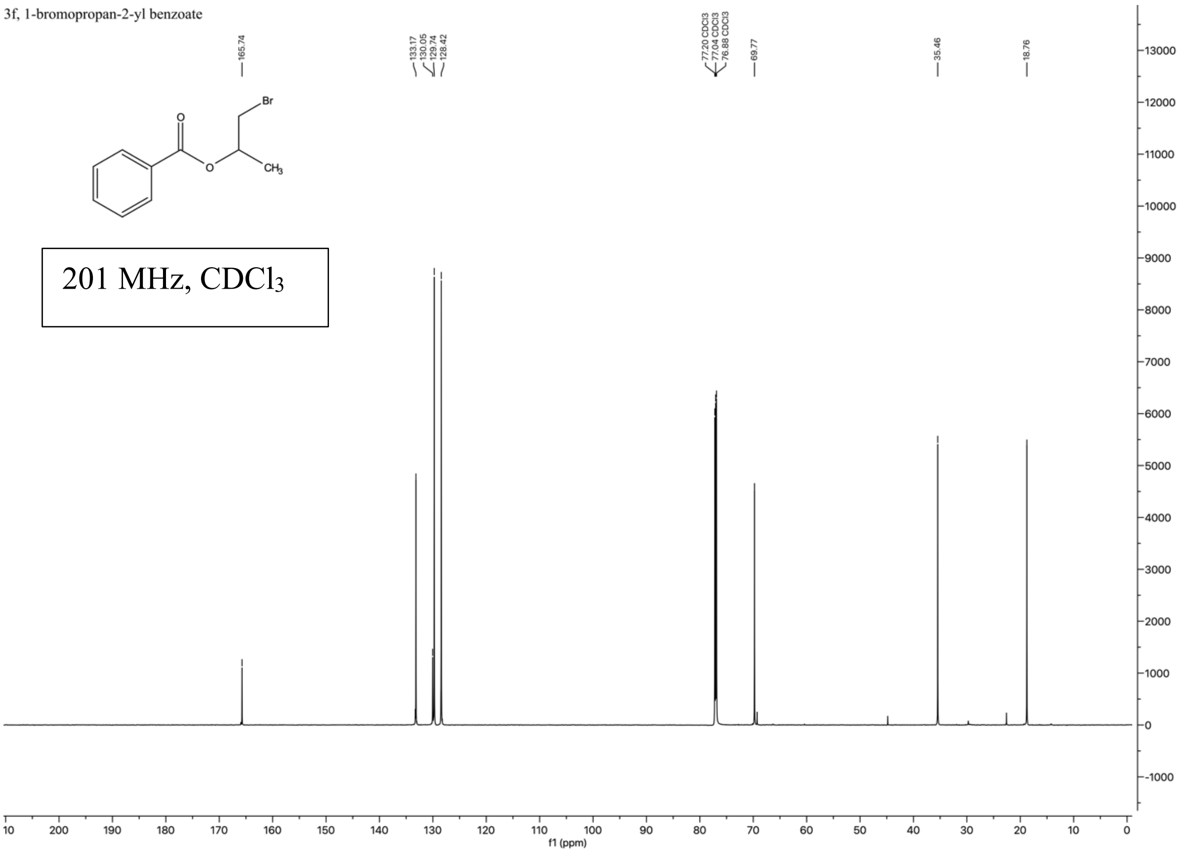

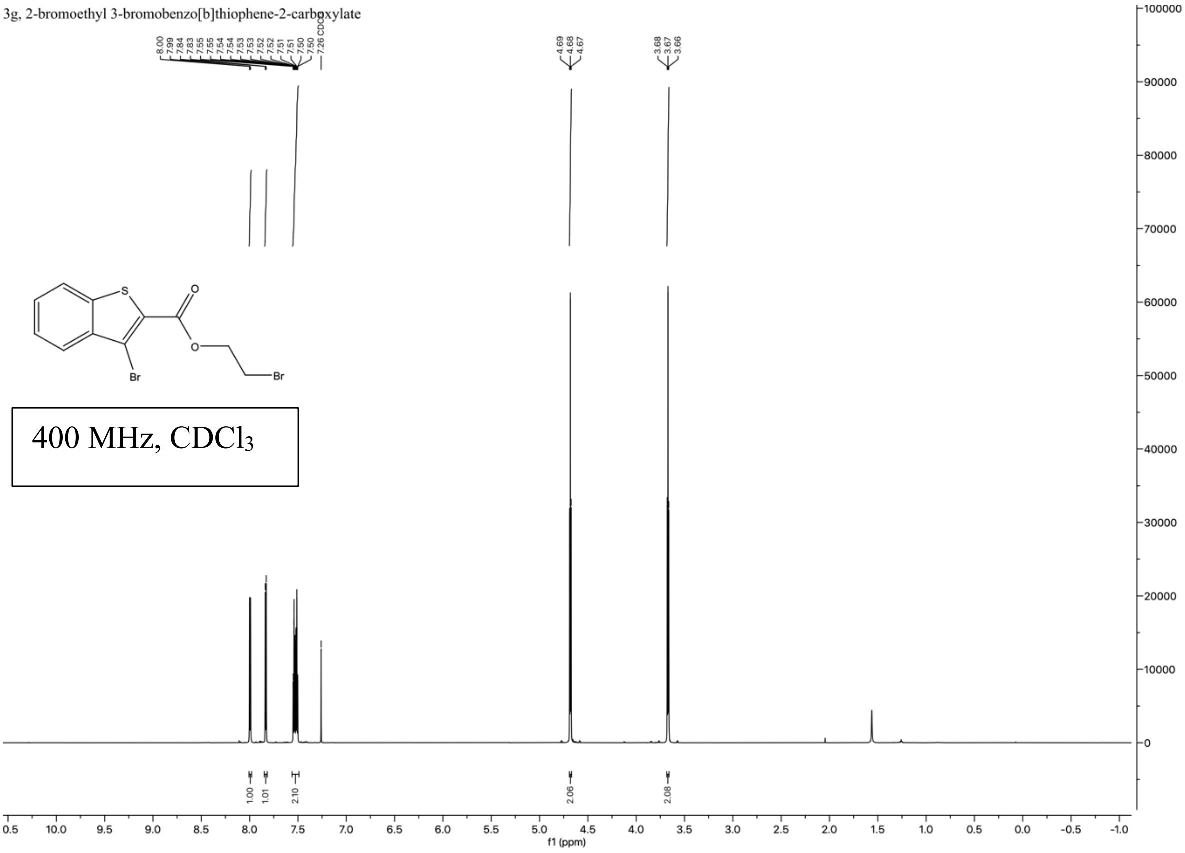
3g, 2-bromoethyl 3-bromobenzo[b]thiophene-2-carboxylate
8.00 7.99 7.84 7.83 7.55 7.55 7.54 7.54 7.53 7.53 7.52 7.52 7.51 7.51 7.50 7.50 7.26 CDCl3
4.69 4.68 4.67
3.68 3.67 3.66
400 MHz, CDCl3
1.00 1.01 2.10 2.06 2.08
f1 (ppm)

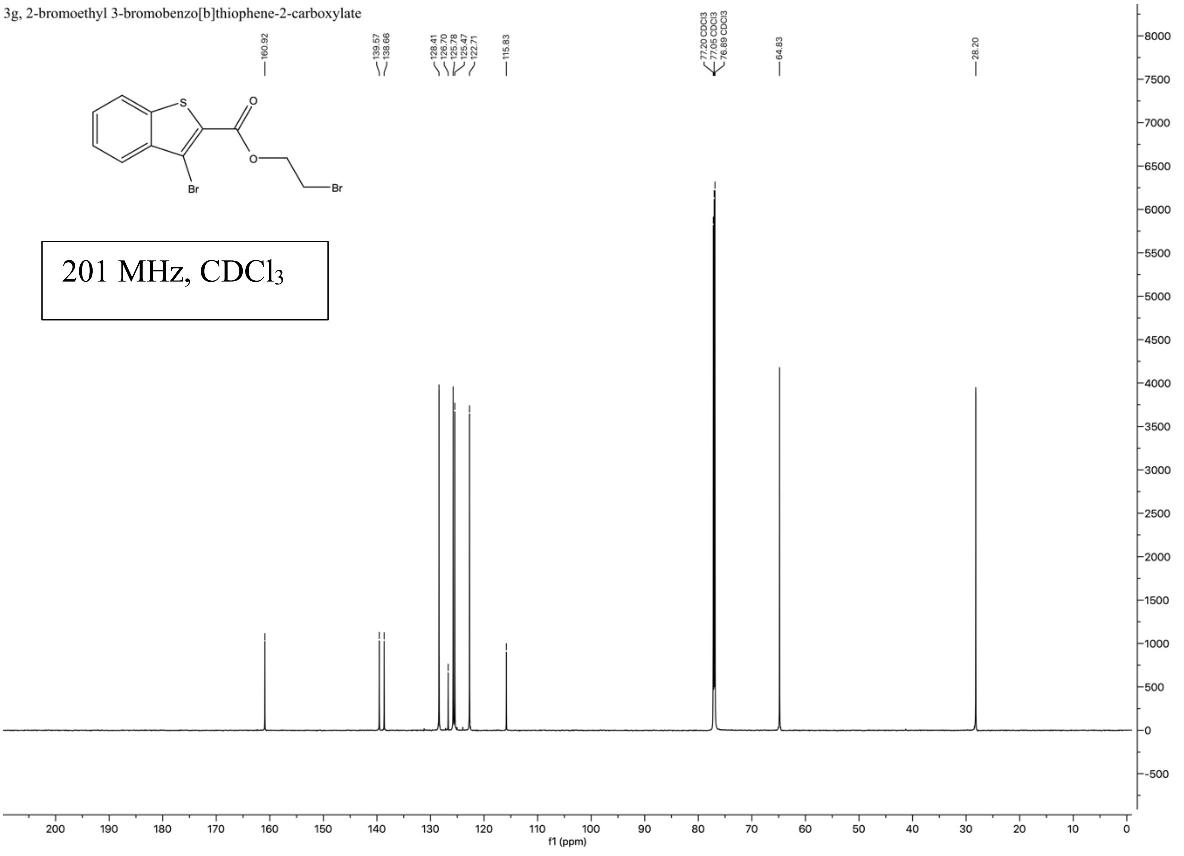
3g, 2-bromoethyl 3-bromobenzo[b]thiophene-2-carboxylate
160.92
139.57 138.66
128.41 126.70 125.78 125.47 122.71
115.83
77.20 CDCl3 77.05 CDCl3 76.89 CDCl3
64.83
28.20
201 MHz, CDCl3
f1 (ppm)

3h, 2-bromoethyl benzofuran-2-carboxylate

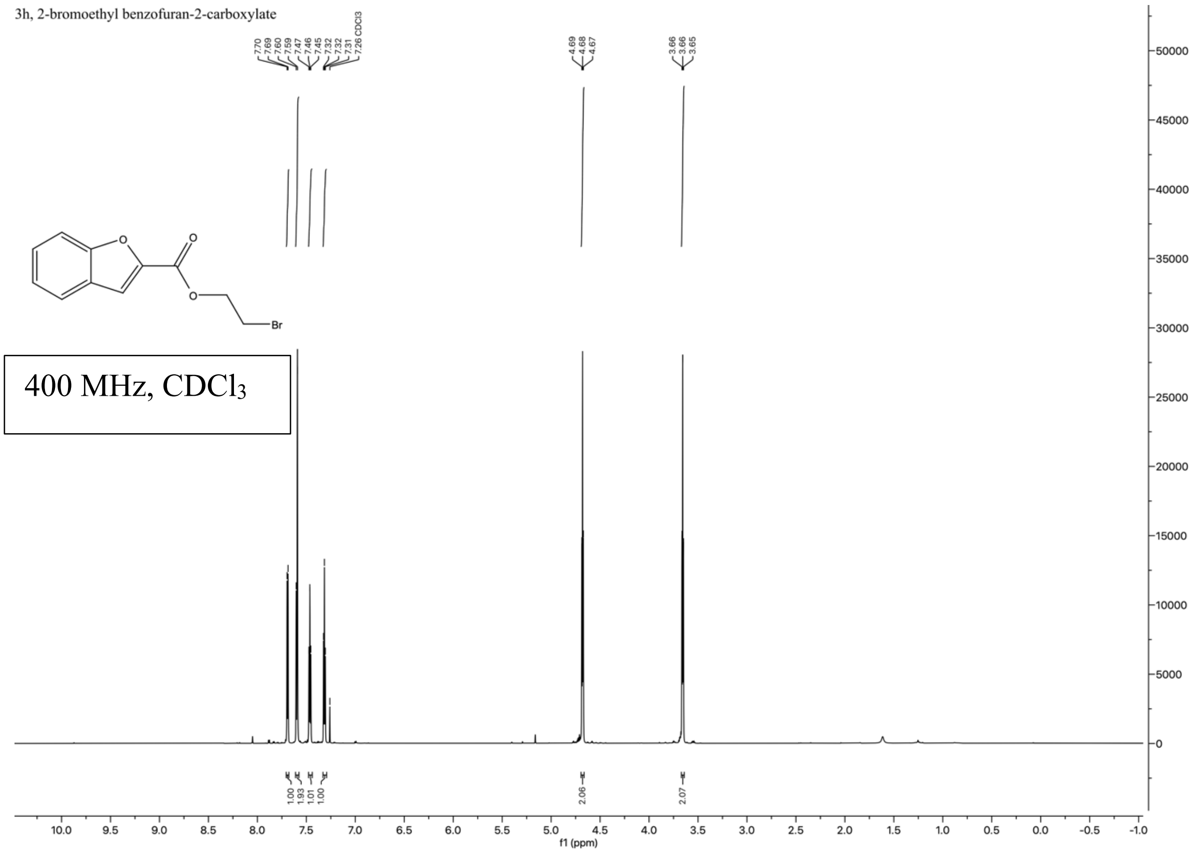

3h, 2-bromoethyl benzofuran-2-carboxylate

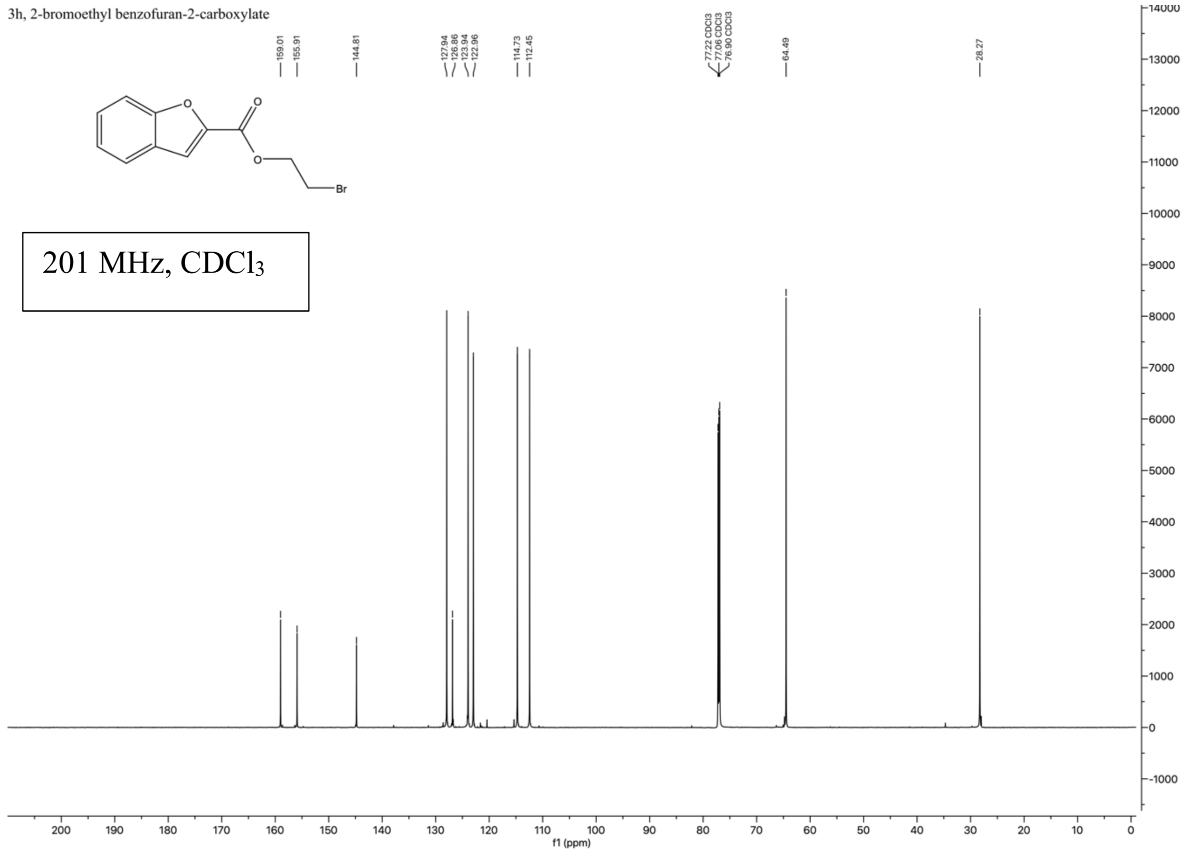

3i, 1-bromododecan-4-one

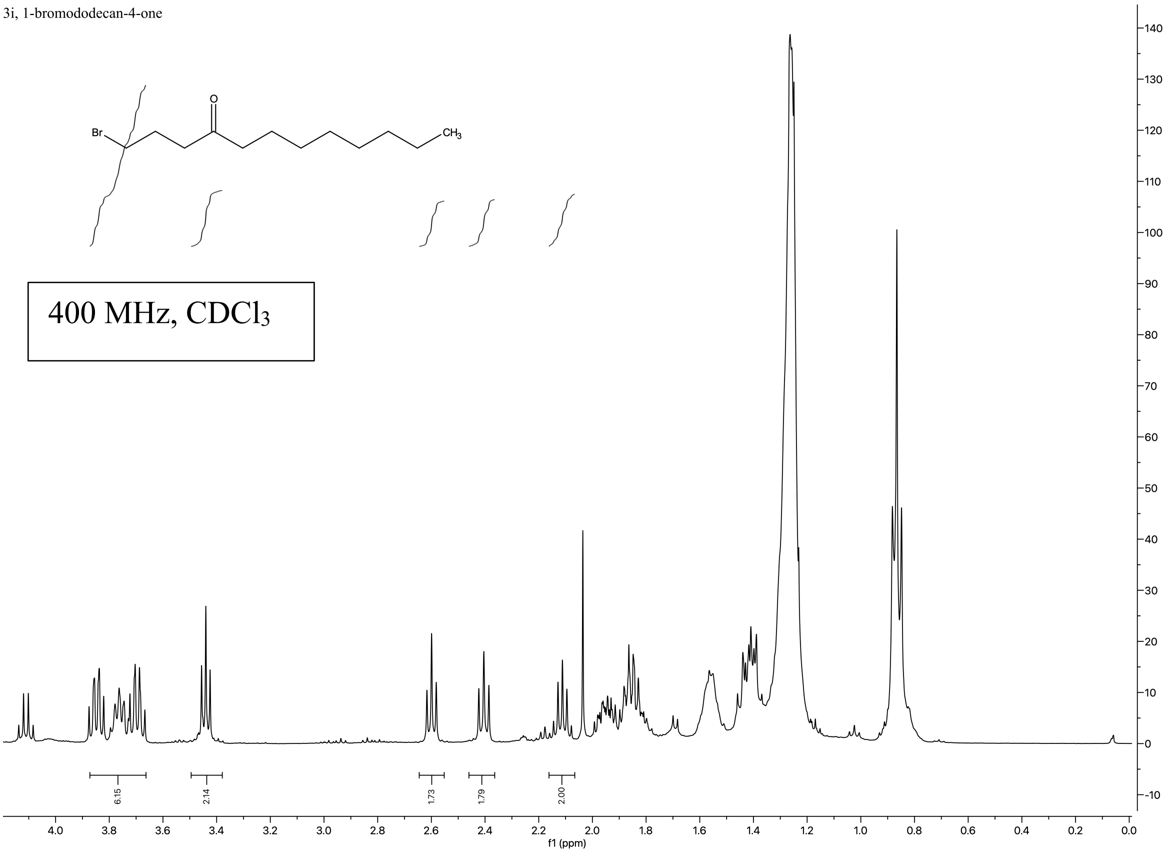

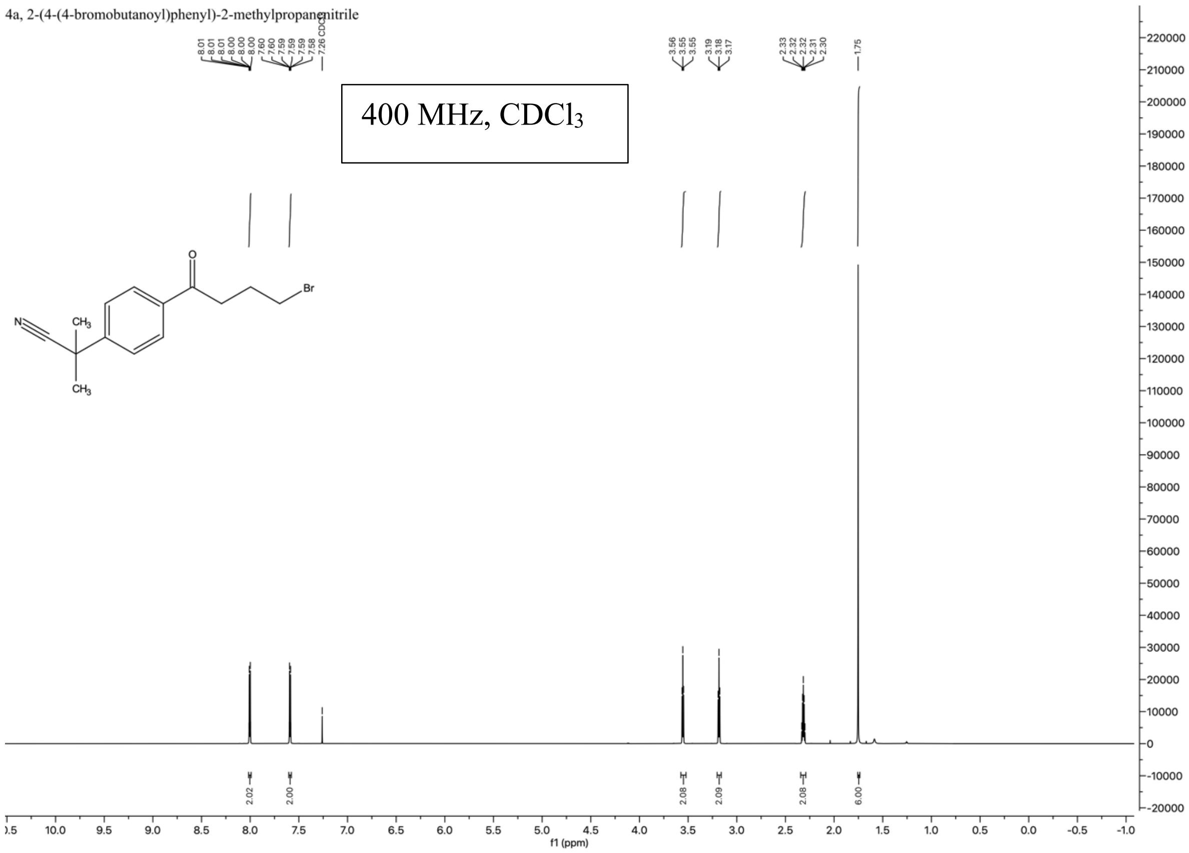
4a, 2-(4-(4-bromobutanoyl)phenyl)-2-methylpropanenitrile
400 MHz, CDCl3
8.01 8.01 8.01 8.00 8.00 8.00 7.60 7.60 7.59 7.59 7.59 7.58 7.26 CDCl3
3.56 3.55 3.55 3.19 3.18 3.17 2.33 2.32 2.32 2.31 2.30 1.75
f1 (ppm)

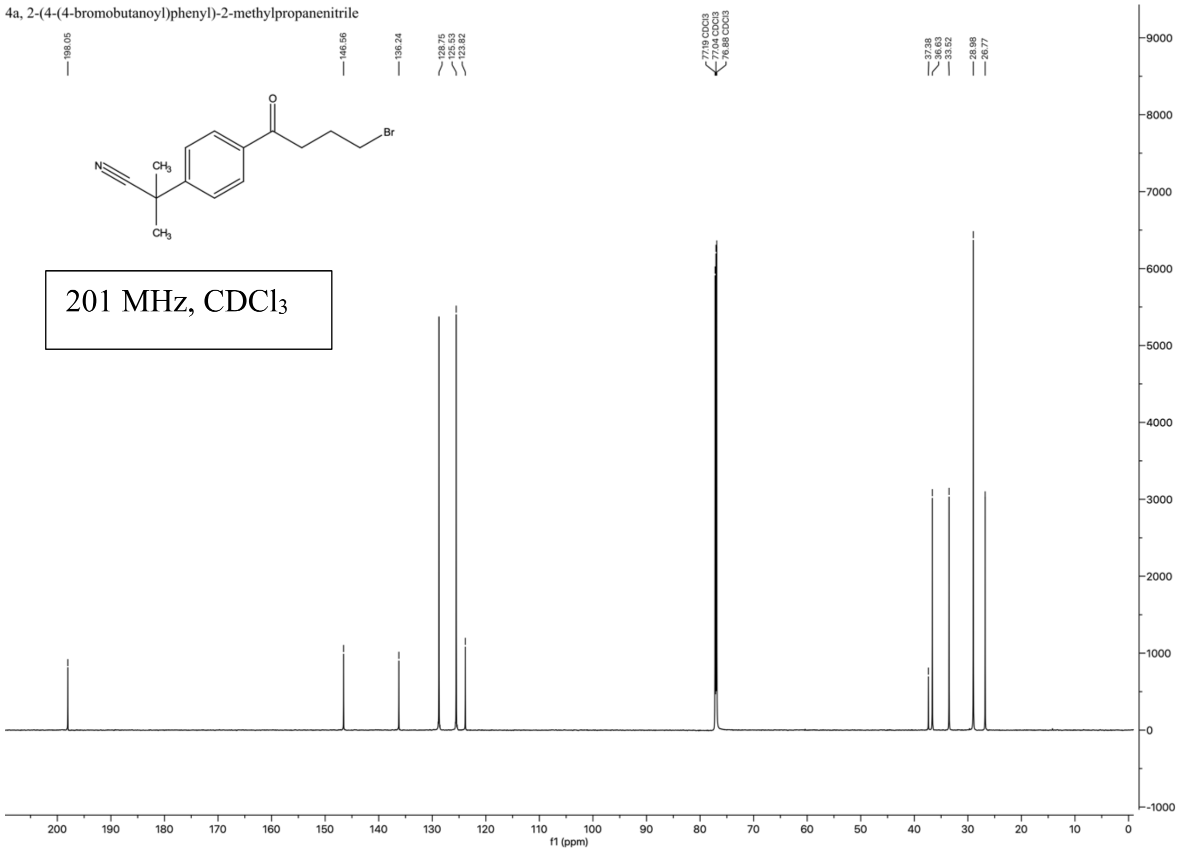
4a, 2-(4-(4-bromobutanoyl)phenyl)-2-methylpropanenitrile
201 MHz, CDCl3
198.05 146.56 136.24 128.75 125.53 123.82
77.19 CDCl3 77.04 CDCl3 76.88 CDCl3
37.38 36.63 33.52 28.98 26.77
f1 (ppm)
